# Identification and Validation of Immune Molecular Subtypes and Immune Landscape Based on Colon Cancer Cohort

**DOI:** 10.3389/fmed.2022.827695

**Published:** 2022-05-06

**Authors:** Wenqian Qi, Qian Zhang

**Affiliations:** Department of Digestive, China-Japan Union Hospital, Jilin University, Changchun, China

**Keywords:** colon cancer, immunotyping, immunosuppressants, prognosis, TMB

## Abstract

**Background:**

The incidence and mortality rates of colon adenocarcinoma (COAD), which is the fourth most diagnosed cancer worldwide, are high. A subset of patients with COAD has shown promising responses to immunotherapy. However, the percentage of patients with COAD benefiting from immunotherapy is unclear. Therefore, gaining a better understanding of the immune milieu of colon cancer could aid in the development of immunotherapy and suitable combination strategies.

**Methods:**

In this study, gene expression profiles and clinical follow-up data were downloaded from The Cancer Genome Atlas (TCGA) and Gene Expression Omnibus (GEO) databases, and molecular subtypes were identified using the *ConsensusClusterPlus* package in R. Univariate and multivariate Cox regression analyses were performed to evaluate the prognostic value of immune subtypes. The graph structure learning method was used to reduce the dimension to reveal the internal structure of the immune system. Weighted correlation network analysis (WGCNA) was performed to identify immune-related gene modules. Finally, western blotting was performed to verify the gene expression patterns in COAD samples.

**Results:**

The results showed that 424 COAD samples could be divided into three subtypes based on 1921 immune cell-related genes, with significant differences in prognosis between subtypes. Furthermore, immune-related genes could be divided into five functional modules, each with a different distribution pattern of immune subtypes. Immune subtypes and gene modules were highly reproducible across many data sets. There were significant differences in the distribution of immune checkpoints, molecular markers, and immune characteristics among immune subtypes. Four core genes, namely, *CD2*, *FGL2*, *LAT2*, and *SLAMF1*, with prognostic significance were identified by WGCNA and univariate Cox analysis.

**Conclusion:**

Overall, this study provides a conceptual framework for understanding the tumor immune microenvironment of colon cancer.

## Introduction

Colon adenocarcinoma (COAD) is the second most commonly diagnosed cancer worldwide and is the second leading cause of cancer-related deaths (1). Currently, the primary treatment methods of COAD include surgery, chemotherapy, and radiotherapy. The 5-year survival rate of early patients who had undergone complete treatment can reach 90%; however, treatment methods for late-stage patients with COAD are limited. At present, fluorouracil-based treatment is still recommended if the patient’s physical condition permits (2). Although several clinical studies on patients with advanced colorectal cancer have shown that chemotherapy combined with bevacizumab or cetuximab can improve patients’ prognoses (3–6), the 5-year survival rate of such patients is only 14% (7).

Immune checkpoint inhibitors (ICIs) have provided patients with advanced colon cancer with new treatment options. ICIs are effective in the treatment of a range of malignant tumors in recent studies (8, 9). Patients with colon cancer, particularly those with dMMR/MSI-H, who are more sensitive to ICIs than those with microsatellite stability (MSS)/(MSI-L), may benefit from immunotherapy (10, 11). In the keynote-016 study, 62% of patients with MSI-H colon cancer pretreated with ICIs demonstrated objective efficacy but did not reach the median of progression-free survival (PFS) or overall survival (OS) (6). In addition, MSS/MSI-L patients did not achieve objective response, with median PFS and OS time of only 2.2 and 5.0 months, respectively (10). Therefore, only dMMR/MSI-H is recommended as a biomarker for assessing the applicability and efficacy of ICIs (12). However, dMMR/MSI-H patients account for approximately 15% of all patients with colon cancer, while they account for approximately 5% of patients with metastatic colon cancer (13). Moreover, the effective rate of ICIs in patients with dMMR/MSI-H is only 30–40% (14), which greatly limits their applicability in colon cancer. In addition to the MSI status, other potential biomarkers of ICIs include programmed cell death ligand 1 (PD-L1) expression, tumor mutation burden (TMB), and BRAF and KRAS gene mutation status (15), but their effects are not ideal. First, there is temporal and spatial heterogeneity in PD-L1 expression. Additionally, the predictive efficacy of the TMB level of ICIs is not accurate. Some patients with lower TMB can also respond to immunotherapy (16). Therefore, it is urgent to analyze the tumor characteristics and immune microenvironment of colon cancer.

The tumor microenvironment (TME) contributes to the occurrence and development of colon cancer. Studies have shown that the TME can determine tumor progression by reprogramming the type and number of immune cell infiltration (ICI) (17, 18). The TME has an extremely complex constituent system, including tumor cells, stromal cells, various factors, and the extracellular matrix (19). As an important component of the TME, infiltrating immune cells, especially macrophages and lymphocytes, are highly associated with tumor prognosis (20). Therefore, the pattern of ICI may have potential prognostic value and can be used to guide immunotherapy.

In this study, we genotyped 424 COAD samples of The Cancer Genome Atlas-Colon Adenocarcinoma (TCGA-COAD) based on 1921 immune cell-related genes and identified three reproducible immune subtypes of COAD, which showed significant differences in prognosis. At the same time, independent data were used for subtype verification and comprehensive molecular identification. The findings revealed that distinct gene expression profiles were linked to different immune subtypes. The composition and functional orientation of tumor-infiltrating immune cells (immune activation and inhibition) and cytokine profiles showed a wide range of patterns, especially in clinical prognosis. This study provides a conceptual framework for understanding the tumor immune microenvironment of COAD, which may have clinical significance for the design and development of novel immunotherapies and their appropriate combination strategies.

## Materials and Methods

### Expression Profile Data Source and Preprocessing

RNA sequencing (RNA-Seq) data of TCGA-COAD were downloaded from the TCGA GDC API.

The RNA-Seq data of TCGA-COAD were preprocessed in the following steps.

1) Sample data of primary solid tumors were retained.
2) Samples without survival status were removed.
3) Samples with survival time > 30 days were retained.
4) Genes whose expression level (TPM) was equal to 0 in more than 50% of the samples were removed.
5) Log conversion log2 (TPM + 1) was performed.
6) The expression profiles of 19228 genes were obtained by matching ENSG with clinical information and Gene Symbol.

After screening, a total of 424 samples were included.

The GEO data were downloaded from Gene Expression Omnibus (GEO), and the GSE39582 chip data set with survival time was selected.

The GSE39582 data were preprocessed *via* the following steps:

1) Samples without survival status were removed.
2) Samples with survival time > 30 days were retained.
3) Probes with empty gene detection values were removed.
4) The probe annotation file was used to map the ChIP probe to the gene. When multiple probes matched to a gene, the median value was considered, and the probes matched to multiple genes were removed. The expression profiles of a total of 23520 genes were obtained.

Finally, a total of 512 samples were included.

### Acquisition of Immune-Related Genes

Expression data from a total of 2,006 immune-related genes were collected (21; Supplementary Table 1). The following categories of immune-related genes were collected for follow-up analysis from the literature: immune cell-specific genes derived from single-cell RNA-seq data; genes encoding co-stimulatory or co-inhibitory molecules; cytokine and cytokine receptor genes; genes involved in antigen processing and presentation pathways; other immune-related genes.

### Identification of Immune Subtypes and Immune Gene Modules

The expression profile of 2006 immune-related genes was obtained from the TCGA database. Among them, 85 genes were filtered out due to low expression levels or the absence of annotated gene expression profiles. Finally, 1921 immune-related genes were obtained. The consistent matrix was constructed using the *ConsensusClusterPlus* package in R (22). Using the PAM algorithm and the “1-Pearson correlation coefficient” as the metric distance, 500 bootstraps, each involving 80% of the patients in the training cohort. were performed. The consistency matrix and the consistency cumulative distribution function were calculated to identify the best categorization, with the number of clusters ranging from 2 to 10. The immune-related genes were grouped by consistent clustering, and the immune gene modules were obtained simultaneously using the same settings and parameters as previously reported.

### Assessment of Clinical, Molecular, and Cellular Characteristics Associated With Immune Subtypes

The prognostic value of immune subtypes with age and sex as covariates and OS as an endpoint in the training cohort was evaluated using the log-rank test and univariate and multivariate Cox regression analyses. Variance analysis was then performed to assess the correlation between immune subtypes and various immune-related molecular and cellular characteristics in the verification cohort.

### Elucidation of the Immune Landscape

Considering the dynamic characteristics of the immune system, the Graph Structure Learning method was used for dimension reduction to reveal the internal structure of the immune system and observe the distribution of immune cells in each patient. Simply, this method projects high-dimension gene expression data into a lower-dimensional space preserving the local structure information (23). This algorithm has been previously used to simulate the progression and definition of cancer using large and single-cell gene expression data (24, 25). The obtained immune landscape reflected the relationship between patients in a non-linear manifold, which may complement the discrete immune subtypes defined in a linear Euclidean space.

### Western Blotting Experiment

Colon adenocarcinoma and adjacent normal tissues were collected from 3 patients, immediately placed in liquid nitrogen, and preserved at −80°C. Take the tumor tissue and normal tissue adjacent to the cancer into small pieces and put them into the tube, add lysis buffer RIPA (1% Triton X-100, 50 mM Tris–HCl pH7.4, 150 mM Na Cl, 10 mM EDTA, 100 mM Na F, 1 mM Na 3 VO 4, 1 mM PMSF, 2 μg/ml Aprotinin) (1 ml lysate is added to 250 mg tissue). Use a homogenizer to homogenize at low speed for 30 s each time, and ice bath for 1 min between each time until the tissue is completely lysed. Centrifuge at 13,000 rpm for 25 min, take the supernatant, and quantify the protein by Coomassie brilliant blue method. After mixing with 3× sample buffer, boil for 5 min. The sample (30–50 μg/lane) was electrophoresed in a 12% SDS-polypropylene gel for 3 h, and then transferred to a nitrocellulose membrane (voltage: 2 mV/cm2; time: 120 min). After sealing with 5% skimmed milk for 1 h, cut the transfer film according to the molecular weight marked by the pre-stained Marker, and add the primary antibodies separately at 4°C overnight. After washing 4 times with TTBS, add secondary antibody (1:2000) for 30 min at room temperature. After washing 4 times with TTBS again, the color will be developed by ECL method.

Primary antibodies were as follows: CD2 (1:1000, ab219411, Abcam), FGL2 (1:1000, ab198029, Abcam), LAT2 (1:1000, ab75610, Abcam), SLAMF1 (1:1000, ab228978, Abcam). After rinsing 3 times (10 min each time) with tris-buffered saline, the membrane was incubated with horseradish peroxidase-conjugated secondary antibody against rabbit IgG (1:5000, Amersham Bioscience, Piscataway, NJ, United States) for 1 h at room temperature. After washout, the membrane was developed using enhanced chemiluminescence reagents (Pierce, Rockford, IL, United States) and visualized using a chemiluminescence system (PTC-200, Bio-Rad Laboratories, Hercules, CA, United States). All Western blots were repeated three times.

## Results

### Molecular Subtype Based on Immune-Related Gene Expression

We first extracted the expression profile of immune-related genes in colon cancer from the RNA-Seq data of TCGA-COAD and finally obtained 275 immune-related genes with significant differences in the prognosis (Supplementary Table 2).

**FIGURE 1:**
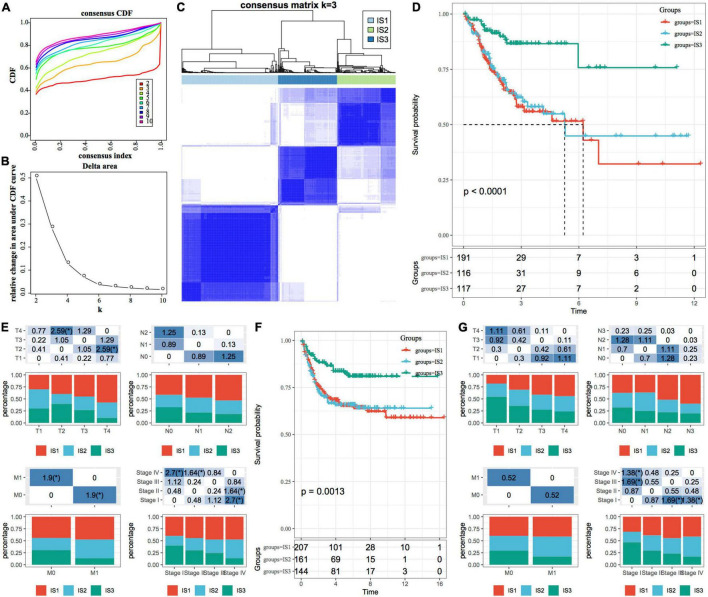
Immune subtypes in TCGA-COAD. **(A)** The cumulative distribution function (CDF) curve of samples in the TCGA-COAD cohort; **(B)** The CDF Delta area curve of samples in the TCGA-COAD cohort. The Delta area curve of consensus clustering, indicating the relative change in area under the CDF curve for each category number *k*, compared with *k*-1. The horizontal axis represents the category number *k*, and the vertical axis represents the relative change in area under the CDF curve; **(C)** Sample clustering heatmap when consumption *k* = 3; **(D)** The Kaplan–Meier (KM) curve for the prognosis of three subtypes; **(E)** The distribution proportion of different immune molecular subtypes and their association with different clinical features in the TCGA-COAD cohort; **(F)** Prognostic differences among the three immune molecular subtypes in the GSE39582 cohort; **(G)** The distribution proportion of different immune molecular subtypes and their association with different clinical features in the GSE39582 cohort. The lower part represents the proportion, and the upper part represents the statistical significance of the distribution difference between two pairs–log10 (*P*-value).

*ConsensusClusterPlus* is a popular machine learning algorithm, which was extensively utilized in medical studies (26–30). Four hundred and four COAD samples were clustered by *ConsensusClusterPlus*, and the optimal number of clusters was determined according to the cumulative distribution function (CDF). According to the CDF Delta area curve, when the number of clusters was selected as 3, it has relatively stable clustering results (Figures 1A,B). Finally, we selected *k* = 3 to obtain three immune subtypes (IS) (Figure 1C). By further analyzing the prognostic characteristics of the three immune subtypes, we observed that they had significant prognostic differences (Figure 1D). In general, the prognosis of IS3 was good, while that of IS1 was poor. In addition, we also compared the correlation between the three molecular subtypes and TNM stage, and clinical stage (Figure 1E). In addition, we used the same method for the molecular typing of GSE39582 data. We observed significant differences in the patient prognosis among the three immune molecular subtypes (Figure 1F), which was consistent with the results of the training set. Similarly, we compared the correlation between TNM stage, and clinical stage in the three molecular subtypes. We observed significant differences among the three immune molecular subtypes in terms of the stage (Figure 1G).

To investigate the pathways of different biological processes in different molecular subtypes, we used the R package “GSEA” for single-sample GSEA analysis (ssGSEA), and the most significant top 5 pathway enrichment results are shown in Supplementary Figure 1. It can be seen that IS1 subtypes are mainly enriched in ECM RECEPTOR INTERACTION and FOCAL ADHESION related pathways, while IS3 molecular subtypes are mainly enriched in APOPTOSIS, PRIMARY IMMUNOEFFICIENCY related pathways. The differences in the above pathways affect the prognosis between different molecular subtypes to some extent.

### Relationship Between Immunotyping and Tumor Mutation Burden and Common Gene Mutations

**FIGURE 2:**
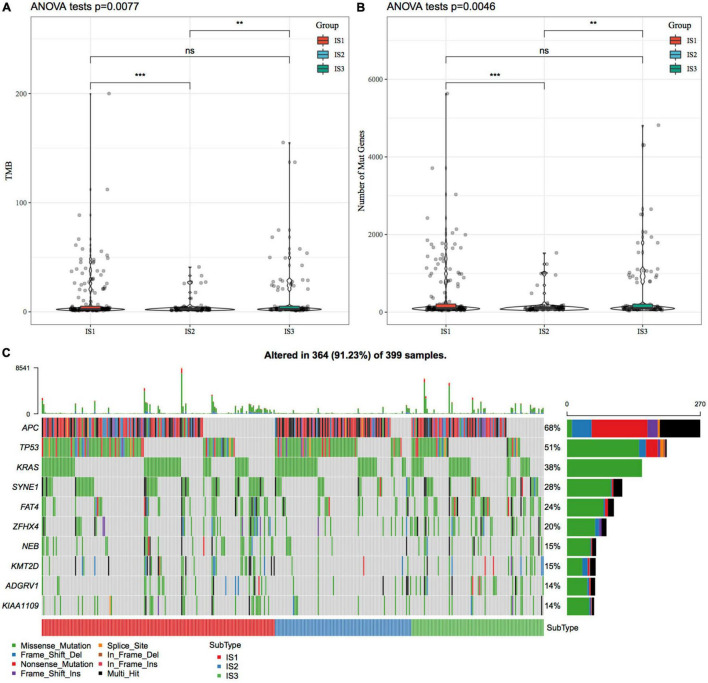
The relationship between immunophenotyping and TMB and common gene mutations. **(A)** Differences in the distribution of the tumor mutation burden (TMB) in three molecular subtypes; **(B)** Differences in the distribution of the number of gene mutations in the three molecular subtype samples; Rank sum test was used to determine the *P*-value, * *P* < 0.05; ** *P* < 0.01; *** *P* < 0.001; **(C)** The mutation characteristics of significantly mutated genes with the mutation frequency of the top ten mutated genes in each subtype sample.

We downloaded the mutation dataset processed by Mutect2 software of TCGA-COAD, calculated the TMB, and analyzed the TMB distribution in three immune molecular subtypes (Figure 2A). The TMB of IS1 and IS3 was significantly higher than that of IS2, and IS2 has the lowest TMB. In addition, we also counted the differences in the number of sample gene mutations in different immune molecular subtypes (Figure 2B). We further screened genes with mutation frequency greater than 3 in each subtype, and a total of 12460 genes were included. The Chi-squared test was used to screen the genes with a significant high-frequency mutation in each subtype, with the selection threshold as *P* < 0.05. Finally, 1553 genes (Supplementary Table 3) were obtained. Among them, the mutation characteristics of the top ten genes with a significant high-frequency mutation in each subtype are shown in Figure 2C.

### Expression of Classical Markers and Immune Checkpoint Genes in Response to Chemotherapy

**FIGURE 3:**
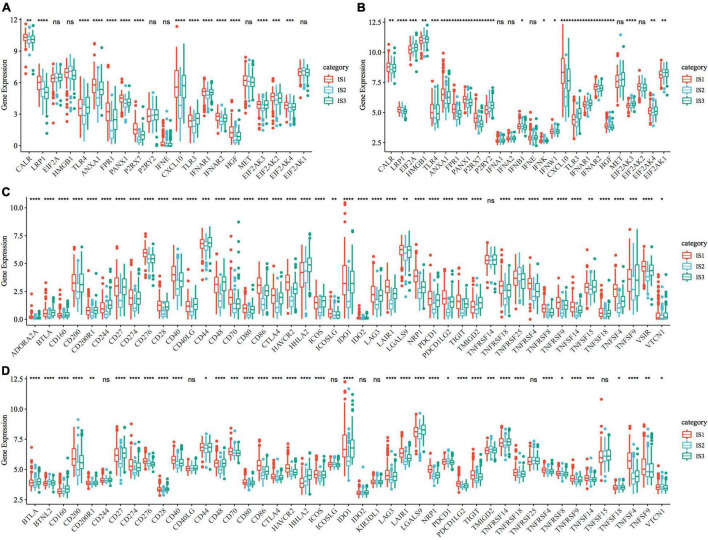
Expression of chemotherapy-induced marker and immune checkpoint genes. **(A)** Differences in the expression and distribution of classical markers in chemotherapy-induced immune response in the TCGA-COAD cohort; **(B)** The expression and distribution of classical markers of chemotherapy-induced immune response were different among samples in the GSE39582 cohort; **(C)** Differences in the expression and distribution of immune checkpoint genes in the TCGA-COAD cohort; **(D)** The expression and distribution of immune checkpoint genes were different among samples in the GSE39582 cohort. The significance was statistically tested by one-way analysis of variance, * *P* < 0.05, ** *P* < 0.01, *** *P* < 0.001, **** *P* < 0.0001.

To observe the expression and distribution of classical markers of chemotherapy-induced immune response in the three immune subtypes, we evaluated the differences in the expression of these genes in the TCGA-COAD cohort and GSE39582 cohorts. A total of 21 genes were expressed in the TCGA-COAD cohort (31), of which the expression of 15 (71.4%) genes was significantly different in each subtype (Figure 3A). A total of 26 genes were expressed in the GSE39582 cohort, of which the expression of 21 (80.8%) genes was significantly different in each subtype (Figure 3B). These results suggest that the expression of immune response markers induced by chemotherapy varies in different immune subtypes, which may lead to different prognoses. In addition, we obtained the expression profile of 47 immune checkpoint-related genes from a previous study (32), of which 2 genes were filtered out in the data preprocessing step. We analyzed the differences in the expression of these genes in each immune subtype and found significant differences in the expression of 45 genes in the TCGA-COAD cohort (Figure 3C) and 38 (84.4%) genes in the GSE39582 cohort (Figure 3D).

### Differential Analysis of Tumor Markers in Different Immune Subtypes

**FIGURE 4:**
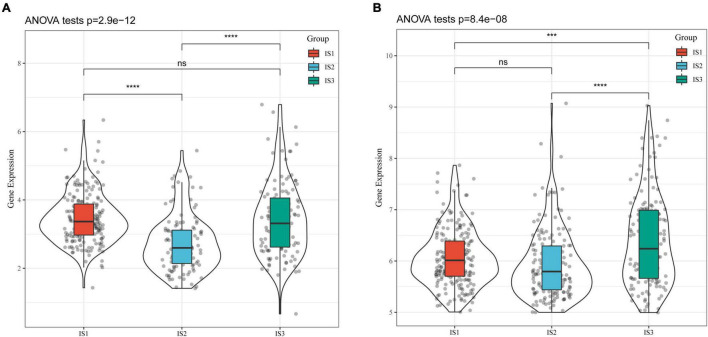
Differential analysis of tumor markers in different immune subtypes. **(A)** Differences in the expression of *CA199* in each subtype of the TCGA-COAD cohort; **(B)** Differences in the expression of *CA199* in each subtype of the GSE39582 cohort; * *P* < 0.05, ** *P* < 0.01, *** *P* < 0.001, **** *P* < 0.0001.

CA19-9 is the most significant prognostic indicator of metastatic colorectal cancer (33, 34). We extracted the expression profiles of *CA199* from the TCGA-COAD cohort and GSE39582 dataset, respectively, and analyzed their differential distribution in each subtype. We observed that the expression of *CA199* was significantly different among subtypes in each cohort (Figure 4). Among them, differences in the expression of *CA199* in the TCGA-COAD and GSE39582 were consistent.

### Immune Characteristics in Different Immune Subtypes

**FIGURE 5:**
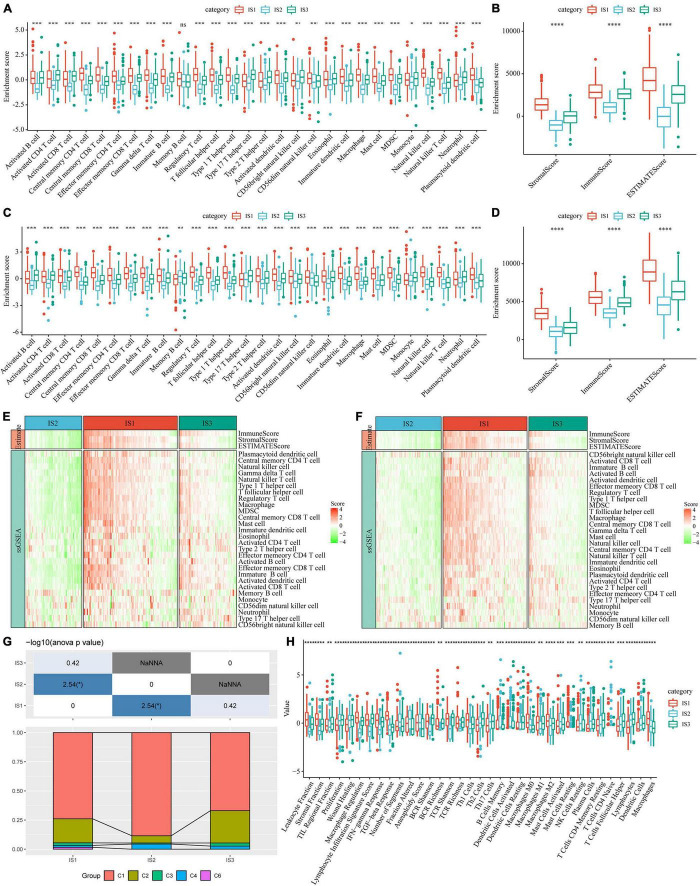
Immune signatures in different immune subtypes. **(A)** Differences in the enrichment scores of 28 types of immune cells in each subtype of the TCGA-COAD cohort; **(B)** The immune score of each subtype in the TCGA-COAD cohort; **(C)** Differences in the enrichment scores of immune cells in the GSE39582 cohort; **(D)** Immune scores for each subtype in the GSE39582 cohort; **(E)** A heatmap depicting the immune score of patients in the TCGA-COAD cohort; **(F)** A heatmap depicting the immune score of patients in the GSE39582 cohort; **(G)** Intersection of three immune molecular subtypes with previous immune molecular subtypes; **(H)** The distribution of three immune subtypes in 56 immune-related features, out of which 35 immune features were significant (FDR < 0.01). *P < 0.05, **P < 0.01, ***P < 0.001, and ****P < 0.0001.

To compare the distribution of immune cell components in different immune subtypes, we obtained the marker genes of 28 immune cells from the previous study (35). The ssGSEA method was used to score each immune cell to determine the score of 28 immune cells in each patient. Based on this, we calculated 28 immune scores of patients in the TCGA-COAD cohort (Figure 5A). The scores of most of these immune cell components were different among subtypes, such as activated B cells, activated CD4 T cells, activated CD8 T cells, central memory CD4 T cells, central memory CD8 T cells, and myeloid-derived suppressor cells (MDSCs), which were significantly lower in the IS2 subtype than in IS1 and IS3 subtypes. In addition, the immune scores of patients with cancer in the TCGA-COAD cohort were calculated using the ESTIMATE method (Figure 5B). The stromal, immune, and estimate scores of IS2 were significantly lower than those of IS1 and IS3. A similar trend was observed in immune subtypes in the GSE39582 cohort (Figures 5C,D). The heatmaps depicting ssGSEA and ESTIMATE immune scores of TCGA-COAD cohort and GSE39582 cohorts are shown in Figures 5E,F. To observe the correlation between the three immune molecular subtypes and the six molecular subtypes of a previous pan-cancer analysis, we extracted and compared the molecular subtype data of these samples from the previous study (36). The results showed that patients in our study mainly belonged to the C1 and C2 molecular subtypes. In addition, we observed that the proportion of “C1” subtype of the IS2 subtype was higher than that of IS1 and IS3 (Figure 5G). Moreover, we evaluated the correlation between immunophenotypes and 56 previously defined immune molecular characteristics. By selecting FDR < 0.01, 35 most significant immune-related features were identified (Figure 5H). The IS1 subtype had the highest leukocyte fraction, strategic fraction, macrophage regulation, IFN-gamma response, TGF beta response, and TCR Shannon. In contrast, the number of segments, Th17 cells, and activated mast cells were significantly higher in the IS2 subtype than in IS1 and IS3 subtypes.

### Differential Responses of Immune Subtypes to Immunotherapy/Chemotherapy

**FIGURE 6:**
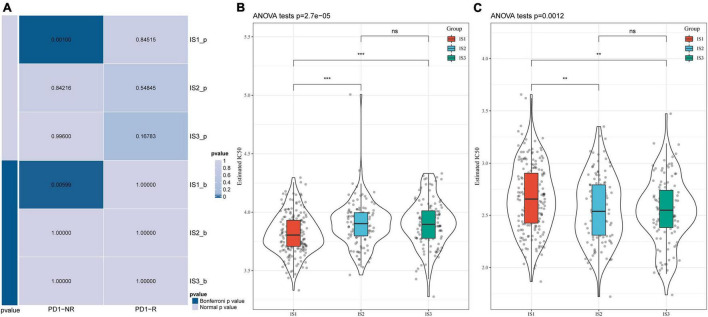
Differential analysis of immune subtypes on immunotherapy/chemotherapy. **(A)** Results of the submap analysis showed that IS1 was not sensitive to the programmed cell death protein 1 inhibitor (Bonferroni-corrected *P* < 0.05). The box plots of the estimated IC50 for **(B)** 5-Fluorouracil and **(C)** Cisplatin. **P < 0.01, ***P < 0.001.

We analyzed the differences in the response of different immune molecular subtypes to immunotherapy and chemotherapy. Here, we used the subclass mapping method to compare the similarity between the three immune subtypes and patients who received immunotherapy in the GSE77220 dataset. The lower the *P*-value, the higher the similarity. As a result, we found that the IS1 subtype was not sensitive to PD-1 inhibitors (Figure 6A). At the same time, we also analyzed the effects of different chemotherapeutic drugs on molecular subtypes and found that the IS1 subtype was more sensitive to 5-fluorouracil than other subtypes (Figure 6B), while IS2 and IS3 were more sensitive to cisplatin (Figure 6C).

### Immune Landscape of Colon Adenocarcinoma

**FIGURE 7:**
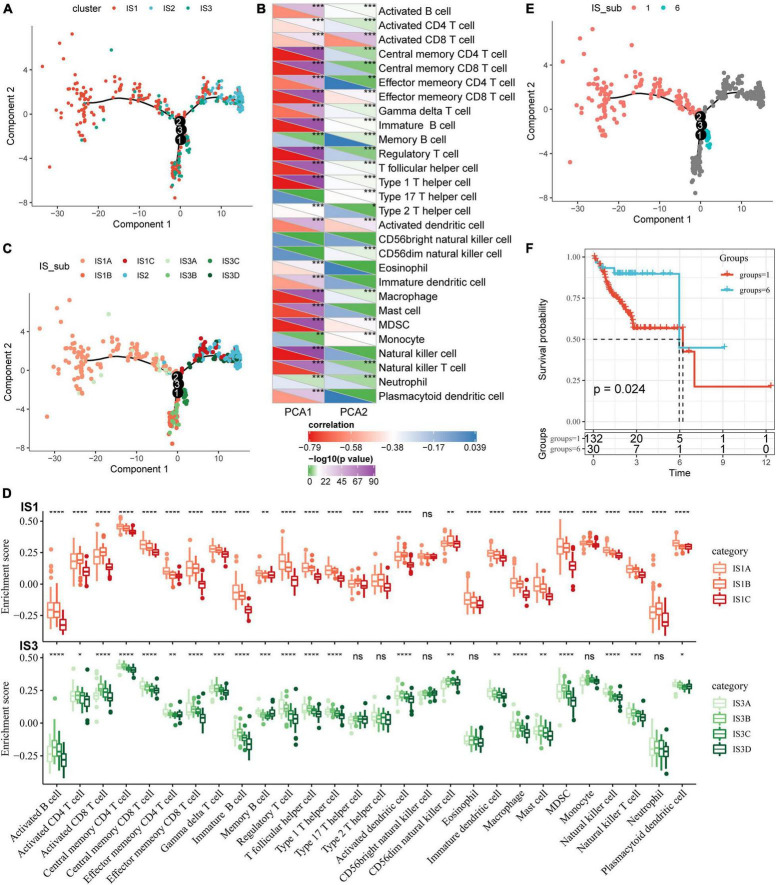
Immune landscape of COAD. **(A)** The immune landscape in colon cancer, where each point represents a sample, different colors represent different molecular subtypes, the horizontal axis represents the first principal component, and the vertical axis represents the second principal component; **(B)** The correlation heat map between the two principal components and 28 types of immune cells; **(C)** The immune landscape and molecular subgroups of three immune subtypes in colon cancer; **(D)** The immune landscape in colon cancer and samples from two different locations; **(E)** The immune landscape in colon cancer; **(F)** The prognosis of samples at different locations in the immune landscape of colon cancer is different. *P < 0.05, **P < 0.01, ***P < 0.001, and ****P < 0.0001.

To visualize and reveal the potential structure of individual distribution of patients, we applied the dimension reduction method based on graph learning to profile the expression of immune genes. This analysis puts a single patient into a graph with a sparse tree structure and defines the immune landscape of COAD. The patient’s position therein represented the overall characteristics of the immune microenvironment of the corresponding subtype of tumor (Figure 7A). The horizontal coordinates were highly correlated with a variety of immune cells (Figure 7B). Among them, the horizontal coordinates had the highest correlation with natural killer cells, regulatory T cells, type 1 T helper cells, central memory CD4 T cells, image B cells, MDSCs, central memory CD8 T cells, effector memory CD8 T cells, macrophages, and T helper cells (| R| > 0.75). The ordinates had the highest correlation with activated CD8 T cells, activated dendritic cells, effector memory CD8 T cells, and MDSCs. The IS2 subtype was distributed at both horizontal and vertical ends of the immune landscape, suggesting significant intra-class heterogeneity among subtypes. According to the position of IS2 in the immune landscape, it was further divided into three subtypes (Figure 7C). These subtypes showed different immune expression patterns (Figure 7D). Furthermore, different locations on the immune landscape map also had different prognostic characteristics. Results of immune landscape analysis provided further information on the immune subtypes defined earlier (Figures 7E,F).

### Identification of Immune Gene Co-expression Modules

**FIGURE 8:**
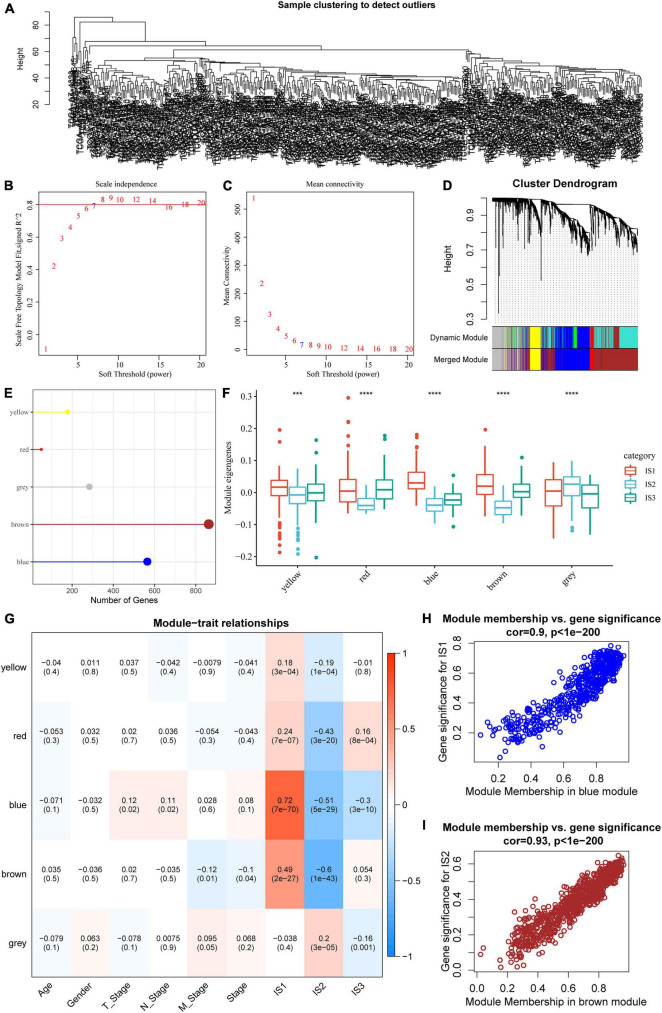
Co-expression module analysis of immune genes. **(A)** Sample cluster analysis; **(B,C)** Analysis of network topology for various soft-thresholding powers; **(D)** Gene dendrogram and module colors; **(E)** Gene statistics of modules; **(F)** Distribution of modular eigenvectors in immune molecular subtypes; **(G)** Correlation analysis between module feature vector and clinical features; **(H)** Scatter diagram for the module membership vs. gene significance for IS1 in the blue module; **(I)** Scatter diagram for module membership vs. gene significance for IS2 in the brown module. ***P < 0.001 and ****P < 0.0001.

The “WGCNA” package in R was used to identify the co-expression modules of these immune-related genes. First, the samples were clustered (Figure 8A), and the soft threshold was set to 3 to screen the co-expression modules. We found that the co-expression network conformed to the scale-free network, i.e., the log (k) of the node with connection degree k was negatively correlated with the log [P (k)] of the probability of the node, and the correlation coefficient was greater than 0.8. To ensure that the network was scale-free, we selected β = 7 (Figures 8B,C).

Next, the expression matrix was transformed into an adjacency matrix, which in turn was transformed into a topology matrix. Based on TOM, the average linkage hierarchical clustering method was used to cluster genes according to the standard of a hybrid dynamic cut tree. The minimum number of genes was set as 40 for each gene network module. After determining the gene modules using the dynamic cutting method, the eigengenes of each module were determined, modules were clustered, and modules close to each other were merged into new modules, and the following parameters were set: height = 0.25, deepSplit = 2, and minModuleSize = 40. A total of five modules were obtained (Figure 8D). Notably, the gray module was a gene set that could be aggregated to other modules. The gene statistics of each module are shown in Figure 8E, from which it could be seen that 1921 genes were assigned to five co-expression modules. The distribution of the eigenvectors of the five modules in the three immune molecular subtypes was calculated (Figure 8F). The results showed that the eigenvectors of the five modules were significantly different among the three molecular subtypes, in which the eigenvectors of IS2 in yellow, red, blue, and brown modules were significantly lower than those of IS1 and IS2. We further analyzed the correlation between each module and age, sex, T stage, N stage, M stage, as well as IS1, IS2, and IS3 subtypes. As shown in Figure 8G, the expression of genes in the blue module was significantly positively correlated with IS1, while that of the brown module was significantly negatively correlated with IS1. The results of correlation analysis of GS and MM of genes in the gene modules are shown in Figures 8H,I. The results showed that the GS and MM of blue and brown modules were highly positively correlated.

### Functional and Prognostic Analysis of Immune Gene Co-expression Modules

**FIGURE 9:**
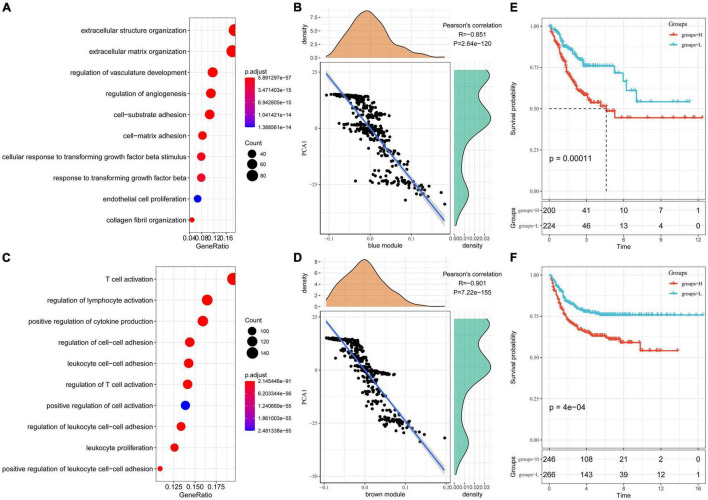
The function and prognosis analysis of immune gene co-expressed genes. **(A)** Graphical results of gene enrichment analysis of genes in the blue module; **(B)** The correlation between the feature vector of the blue module and the first principal component in the immune landscape; **(C)** Graphical results of gene enrichment analysis of genes in the brown module; **(D)** The correlation between the feature vector of the brown module and the first principal component in the immune landscape; **(E)** Kaplan–Meier (KM) survival curve distribution of patients grouped according to the expression of model characteristic genes screened by the brown module in the TCGA-COAD cohort; **(F)** KM survival curve distribution of patients grouped according to the expression of model characteristic genes screened by the brown module in the GSE39582 cohort.

We identified five immune-related gene modules. Results of functional enrichment analysis showed that the blue module was related to immune processes such as regulation of vascular development, regulation of angiogenesis, response to transforming growth factor-beta, and endogenous cell promotion (Figure 9A). The expression of the blue module was highly negatively correlated with the first principal component in the immune landscape (Figure 9B). The function annotated by the brown module was related to immune processes such as T-cell activation, regulation of lymphocyte activation, positive regulation of cytokine production, leukocyte proliferation (Figure 9C). Moreover, the expression of the brown module was highly negatively correlated with the first principal component in the immune landscape (Figure 9D). Next, we extracted the genes with correlation coefficient > 0.8 and the module feature vector in the brown module from the TCGA-COAD dataset for univariate Cox proportional-hazards regression analysis and selected *P* < 0.05 as the threshold for filtering. The expression of five genes was different among datasets. Then, we used lasso regression to further compress the number of genes in the risk model. The “glmnet” package in R was used for lasso Cox regression analysis. The model was found to be optimal at lambda = 0.0101564. Therefore, four genes (*CD2*, *FGL2*, *LAT2*, and *SLAMF1*) with lambda = 0.0101564 were selected as the hub genes of the module. Then, multivariate Cox analysis was conducted, and the risk score based on the final 4-gene signature was calculated as follows: Risk score = -0.19826556 × *CD2* - 0.15893408 × *FGL2* + 0.56282953 × *LAT2 -* 0.03863855 × *SLAMF1*. In the TCGA-COAD dataset, the prognosis of the high-risk group was significantly lower than that of the low-risk group (Figure 9E). Moreover, the prognosis of patients in the high-risk and low-risk groups was significantly different in the GSE39582 dataset (Figure 9F).

**FIGURE 10:**
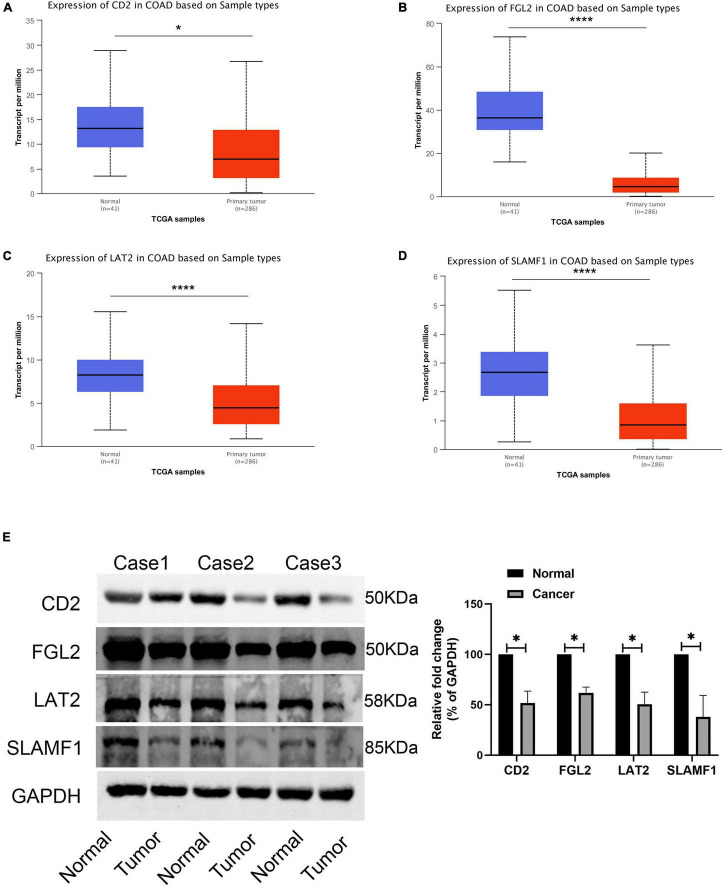
Expression validation of four core genes. **(A)** The expression of CD2 in TCGA-COAD; **(B)** The expression of FGL2 in TCGA-COAD; **(C)** The expression of LAT2 in TCGA-COAD; **(D)** The expression of SLAMF1 in TCGA-COAD; **(E)** The expression of four hub genes in 3 paired COAD tissues and normal tissues. *P < 0.05 and **** *P* < 0.0001.

Finally, four hub genes, *CD2*, *FGL2*, *LAT2*, and *SLAMF1*, whose correlation coefficient between the brown module gene and module characteristics was greater than 0.8 were selected as the final characteristic genes. The TCGA database showed that the expression of *CD2, FGL2, LAT2*, and *SLAMF1* in tumor tissues was significantly lower than that in cancer tissues (Figures 10A–D), which was confirmed by western blotting experiments (Figure 10E).

## Discussion

With the advent of ICIs in tumors, our understanding of cancers has shifted from focusing on tumor cells to knowing the entire tumor microenvironment. Because of this shift, tumor treatment strategies are changing as well. In the past, tumor cells were mainly eliminated by surgery and chemotherapy. At present, tumor progression can be inhibited by reactivating immune cells in the TME. However, biomarkers for assessing the efficacy of ICIs are unclear, which may be due to the complex TME characteristics of COAD. Previous studies have evaluated the relationship between the MSI status and ICI treatment prognosis in immunotherapy cohorts and have analyzed the mutation data of immune microenvironment and immunogenicity under different MSI statuses (15). However, studies on the systematic analysis of the immune microenvironment in patients with COAD are lacking.

In this study, we first identified molecular subtypes of COAD based on the expression of immune-related genes in the TME. The results showed that COAD could be classified into three immune subtypes (IS), with significant differences in the prognosis of each immune subtype. In general, the prognosis of IS3 was better than that of IS1, indicating that differences in the immune microenvironment can affect the prognosis, which is consistent with previous reports. Furthermore, we observed that most of these immune cell components were different in different subtypes. The results showed that the proportion of activated B cells, activated CD4 T cells, activated CD8 T cells, central memory CD4 T cells, central memory CD8 T cells, and MDSCs were significantly lower in the IS2 subtype than in IS1 and IS3 subtypes, which also explained the good prognosis of the IS3 subtype. For example, CD8-positive cell infiltration was previously reported to be positively correlated with the prognosis of COAD (37, 38).

In addition, we analyzed the differences in the efficacy of immunotherapy and chemotherapy among different immune molecular subtypes. The results showed that the IS1 subtype was not sensitive to PD-1 inhibitors. At the same time, we also analyzed the sensitivity of different subtypes to chemotherapeutic drugs and found that the IS1 subtype was more sensitive to 5-fluorouracil than other subtypes, while IS2 and IS3 were more sensitive to cisplatin. These results show that the IS1 subtype with poor infiltration of immune cells not only has a poor prognosis, but also exhibits poor response to PD-1 inhibitors, suggesting such patients should be considered for alternative treatment options. The application of classical chemotherapy containing 5-fluorouracil may benefit patients belonging to the ISI subtype. Additionally, we detected the expression profile of classical tumor marker genes, *CA199* and *CA153*, in colon cancer and analyzed their differential distribution in various subtypes. The results showed that differences in the expression of *CA199* and *CA153* in TCGA-COAD and GSE39582 datasets were consistent. The expression of *CA199* and *CA153* was relatively higher in IS1 and IS3 subtypes than in the ISI subtype, suggesting that these tumor markers could be easily used to categorize patients for treatment compared to the use of molecular typing and the application of ICIs. However, large-scale clinical trials are required to determine the applicability of these ICIs in patients with COAD.

Previous studies have shown that the TMB is an effective biological index for predicting the efficacy of ICIs. Because the TMB correlates with the frequency of gene mutations, higher TMB indicates higher gene mutations, leading to increased immunogenicity. This in turn can promote the level of lymphocyte infiltration in the TME and lead to a better prognosis of immunotherapy (39–42). Therefore, we calculated the TMB of the three immune molecular subtypes. The results showed that the TMB of IS1 and IS3 was significantly higher than that of IS2, and IS2 has the lowest TMB. This result may be in contradiction with our previous results. In our previous study, we found that the IS1 subtype was not sensitive to PD-1 inhibitors, indicating that ICIs are a better immune marker since higher mutations in immune genes do not necessarily lead to abundant infiltration of immune cells. Our results further defined COAD immune landscape also confirms this view. Although patients with COAD were categorized into three immune infiltration subtypes, the immune infiltration characteristics of each patient were different. Our classification categorizes patients with similar immune infiltration characteristics into different immune subtypes to facilitate early clinical decision-making.

To further simplify the clinical work, we identified the immune gene co-expression module, screened the core genes, and identified the following characteristic genes: *CD2* (CD2 antigen cytotoxic tail binding protein 2), *FGL2* (fibroleukin), *LAT2* (linker for activation of T-cells family member 2), and *LAMF1* (signaling lymphocytic activation molecule). The high expression of these genes is related to a poor prognosis. Among them, *CD2*, which is associated with malignancy in non-small cell lung cancer, shows stem cell characteristics (43). However, in breast cancer, high CD2 expression is associated with a longer survival time. High CD2 expression is mainly related to immune-related pathways. In addition, *CD2* expression is associated with a variety of tumor-infiltrating immune cells (TIC) (44). Fibrinogen like protein-2 (FGL2) plays a key role in cancer by regulating the proliferation, invasion, and migration of tumor cells, or regulating the function of immune cells in the TME (45). FGL2 is overexpressed in glioma, and its expression level is negatively correlated with the prognosis of patients with glioma. The expression level of *FGL2* in breast cancer cells was significantly lower than that in adjacent normal tissues. The low expression level of FGL2 is associated with a poor prognosis in patients with breast cancer. In addition, the expression level of FGL2 is positively correlated with the infiltration of breast cancer cells, especially those with high anti-tumor activity (46). LAT2 promotes the progression of multiple tumors and drug resistance (47, 48). SLAMF1 promotes methotrexate resistance by activating autophagy of choriocarcinoma cells (49). Moreover, it serves as a prognostic marker gene of chronic lymphoblastic leukemia (CLL) (50, 51). The functional differences of these hub genes could be attributed to the heterogeneity of different tumors, as well as their distinct immune microenvironment characteristics, which need to be investigated further.

In conclusion, in this study, we systematically analyzed the immune types of COAD according to the expression profile of immune-related genes and divided them into three subtypes with significant differences in prognoses. Immune-related genes were divided into five functional modules, with differences in the distribution and molecular and cytological characteristics of each immune subtype. In independent datasets, immune subtypes and gene modules were found to be highly reproducible.

## Data Availability Statement

The datasets presented in this study can be found in online repositories. The names of the repository/repositories and accession number(s) can be found in the article/Supplementary Material.

## Ethics Statement

The patients and their families in this study were fully informed, and informed consent was obtained from the participants. All research is conducted in accordance with the Helsinki Declaration. This study was approved by the Ethics Committee of China-Japan Union Hospital, Jilin University.

## Author Contributions

WQ designed the current study, collected the data, analyzed and interpreted the data. QZ supervised the study. Both authors wrote the manuscript, read and approved the final version of the manuscript, and agreed to be accountable for all aspects of the research in ensuring that the accuracy or integrity of any part of the work are appropriately investigated and resolved.

## Conflict of Interest

The authors declare that the research was conducted in the absence of any commercial or financial relationships that could be construed as a potential conflict of interest.

## Publisher’s Note

All claims expressed in this article are solely those of the authors and do not necessarily represent those of their affiliated organizations, or those of the publisher, the editors and the reviewers. Any product that may be evaluated in this article, or claim that may be made by its manufacturer, is not guaranteed or endorsed by the publisher.

## Supplementary Material

Supplementary Table 1 A total of 2,006 immune-related genes.

Supplementary Table 2 275 immune-related genes with significant differences in the prognosis.

Supplementary Table 3 1553 significant high-frequency mutation genes identified by Chi-squared test.

## Abbreviations

COAD: colon adenocarcinoma
TCGA: The Cancer Genome Atlas
GEO: Gene Expression Omnibus
ICIs: immune checkpoint inhibitors
PFS: progression-free survival
OS: overall survival
TMB: tumor mutation burden
TME: tumor microenvironment
ICI: immune cell infiltration
MDSCs: myeloid-derived suppressor cells.
