# Supplementary material for: Identification and Validation of Immune Molecular Subtypes and Immune Landscape Based on Colon Cancer Cohort
Source: Front Med (Lausanne). 2022 May 6;9:827695. doi: 10.3389/fmed.2022.827695 (PMC9121983; doi:10.3389/fmed.2022.827695)
Supplement: Supplementary Table 2 — 275 immune-related genes with significant differences in the prognosis. [file Table_2.DOCX]

p.value HR Low 95%CI High 95%CI

A2M 0.4633338142636 1.0654018796707 0.899461019761127 1.26195703901356

ABCA6 0.549365531590024 1.14916769137267 0.72897048262307 1.81157730576812

ABCA8 0.220927648703416 1.19865968836093 0.89678557286919 1.60215004787003

ABCA9 0.492976464542343 1.16528019720345 0.752518544714685 1.80444448516462

ABCB1 0.285053412082702 1.06479196840552 0.949025137382192 1.19468061626729

ABCC9 0.147839717523174 1.20049984089872 0.937305511260005 1.53759884123637

ABCD2 0.403111696872988 0.743435623496835 0.371028292351321 1.48963445019656

ABI3 0.991928181811785 0.998922508567782 0.810633663668138 1.23094589189409

ABI3BP 0.655078258118612 1.05019991623038 0.847113939958243 1.30197345602017

ACAP1 0.21730816365944 1.15821421517605 0.917169722812854 1.46260842990081

ACE 0.0759904189371573 1.21325106097967 0.979992275943318 1.5020303456488

ACHE 0.676672644097271 1.03075829701135 0.893961559145698 1.18848809100144

ACOXL 0.175897464691984 1.42754266815452 0.852574495420424 2.39026393628725

ACP5 0.867437634389092 1.01416241449596 0.859787191636462 1.19625578629364

ACSL5 0.0278801941499543 0.77871408620199 0.623106233372475 0.973181771537365

ACSM5 0.560088474806553 1.20530470652937 0.643168853023853 2.2587527812513

ACSS3 0.309429198736403 1.17748349479817 0.859275850830399 1.61353001970465

ACTA2 0.01444929237071 1.18712830226252 1.03466703648294 1.36205518910037

ACTN1 0.0036626023216365 1.54129960974386 1.15122225954266 2.06354982046674

ACVR1 0.502392714152725 1.13757328639675 0.780548637180828 1.65790178892301

ACVR1B 0.566008673479633 1.09829419038513 0.797383405001693 1.5127605127814

ACVR2A 0.231098743807628 0.812642490757528 0.578675376028937 1.14120601141939

ACVR2B 0.904468708588679 1.02096991060814 0.727489287962949 1.43284523306998

ACVRL1 0.547835704072325 0.940135934048498 0.768708551575767 1.14979282158035

ADAM12 0.0744017115983841 1.1217510114445 0.988741213739516 1.27265386957792

ADAM28 0.988999738930845 1.0015886863509 0.799252877782244 1.25514705609784

ADAM8 0.00377400672943169 1.26518168213531 1.07901747746529 1.48346502465456

ADAMDEC1 0.994899793946253 1.0003923561029 0.887020201807563 1.12825487413896

ADAMTS10 0.0124683696274469 1.3643660723554 1.06927127613391 1.74090038790249

ADAMTS12 0.160877862606687 1.12268189917782 0.955004122756881 1.31980021521058

ADAMTS14 0.0185676158076729 1.29003824508409 1.04356060568169 1.59473121610654

ADAMTS16 0.118610102686074 1.1749221626374 0.959591308539295 1.43857293826252

ADAMTS2 0.193120385688585 1.0940738870037 0.955522422976742 1.25271541665593

ADAMTS4 0.000295164265055525 1.35697252880438 1.15022765252282 1.60087826083038

ADAMTS5 0.14695139260677 1.2073109315739 0.935951983986806 1.55734451172271

ADAMTS9 0.368719993113035 1.11136048387475 0.882815105588124 1.39907226020503

ADAMTSL2 0.000161506812006607 1.37303697579115 1.16453928039997 1.61886384480067

ADAP2 0.449920395224115 1.10210865598905 0.856426699445901 1.41826905956091

ADAT1 0.971713722776689 0.994023302533282 0.713664633053942 1.38451911474292

ADCY4 0.00421678473176595 1.50116128665775 1.13653685941245 1.98276473824608

ADCYAP1 0.0383550887864597 1.29385634951525 1.01391317039726 1.65109232433091

ADORA2A 0.665053005316061 1.22774747911265 0.484964627650727 3.1081934362295

ADPRH 0.386006388372944 1.12979325347518 0.857376937372815 1.48876502266236

ADRA2A 0.694685377651022 1.02713767749686 0.898589172591526 1.17407580762518

ADRM1 0.208849118860736 1.17378372913679 0.914241297779542 1.50700722679287

AEBP1 0.0296405017461577 1.14324445894797 1.01331555338406 1.28983305205409

AFF3 0.0150798232358581 1.72372549189935 1.11115832387343 2.67399299234527

AGAP2 0.107544066953989 1.17113142223314 0.96616773053361 1.41957629591324

AGTR1 0.388778436699114 1.1262017607382 0.859472281579684 1.47570833064991

AHCYL2 0.227745413276899 0.89750889735764 0.752887780712044 1.06991007354948

AIF1 0.742291452865345 0.972607856350035 0.824213584238941 1.14771954784911

CRYBG2 0.809959888877662 1.02244512952097 0.853239819239186 1.22520541037724

AIM2 0.801311412532772 1.01843276779753 0.883388186682927 1.17412177133406

AKAP12 0.0350618202947353 1.13468038166642 1.00889254869029 1.27615133069428

AKAP2 0.992209602637055 0.995701008008921 0.419318809463884 2.3643597066813

AKAP5 0.00174857569404431 0.640109587291687 0.484094397365726 0.846405754688339

AKNA 0.49611183428167 1.11513639607173 0.814777130268924 1.52622003692397

ALDH1A1 0.777423046726007 1.01576890482181 0.911343562827566 1.13215971461041

ALDH3B1 0.0257343553937151 1.30791303029215 1.0330607751218 1.65589144027496

ALOX5 0.202378239067726 1.10038203472261 0.949887045888115 1.27472063923997

ALOX5AP 0.430489397708798 1.06557839880557 0.909921794270293 1.24786254285909

ALOXE3 0.079471101361545 1.46608303834597 0.956012791505463 2.24829572828313

ALPK2 0.881588882819 1.0308285996252 0.691318474062812 1.53707392710109

AMH 0.0495674719983253 1.14082585348128 1.00024920658192 1.30115937049205

AMHR2 0.317664131918371 0.331833399900219 0.038128907528939 2.88792447582625

JAML 0.213029915555384 0.860552209386456 0.679394340189787 1.09001512269449

AMIGO3 0.0446429028035919 33.5326006306665 1.08766568366333 1033.8059956701

AMPD1 0.265484347388397 0.777640296768488 0.499497297730128 1.21066607148075

AMPH 0.569568533462313 1.11910198775349 0.759349533369613 1.64929219543513

ANGPTL1 0.384787643009176 1.07407764928339 0.914209656782905 1.26190178383128

ANGPTL2 0.160032153931582 1.10958530410316 0.959754681454975 1.28280650344445

ANK2 0.241883282472959 1.17943563448882 0.894616253826055 1.55493308997326

ANKRD17 0.309065290804063 0.851302098123135 0.624259922120834 1.16091909249393

ANKRD22 0.00128483476229759 0.759604840311411 0.642522815429666 0.898021828281186

ANKRD44 0.632790019064333 0.925569585090408 0.673935191168689 1.2711594053411

ANKRD55 0.248402254274801 1.68981957836045 0.693352902291282 4.11837925243254

SOWAHD 0.860182088300265 0.978598622895508 0.769237606430393 1.24494077867167

ANO6 0.945434810405462 0.991586879144854 0.778491037778564 1.26301330545556

ANTXR1 0.372270739338889 1.05838922139749 0.934343682463701 1.19890332111695

ANTXR2 0.79288520667137 0.972014919040943 0.786421895355939 1.20140729602975

ANXA6 0.0015682764323251 1.39174252412061 1.13387916020355 1.70824839315143

AOAH 0.249910701278709 0.921959852794588 0.802785847345544 1.05882530562244

AOC3 0.0206982122286602 1.16120102941555 1.02309854150262 1.31794521839055

AP1S2 0.324231816134743 1.12704487525335 0.888529330687129 1.42958719196419

AP3B1 0.127506972650652 0.790412483081122 0.584100615017479 1.06959636293444

APBB1IP 0.803709397518031 1.02498566367691 0.84372600773978 1.24518576066843

APBB2 0.396266280248216 1.1193382813504 0.862659018075731 1.45239099324698

APLNR 0.327526071154983 1.08529438568831 0.921274524055202 1.27851565722443

APOBR 0.764622937898836 0.977927581464618 0.844996030672545 1.13177141652141

APOBEC3A 0.928248966654422 1.01167686088029 0.78578358875173 1.30250884020941

APOBEC3D 0.604814772679314 0.941216049204899 0.748236621502625 1.1839672448828

APOBEC3G 0.934519885007371 1.00884350722978 0.817720115963775 1.24463762381598

APOBEC3H 0.918508734125026 0.984332106814887 0.727375302485115 1.33206295731558

APOC1 0.207755949450058 1.07753315812824 0.959343843091708 1.21028316930035

APOC2 0.343159500986163 1.26298071093175 0.779379061967252 2.04665528498979

APOE 0.0189743896790061 1.12924954311407 1.02020454699587 1.24994985993578

APOL3 0.757755871853696 0.972435569435612 0.814180113218891 1.16145177381572

APOL6 0.0318500939134781 0.782802269314457 0.625945328084174 0.978966317584625

AQP10 0.332082019690959 0.58854007746652 0.201630211416498 1.71789445813156

AQP1 0.233776577245999 1.09093175274673 0.945322056640368 1.25896997831695

AQP9 0.397115892146381 1.05523710431325 0.93175995256854 1.19507749098875

AREG 0.445936924835673 0.956696899461128 0.853762144945633 1.07204209375765

ARHGAP15 0.900530111410565 0.979629117553669 0.709417980662666 1.35276132564691

ARHGAP18 0.391493831190429 0.903030104441709 0.715125884088304 1.14030744470621

ARHGAP22 0.114525261064767 1.25093243558965 0.947277949684219 1.65192482199327

ARHGAP25 0.66673978518708 1.05394875560725 0.829771180645406 1.33869192538366

ARHGAP30 0.645439064697626 1.04329752517071 0.870949754168259 1.24975031087391

ARHGAP31 0.694522973647737 1.04407156179915 0.84188625513232 1.29481318825711

ARHGAP4 0.0079132477779432 1.20362318856268 1.04975482857766 1.38004488344073

ARHGAP6 0.551287465011593 0.942820899233772 0.776810605061142 1.14430884727947

ARHGAP9 0.805214146134299 1.02782324914106 0.826405376519942 1.27833223438545

ARHGDIB 0.89557705490991 1.0137231842126 0.827037798276321 1.24254864330493

ARHGEF15 0.0416929937779477 1.26527296948348 1.00888986125747 1.58680917390744

ARHGEF37 0.870782459732009 0.978814316816164 0.756218671923447 1.26693177830087

ARHGEF6 0.789355814649349 0.971170252312615 0.783584856160482 1.2036624388052

ARID5A 0.00467000631340668 1.453071565836 1.12163345243843 1.88244829079489

ARL6IP5 0.306625632794548 0.84761284038225 0.617363400346469 1.16373521134824

ARRB1 0.257722748262557 0.847961943486959 0.637298602907443 1.12826146852013

ARRB2 0.397325075640494 1.13466362905115 0.846867417953871 1.520263412899

ARRDC5 0.177110202586032 1.50098501670016 0.832248470345257 2.70707138629373

ARSB 0.940489297518738 0.989425763904574 0.748472279172627 1.30794869699157

ART4 0.301979571513803 1.3026878241412 0.788457811311138 2.15229723495765

ASAH1 0.0638321298171546 0.765263936844565 0.576685155419072 1.0155088743515

CLMP 0.841428726546278 1.01697102592844 0.86240159308799 1.19924415245417

ASGR2 0.210574652257581 1.28663730826622 0.867170364321621 1.90900846146603

ASPN 0.1112172935423 1.09059426286012 0.980204037789366 1.21341659524871

ASRGL1 0.0498250646684302 0.83817392393213 0.702630297162503 0.9998651205291

ASXL2 0.530603737141586 0.914758819730432 0.692420202993604 1.2084911657067

ASXL3 0.755656562797016 0.915113684276424 0.523399690588043 1.5999876771213

ATE1 0.266198426431734 0.834964838969752 0.607556195356455 1.1474926725202

ATP10A 0.0471967480688966 1.25650979279282 1.0028305775278 1.57436050990426

ATP2A3 0.400953556667621 0.937327464604388 0.805933836226764 1.09014256060405

ATP8A1 0.134240516492637 0.865035314908361 0.715528627482931 1.04578079380403

ATP8B4 0.388357275436025 0.815652659475846 0.513370882155601 1.29592324776295

AVPR1A 0.361156371129426 1.12408681297359 0.874512216649581 1.44488680551782

AZGP1 0.359548947523517 1.06227593616935 0.933493105784917 1.20882538668097

B2M 0.360624230876779 0.903575807486042 0.727033005703816 1.12298786089316

B3GAT1 0.853043670877102 1.06110261536164 0.566528169207715 1.98743649746832

BANK1 0.343636288906334 1.14016180690365 0.869092649010153 1.49577717335705

BATF 0.125878270693258 1.12003833000719 0.968696041333079 1.2950252784753

BATF2 0.351807585794247 0.915273188256172 0.759651055879114 1.10277607416888

BATF3 0.614865888296526 1.06828454933115 0.82591993863765 1.38177058689503

BCL2A1 0.604751791556392 1.03585483032428 0.906494556566091 1.1836753146878

BCL2L14 0.117883575516693 0.817046508173869 0.634244878166142 1.05253510040051

BCL6B 0.0313912009929803 1.25242285946811 1.02029591012096 1.53736088066091

BDKRB2 0.599634490332478 0.93524075864799 0.72837933470333 1.20085130777732

BEND5 0.459725976458917 1.13597004492191 0.810182663715678 1.59276173232552

BEX5 0.566540896236821 1.03736639830783 0.915061445195134 1.17601834279957

BFSP2 0.415319069982649 0.719315918898246 0.325573390900361 1.58924348746541

BGN 0.0161662109173096 1.16801463747786 1.029163267041 1.32559938452233

BHLHA15 0.83091766387813 0.9792146034986 0.807504779794093 1.18743723095908

BHLHE22 0.496076831587656 0.904260423194355 0.67677353523759 1.20821348705454

BHLHE41 0.805348210700022 1.02147058417549 0.862678630169417 1.20949113359967

BICC1 0.549186896930193 1.05902553216598 0.877825584449003 1.27762860600993

BIN2 0.370265947057984 0.890670620187829 0.691388788265514 1.14739227353673

BIRC6 0.993911122295959 1.00111729384391 0.751504307705466 1.33363950912461

BLK 0.435275300942814 1.12621069049271 0.835494837837172 1.51808301133663

BMP2 0.315805366616611 0.91217912140216 0.762222180863986 1.09163806880935

BMP2K 0.0810407312013537 0.710239464344461 0.483585961303611 1.04312394708994

BMP7 0.356403255466816 1.0461136042916 0.950537983652387 1.15129925568989

BMPR1A 0.620224603484806 0.919294409487803 0.659033819251456 1.28233511942591

BMPR1B 0.768090537594604 1.0470122587922 0.771500892444798 1.4209117329564

BMPR2 0.97632645041736 1.00354540843675 0.794363111918997 1.267812379104

BNC2 0.612546955693018 1.06556509223366 0.833380731281157 1.36243726686773

BST1 0.347098585677734 1.10165733789123 0.900322867225384 1.34801517801033

BST2 0.273163367253601 1.06515622565011 0.951440778979927 1.19246285224139

BTK 0.967958260541179 1.00452543536015 0.805900429856034 1.25210424626001

BTLA 0.945578685863011 0.984254420489539 0.624019057042493 1.55244740255942

BTN2A2 0.0536574523661123 1.34724758462385 0.995319381559334 1.82361168475509

BTN3A1 0.232641198448127 0.859403783088497 0.670106137745991 1.1021759401744

BTN3A2 0.513964717483076 0.923178789310443 0.726173530882315 1.17363004955191

BTN3A3 0.368873743161585 0.900694345968173 0.716973724201776 1.13149237897417

VSIR 0.497740456498966 1.08376846321747 0.85891325480388 1.36748859712609

VSTM4 0.162957306276409 1.16754695213225 0.939220513542322 1.45138001755525

C10orf99 0.516031985224013 0.976313388035859 0.908180078625392 1.04955818134746

C11orf21 0.58425448350763 1.07025276829407 0.839205379427395 1.36491139847398

RUBCNL 0.844611388321392 0.989255020856629 0.887955870964858 1.10211050829212

MEDAG 0.0771003036444423 1.14142796873306 0.985720818953641 1.32173104468776

CEP128 0.537829612074366 0.875888253785034 0.574597235407141 1.33516171997416

SLIRP 0.685024720858196 1.06696686491891 0.780052338208491 1.45941270229309

RTRAF 0.29358788173654 0.834180517607839 0.59473176482948 1.17003526145269

C15orf48 0.00353909611437561 0.832723875004867 0.73633731354143 0.941727438295989

C16orf54 0.850242819765559 0.980646009621376 0.800578110957879 1.20121520064508

MILR1 0.34784727824663 1.11036590291061 0.892364464105493 1.38162431152206

SCIMP 0.652889010735245 0.94748279596929 0.748983346501097 1.19858959862263

LDLRAD4 0.290321753655893 0.854137047797379 0.637692169420359 1.1440474438994

PEAK3 0.882968885306051 0.968895918923548 0.636159091204161 1.47566750941147

C19orf38 0.755497449319214 1.03263826447508 0.843640206297192 1.26397696233371

TRIR 0.539305664780853 1.12115977327656 0.778193092456523 1.61527935598293

MCEMP1 0.102306624557941 1.13542019339127 0.974961992273571 1.32228643349918

C1orf116 0.187223155392357 1.18737110515182 0.919901545340561 1.53260981948617

C1orf127 0.169924517890726 1.27215398869451 0.902087355016827 1.79403442687782

GCSAML 0.0126972711534912 0.246536635713944 0.0819640479358857 0.741548450568152

C1orf162 0.42865618957219 1.08159932685661 0.890652371250584 1.31348339893156

THEMIS2 0.395982798830396 1.09083252080501 0.892427023754237 1.33334777721108

C1orf54 0.207435208789433 1.19453096964301 0.906100997202519 1.57477394003721

C1QA 0.515321050102842 1.04708314075736 0.911558606718384 1.20275657053503

C1QB 0.63369149943331 1.03182697814031 0.907074125035269 1.17373749667566

C1QC 0.469973759973979 1.05020734350774 0.919523497844725 1.19946414305101

C1QTNF7 0.961524155992014 0.992490435727439 0.730670882036547 1.34812716535921

LAMP5 0.0010984226919341 1.33958135812889 1.12389913068049 1.59665415343808

C2 0.383006237873127 1.08136053991725 0.90708629691628 1.28911727722644

RTP5 0.0450115226489169 2.57944985510537 1.02129852195241 6.51480582022535

TRABD2A 0.557347390779111 1.06412758388708 0.864629817869356 1.30965586819543

C3 0.567500410362336 1.03084029817274 0.928902513916405 1.14396473732924

C3AR1 0.943061805291691 1.00524720721123 0.870764453620004 1.16049976937501

C4A 0.131549237525415 1.16476127280721 0.955320845985962 1.42011851655062

NDNF 0.166833287340496 0.889920593525992 0.754300185463953 1.04992505376959

C5AR1 0.0348959880127506 1.181211269023 1.01188020019534 1.37887870698288

CREBRF 0.953359237467427 0.991784022171924 0.752236207313981 1.30761526375844

C5orf56 0.290462497627254 0.833723724347082 0.595101134041117 1.16802877490592

ADTRP 0.573749738137123 0.967319284823113 0.861574959768314 1.08604200735165

UQCC2 0.24433573645847 1.20536744822671 0.88014580839082 1.65076135271376

C6orf132 0.364124811431973 1.15729527480747 0.844162954677488 1.5865803464491

CCDC170 0.673842761367944 0.962243500664849 0.804351916518729 1.15112867335374

C7 0.0394011436057977 1.12787990562408 1.00585942243953 1.26470265439808

CPED1 0.378952944227549 0.909807653317276 0.7370599914769 1.12304286707527

NUGGC 0.917455945054009 0.980473525012985 0.67526703206999 1.42362693215526

ERCC6L2 0.617790964878897 0.917096674796489 0.652807263117928 1.2883838131728

C9orf139 0.882053499903713 1.04835816089203 0.56178612757303 1.95635808640726

CYSRT1 0.0495126201078907 1.22572272442912 1.00043393118783 1.50174454338842

CACNA1C 0.0999395998927484 1.20383097099447 0.965123464103553 1.50157887630633

CACNA2D2 0.609803568730754 0.95331062384969 0.793389245040243 1.14546693344525

CACNA2D4 0.293503670522356 1.14385925726321 0.890145118142609 1.46988841904442

CALB2 0.000144140061938393 1.24084437835126 1.1101762495438 1.38689219114409

CALD1 0.181109582025563 1.09809148347212 0.957370963808926 1.25949600693619

CALR 0.874013424087555 1.02776233602593 0.73263770302272 1.44177048900896

CAMK1 0.305243412939314 1.1950872569326 0.850033058429783 1.68020941952655

CAMK4 0.62473567657217 0.879267447249474 0.52507406963623 1.47238511383397

CANX 0.783291785974316 0.958618758599113 0.709331021903605 1.29551633294136

CARD11 0.127229294373559 1.09295037399428 0.974976573294688 1.22519920245631

CARD8 0.743708349857857 1.05770845995256 0.755610393569351 1.48058734471673

CARD9 0.0129108507648131 1.29423951771739 1.05611207184167 1.58605887943345

CASP5 0.0142324285242623 0.833678444641523 0.720830601186716 0.964192901793689

CASS4 0.514446701238258 0.898849197357244 0.652304984146976 1.23857689152306

CAV1 0.273405492492726 1.10720779351221 0.92274308308114 1.32854867242215

CCDC102B 0.367230498026694 1.14671004385467 0.851568307902175 1.54414380205921

CCDC141 0.360336865726954 1.31515033295061 0.731220918811569 2.36538692174071

CCDC69 0.822588457882632 1.02090703058432 0.851990068871526 1.22331374880575

CCDC80 0.114205917329975 1.10539185879638 0.976158363251318 1.25173456223183

CCL11 0.225069403729898 0.925843403033632 0.817477805352311 1.04857404241265

CCL13 0.645373658603765 1.02631132594822 0.918844205139618 1.1463476962447

CCL14 0.863161786605487 1.03655761843843 0.689073505130127 1.55927007546148

CCL15 0.0318734727373452 0.80209186646788 0.655768802662361 0.981064301384848

CCL16 0.597082258156631 0.756208522772533 0.268327283913126 2.13117101464404

CCL17 0.741509590336184 0.967426432303 0.794623275875956 1.17780831537662

CCL18 0.453721427885919 1.03193939568598 0.950462519821684 1.12040074612151

CCL19 0.972201111630111 0.998002184223739 0.891832910195394 1.11681050152897

CCL20 0.26661985013209 0.937485693144856 0.836567378383563 1.05057816926771

CCL21 0.771001787699007 1.01361759952629 0.925377639045419 1.11027173633596

CCL22 0.107415353471812 0.867040670241994 0.728797525398864 1.03150669102815

CCL23 0.110988086924419 1.20017546878538 0.958941505334688 1.50209491179701

CCL24 0.691143960728487 0.977713785337866 0.8748318278785 1.09269486497519

CCL25 0.658749711896955 0.975503947648198 0.873832740109384 1.08900468956805

CCL26 0.86168414480185 0.985178553960425 0.83284278277356 1.1653781521061

CCL2 0.696284509223272 1.02839503050234 0.8935169926875 1.18363315685905

CCL28 0.0126032443432456 0.836821823636447 0.727531267334592 0.962530128883344

CCL3 0.118069026578838 1.12202210538512 0.971176253369274 1.29629776325901

CCL3L1 0.0801386464630571 1.13841419581862 0.984557028355121 1.31631469170104

CCL3L3 0.0801386464630571 1.13841419581862 0.984557028355121 1.31631469170104

CCL4 0.983616438974046 0.998402232032113 0.857084630241211 1.16302052534319

CCL4L1 0.447157172580431 1.05818664161277 0.914602123852415 1.22431267026928

CCL5 0.199133445090249 0.915474108975777 0.8000216647505 1.04758768559895

CCL7 0.795594521211109 1.0291810406116 0.82790480834104 1.27939058172259

CCL8 0.755070221589718 1.02370480210257 0.883587710444384 1.18604130575876

CCNT1 0.952536497887044 0.990757892093544 0.729767777259563 1.34508706924793

CCR10 0.343724913654653 1.17328860697651 0.842820436479997 1.63333267167821

CCR1 0.833887397733327 0.982605896138974 0.833986997025985 1.15770911365539

CCR2 0.239017896658273 0.870085989033745 0.690172965577744 1.09689840963142

CCR3 0.0989777971032563 0.635314651831887 0.370632635109136 1.08901556041718

CCR4 0.149391415490157 0.832964910467335 0.64970924306923 1.06790929861517

CCR5 0.231430035620357 0.888201297902316 0.731445251388704 1.07855173589215

CCR6 0.215211052956768 0.558891059452442 0.222711770892847 1.40252675053335

CCR7 0.629295090086913 0.953487622088212 0.785830526983485 1.15691439089963

CCR8 0.395811746468051 0.886103366062365 0.670296575408641 1.17139070100183

CCR9 0.284235803737372 1.44962141738478 0.734704823995389 2.86019934143492

CCRL2 0.000221311977595351 0.630644003955683 0.493782016481937 0.805440146562739

CD14 0.218401223782401 1.10190327941472 0.944131275803925 1.28604026611768

CD160 0.250477149828651 0.66277659839853 0.328629092263373 1.33668269099158

CD163 0.261233372385645 1.07238907738584 0.949295127400481 1.21144447085241

CD163L1 0.787120540938929 0.970684629259693 0.782158494822874 1.20465181381735

CD180 0.903482780233547 0.984520914154813 0.765103595293942 1.26686299263284

CD19 0.261442219242984 1.13471094851163 0.910109116355195 1.41474128050559

CD1A 0.0217965976441886 0.690680068635503 0.503443625720951 0.947551886325316

CD1B 0.0542641529198868 0.707841075316298 0.497879008406423 1.0063468823653

CD1C 0.364655241502746 0.902624241911422 0.723287366301022 1.12642714368536

CD1D 0.381254919306957 1.11537521418225 0.873516402045498 1.42419978090726

CD1E 0.120437289105088 0.777848399454385 0.566491859080956 1.06806147843915

CD200 0.277625064857008 1.0925434151367 0.931204713846599 1.28183534319523

CD200R1 0.690569176638599 0.928395590819772 0.643971360721801 1.33844208861634

CD207 0.382396856669468 0.877800798418123 0.65521809203924 1.17599658963225

CD209 0.614193910012889 0.956144909230577 0.8031532740239 1.13827972445063

CD226 0.187939166564141 0.671002781711596 0.370506745632725 1.21521332167868

CD22 0.272805317071318 1.12393967534893 0.912119223827703 1.38495095906681

CD244 0.115601524283975 0.792466277591081 0.593131526403177 1.05879180782608

CD247 0.331385575599231 0.88715381969404 0.696738882405825 1.12960812102245

CD248 0.00362056780024649 1.25728489987881 1.07758096992907 1.46695734573646

CD274 0.442614168429119 0.920272360648682 0.744395996551136 1.1377025423265

CD27 0.723715494924457 0.970411700536121 0.821549641250954 1.14624706926231

CD28 0.709757427784003 0.94431622779121 0.698364798610402 1.27688729421096

CD2 0.0303092743903061 0.844343696000503 0.724484324261237 0.984032715549444

CD300A 0.54224898192957 1.05647594971515 0.88536801993952 1.26065252775086

CD300C 0.48691949357378 1.08677925952887 0.859510533781977 1.37414157537448

CD300E 0.16704383549589 1.14286580279575 0.945653258894191 1.38120630465265

CD300LB 0.218888242727403 1.25301335435471 0.874584505198907 1.79518669363365

CD300LF 0.490892350115358 0.936009974162859 0.775479748176108 1.12977118202369

CD302 0.286554771447687 0.871227412710718 0.676124212939483 1.12262982175814

CD33 0.728885298557634 1.06284872199643 0.752980360847213 1.5002348860446

CD34 0.0745339600263962 1.21534651157292 0.980867280090726 1.50587869854914

CD36 0.169471582596022 1.13790198203507 0.946384436646216 1.36817647309153

CD37 0.224299165803069 1.10995894397756 0.938061073644658 1.31335676527867

CD3D 0.0761212187814667 0.866423672574305 0.739468077670956 1.01517564187698

CD3E 0.233391980399671 0.902749215107966 0.762934489163795 1.06818626887778

CD3G 0.042721736971517 0.776259113638792 0.60760402776459 0.991728467838178

CD40 0.957251612191081 0.995551155720445 0.845783574261468 1.1718389122439

CD40LG 0.221838930434766 0.830990222148257 0.617446416146431 1.11838814065161

CD48 0.286905903895076 0.91308120440118 0.772375852700413 1.07941914925982

CD4 0.638836597154784 0.96257904404456 0.820851807477176 1.12877672631488

CD52 0.682874578223763 0.968077322857188 0.828541628790965 1.13111239129637

CD53 0.842074061932707 0.986100672582123 0.859258389738518 1.13166720055282

CD58 0.0241299916590937 0.715544506568693 0.53492770998112 0.957146042964769

CD5 0.773174194353401 1.02593638648685 0.861986147204649 1.22107005145153

CD68 0.937621652781284 0.990510681851763 0.780107169471878 1.25766234340167

CD6 0.34632765566281 1.1268997932002 0.878820912144202 1.44500788086194

CD69 0.360243104318111 0.918202271701792 0.764790442742914 1.1023874837329

CD70 0.00737518197975489 1.20702007534868 1.05181923219335 1.38512152820849

CD72 0.285417781928385 1.15217165104819 0.888477888791196 1.49412780016981

CD74 0.469647366095336 0.947833732442299 0.819710561276238 1.09598293202025

CD7 0.871599909759018 1.01294845911855 0.866627377089725 1.18397434463279

CD79A 0.393575285548591 0.952376087223623 0.851382534843512 1.06534979799896

CD79B 0.471659187563986 1.06754088024857 0.893497996314811 1.27548526768084

CD80 0.719034837028874 0.939715035561312 0.669692576052721 1.31861152361121

CD84 0.638352761780959 0.955669046056062 0.791020514430456 1.15458867239024

CD86 0.653284731166952 0.961817761552058 0.811565026942792 1.13988821071047

CD8A 0.289401594490578 0.916401501320855 0.779729911334676 1.07702898069619

CD8B 0.455049222452628 1.07303194087627 0.891855166029562 1.29101404577449

CD93 0.216304352878994 1.11515498513322 0.938208715105008 1.32547334174815

CD96 0.193164574242179 0.853820808391462 0.672964449718494 1.08328155097524

ADGRE5 0.185395913609965 1.25570824983529 0.896470297325864 1.75890178783163

CDC42SE2 0.00321141986470637 0.646389082044539 0.483557381526227 0.864052253876555

CDH11 0.510432083043652 1.05465828288495 0.90009164926086 1.23576759607895

CDH20 0.546188389701376 1.50347472077716 0.399880454946255 5.65277999476061

CDH3 0.857484251272572 0.980741860804063 0.793195541622063 1.21263238011456

CDH5 0.065182404942991 1.19898273338195 0.988653038141605 1.45405874405674

CDH6 0.958658703990038 0.990702154225082 0.695906610804374 1.41037711547494

CDK15 0.292723961380691 0.539454110172025 0.170860446630192 1.7032071653854

CDKL5 0.872430604906975 0.97498256862557 0.715633722031381 1.32832059174824

CDSN 0.700118981933242 1.27680530807297 0.368203356746563 4.42753105003713

CEACAM19 0.446952927615723 1.08919063177938 0.873937079556389 1.357461835763

CEACAM21 0.892892720988509 1.0202377555807 0.762147689249979 1.3657262136905

CEACAM4 0.353902565248 0.857264026066532 0.619020840751348 1.18720011025121

ADA2 0.702043811026817 0.972117921518438 0.841007984738135 1.12366739732153

CELF2 0.116166247804633 0.852828852409167 0.699213389478785 1.04019325494282

CERKL 0.281684946834681 1.14803598062193 0.892893919853447 1.47608420608227

CETP 0.145243968824108 1.25239334232744 0.925161761492559 1.69536739323906

CFP 0.479891183068272 1.11986153312835 0.81802938317031 1.53306211143699

CHAC1 0.549654220099693 1.06064394427538 0.874587723085571 1.28628100627703

CHIT1 0.305471791197918 1.08458737644831 0.9285770574044 1.2668090039174

CHN1 0.482515939487141 1.08188689929843 0.868525651371694 1.34766228380819

CHRD 0.0538083280309856 1.29182335096842 0.995814501574328 1.67582171927502

CHRDL1 0.0737666590327029 1.10649681296377 0.990317784173793 1.23630537254305

CHRNA6 0.724141075602019 1.11920664992628 0.598813265184629 2.09184331421413

CHST13 0.0807069458461576 1.14004704478796 0.984116003026314 1.32068502120986

CHST2 0.977779592781743 1.00338761375889 0.790889136702109 1.27298082211991

CIITA 0.32711783237654 0.918413271875165 0.774659703107183 1.08884318439853

CILP 0.0174064999848599 1.15965949168138 1.02638196042259 1.31024334848308

CISH 0.496206164540504 0.909447698512555 0.69190273369417 1.19539217877317

CLCF1 0.104328545535832 1.22638546939453 0.958693828702924 1.56882340796636

CLEC10A 0.0370311059699119 0.82285780271601 0.685083589730419 0.988339194866801

CLEC11A 0.0166968229171411 1.25473246380932 1.04194415219352 1.51097691025249

CLEC12A 0.536908263311683 0.920408330121652 0.707379952362816 1.19759047641602

CLEC14A 0.0166946910012023 1.27676648147085 1.04523772629887 1.55958076061765

CLEC1A 0.315402908363659 1.17163834001009 0.859985416471245 1.59623218427856

CLEC3B 0.436869823905609 1.07398417238996 0.897130719204087 1.28570115575515

CLEC4A 0.202871690739656 0.882438164208806 0.72792163314837 1.06975404795187

CLEC4D 0.759397861596649 0.940512686175981 0.635204582534636 1.3925656980123

CLEC4E 0.858627864895905 1.0162436428411 0.85113168887354 1.21338584277337

CLEC4G 0.0411984842273524 1.34862364741882 1.01202067411693 1.79718239843695

CLEC5A 0.887040552967219 0.987043559870553 0.824499685807648 1.18163172873449

CLEC6A 0.0623763732204011 0.574510508062774 0.320738491955 1.02906988762937

CLEC9A 0.93472673356267 0.966701070951281 0.42984751948709 2.17405223529834

CLECL1 0.18613315512607 0.720063288755154 0.442515837184091 1.17168945435322

CLIC2 0.463056060924855 0.930995248833896 0.769147292107733 1.12690009084747

CLIC3 0.998027851714338 1.00016324676891 0.878733665130374 1.1383728197541

CLIC5 0.473915267072525 0.932849851286698 0.771239471944332 1.12832508799345

CLIP3 0.0514661114151295 1.20250560026338 0.998827411207186 1.44771729574095

CLNK 0.551786681202364 0.767937021520359 0.321836672017126 1.83238058399441

CLOCK 0.213536065447756 0.84363966533662 0.645365227382521 1.10282961450514

CLTB 0.0402453084372437 1.32695361342765 1.01266503495554 1.73878413039707

CMA1 0.762747927528926 1.03629971665752 0.822137055437237 1.30625069827721

CMAHP 0.667323908334208 1.04248263448633 0.862335593241881 1.26026346554935

CMKLR1 0.733065881831471 0.967467984827808 0.800004254264121 1.16998665529316

CMPK2 0.505436021897772 0.93993512361755 0.783291277321759 1.12790485760385

CNFN 0.556721073882812 0.94762467849099 0.791993716450038 1.13383794925828

CNR2 0.133502178408511 1.57044366797268 0.870893726211123 2.8319107602316

CNRIP1 0.398800561809452 1.10764847595996 0.873497419388759 1.4045664235104

CNTF 0.195464450896527 0.693088485622877 0.397875170331652 1.20734261578222

CNTFR 0.330366080126598 1.07736778781403 0.927249861195093 1.25178918735375

COL10A1 0.137249941708587 1.07016862185188 0.978608571985951 1.17029516395121

COL11A1 0.0890388683860226 1.10078045542443 0.985455392989952 1.22960168432176

COL12A1 0.592572422421984 1.03860683992166 0.90405564216425 1.19318338122387

COL14A1 0.789354427302009 1.01969770461753 0.88373063498876 1.17658409433264

COL15A1 0.21941760026334 1.09618825465201 0.946738506463026 1.26922976242539

COL18A1 0.00349076205243702 1.28495806781631 1.08597982729469 1.52039402072444

COL1A1 0.0215682389623484 1.14345221270889 1.0199090081883 1.28196040259648

COL1A2 0.0518179289912395 1.13076325192383 0.999032266510009 1.27986409925284

COL6A5 0.0808682260064162 0.213026383878737 0.0375342033650627 1.20903698919828

COL3A1 0.0730056217251037 1.11943594148466 0.989534833209562 1.26638980764639

COL4A1 0.0580565639386395 1.19017852508857 0.994074759194039 1.42496820131563

COL4A2 0.0112537355122834 1.26196059073638 1.05417436575465 1.51070314770145

COL5A1 0.0368372420976061 1.16049216047459 1.00913940200634 1.33454511026468

COL5A2 0.120245747511384 1.11466905452524 0.972016966889675 1.27825659781638

COL5A3 0.00484395002134731 1.26411533327575 1.07392296509244 1.48799087808437

COL6A1 0.0240211629087908 1.18778741463803 1.02289271062031 1.37926385409173

COL6A2 0.0252058598198358 1.17576116420606 1.02033420874261 1.3548642233203

COL6A3 0.164323183996555 1.09770654804785 0.96255729846594 1.25183162347583

COL6A6 0.320510323396605 0.62997874798262 0.253164406318232 1.56765016331274

COL8A1 0.144791076306514 1.09906196079641 0.968018299697529 1.24784541164882

COLEC12 0.136409270424886 1.13546860623317 0.960644766376629 1.34210792674595

CORIN 0.106954561641313 1.28519179156323 0.947286011974011 1.74363172286219

CORO1A 0.171215678553438 1.14358037188216 0.943638172158389 1.38588720289139

COTL1 0.67095914989588 1.04759372639892 0.845342923612084 1.29823363387375

CPA3 0.135668892514258 0.915255330641521 0.814753472995557 1.02815434119946

CPNE5 0.647811662311367 1.056884412887 0.833556758289042 1.34004631489793

CPVL 0.103491979266573 1.12533013108739 0.976223905399681 1.29721050358288

CPXM1 0.101136359716866 1.14295738832383 0.974207931746527 1.34093713359738

CPZ 0.234998188019942 1.22593712456012 0.875914786704548 1.71583110159515

CR1 0.534147472393411 1.10571142483135 0.805479310827648 1.51785122046936

CR1L 0.351189817087325 0.506270017709166 0.121031236904767 2.11771223186718

CR2 0.104993479369989 1.12603412200264 0.975493753262242 1.29980621574867

CREB1 0.858144872777609 0.971300068711279 0.70578924196649 1.33669340276418

CREB3L1 0.881190644314088 1.00918650382444 0.895141120237881 1.13776183048182

CREBL2 0.875828310072083 0.974832829005214 0.708077973673345 1.34208248221079

CRISPLD2 0.0870631722400835 1.14800009599814 0.980125927459515 1.34462744376914

CRLF2 0.286504205105484 0.658369104012462 0.305245144086046 1.42000580686055

CRTAM 0.266583379231512 0.829069982676323 0.595620745981141 1.15401795658186

CRYBB1 0.0986927766961853 1.25696754570904 0.95813954195809 1.64899510121138

CSF1 0.645182901990513 1.0503620073608 0.852137471450809 1.29469760862488

CSF1R 0.700665791425496 1.02977889802573 0.886684521355116 1.19596604347893

CSF2 0.568447670133581 1.05195395475123 0.883904247681671 1.25195362033749

CSF2RA 0.65499135380582 1.04603586308064 0.858637164712486 1.27433457555642

CSF2RB 0.599916206859358 0.958779310437155 0.819232597529667 1.12209617744008

CSF3 0.0419039655177325 1.1490293809748 1.00510055239548 1.31356859291015

CSF3R 0.535353930528394 1.05364160956456 0.893178878764273 1.24293203500481

CSGALNACT2 0.382759318544372 1.10524756425877 0.88281391738465 1.38372555557219

CSMD2 0.964094829895532 0.990518680834889 0.654221741982972 1.49968610659293

CST7 0.487967827538796 1.05161628280377 0.912196392823238 1.21234507717716

CTF1 0.0128801690890951 1.24821764806903 1.04811151587363 1.48652817315178

CTHRC1 0.078639442120536 1.11603465647291 0.987510816931596 1.26128578350032

CTLA4 0.325239795816754 0.899819431819728 0.729150270679603 1.11043641131165

CTSB 0.769844657425105 1.03757306992033 0.810420452542584 1.32839425763052

CTSE 0.492454654345111 0.975764448488789 0.909747448666511 1.04657205725635

CTSG 0.475894336010281 1.05961845212099 0.903667088452652 1.24248329769079

CTSK 0.797101147813234 1.0197077841805 0.878743974385581 1.18328431878619

CTSL 0.0345334788130662 1.19862952665875 1.01327190505841 1.4178945799305

CTSO 0.1294284860823 0.849046447025912 0.687182505273311 1.04903699333936

CTSS 0.0509565608068887 0.788885684276794 0.621727084423353 1.00098682918741

CTSW 0.376186172198013 0.929884996963327 0.791601806316885 1.0923245761662

CTSZ 0.00375719489969343 1.52910446178739 1.14734300477518 2.03789141113583

CTTNBP2 0.639615237184582 0.963531784815115 0.824768204695582 1.12564171977467

CX3CL1 0.0264736317398509 1.19323956344561 1.02085184090473 1.39473780495904

CX3CR1 0.286431559091543 0.822756560200549 0.574758500562404 1.17776136706227

CXCL10 0.00742650028426282 0.869882247102891 0.785485099067085 0.963347522089847

CXCL11 0.00182254682910461 0.837529600119436 0.749193530340052 0.936281217962251

CXCL12 0.428189234437065 1.0654147487477 0.910842710341908 1.24621800664466

CXCL1 0.0412427411245396 0.87840153387216 0.77557524753368 0.994860598198058

CXCL13 0.296252538360863 0.943914709160774 0.847022967542872 1.05188998682609

CXCL16 0.44266147430729 0.915469879851808 0.730669370886825 1.1470100353306

CXCL2 0.291446289667478 0.931378274750523 0.816143498799623 1.06288353941816

CXCL3 0.0758305435494649 0.884163357276305 0.771803972223421 1.01288004530224

CXCL5 0.430122863493923 1.03472279797225 0.950601810819626 1.12628784887374

CXCL6 0.416621220867687 1.06279453410842 0.917551948817633 1.23102808858534

CXCL9 0.0210504973106904 0.887986115679447 0.80274008292605 0.982284749959485

CXCR1 0.445364895359641 1.07135939254222 0.897543536208431 1.27883595801629

CXCR2 0.715061038775113 1.03564186175067 0.858129321779265 1.24987462680629

CXCR3 0.395331545699381 1.08039138969757 0.903956183330002 1.2912634223406

CXCR4 0.375294082845447 1.06994995831996 0.921406119450334 1.24244118759685

CXCR5 0.052634935419202 2.43995280523151 0.989897604782142 6.01412677735222

CXCR6 0.0115607517406717 0.764797547609218 0.621101276883287 0.941738989435354

ACKR3 0.289235180974486 1.11172326257504 0.913969682736046 1.35226434300385

CXorf21 0.886044477602611 1.01749102800806 0.802675667666449 1.28979615775161

CXorf65 0.392292250076545 1.13253415737164 0.851558012456649 1.50621989206963

CYBA 0.545881380845664 1.05413241594053 0.888370177407009 1.25082446326598

CYBB 0.398192380732822 0.944371797171129 0.826946949056862 1.07847074387223

CYFIP2 0.419591681463888 1.11420056303525 0.856883138894634 1.44878903355422

CYP1B1 0.117696610486832 1.11438499287488 0.97299657982759 1.27631888753894

CYP27A1 0.808196255971196 1.01980787608317 0.870446576618374 1.19479831624088

CYSLTR1 0.120065306534218 0.736490625265392 0.500826502918432 1.08304659985646

CYSLTR2 0.596229765547308 0.872907809822923 0.52795481155772 1.44324481521758

CYTH4 0.633827656816846 1.04969631120265 0.859796280117568 1.28153886127744

CYTIP 0.448838280439979 0.930534601786648 0.772351575060609 1.12111462329094

DAAM2 0.188318287901977 1.1622906632615 0.928982391660026 1.45419288678967

DAB2 0.889193148332002 0.985494325794874 0.802385905930264 1.21038898988126

DACT1 0.0576301240337724 1.17847854596288 0.994697428697673 1.39621521402051

DACT3 0.0461421717083163 1.21106459115778 1.00328266463115 1.46187858682417

ACKR1 0.111346092225897 1.09745489716963 0.978747501082488 1.23055972044837

DBH 0.302267146709012 1.13061978383376 0.895400664815052 1.42763027304692

DCBLD1 0.119886739233363 1.31837510845029 0.930599563344124 1.86773451766447

DCHS1 0.0118382727480696 1.28981569105848 1.0579335450712 1.57252270206509

DCN 0.883715171586717 1.00951695403798 0.889179339419657 1.14614053128503

DDI2 0.671258598934272 0.941946972530872 0.714630338179729 1.2415707137764

DDR2 0.264941693346455 1.08865864486373 0.937627852981731 1.26401710579264

DDX58 0.965199347514479 0.995045188152368 0.796042704273347 1.24379624503812

DDX60 0.325226712562106 0.911135985844504 0.75693949350309 1.09674391655645

DENND1C 0.528624120103875 1.09766571506662 0.82146101513099 1.46674035631573

DENND2A 0.451171324974234 0.909206463477254 0.709794240419528 1.16464229512516

DERL3 0.538397061595554 0.95252095874422 0.815784098856701 1.1121768346779

DHRS1 0.141409015985911 1.27412587284748 0.922540876611443 1.75970169020826

DHRS9 0.520254395435589 0.96453272774544 0.86399091911831 1.07677449184471

DIXDC1 0.514428008390727 0.916480145615483 0.70510646588196 1.19121848678096

DKK2 0.260034096583047 1.11131790002194 0.924851866125493 1.3353786915986

DLC1 0.253025546845302 1.13911377771112 0.911110588287831 1.4241742059103

DLL4 0.450687007358993 1.1048160933267 0.852709995138654 1.43145806549998

DMKN 0.831115604544751 1.01436565485036 0.889747227378343 1.15643819961293

DMXL2 0.835073022236325 0.972171248236826 0.74534080489815 1.26803326704687

DNAH8 0.969953700438802 1.02666156531871 0.261101298012313 4.03687755567176

DNAJC5B 0.946500910708397 0.98908945559076 0.717912690864952 1.36269767007762

DOCK10 0.613995569237238 0.938722827712122 0.734210109310849 1.20020214389987

DOCK11 0.0284413332302058 1.25137017278604 1.02394082898294 1.52931425822132

DOCK2 0.843816964128619 0.978929052554984 0.792028623556915 1.20993365824655

DOCK4 0.719719705494887 1.04662348598486 0.816007262620056 1.34241540681623

DOCK8 0.812267201196708 0.974596554267759 0.788139707770551 1.20516506683497

DOK1 0.204990842112476 1.27129067238055 0.877088845230779 1.84266392449278

DOK2 0.77768134810043 0.975202533725877 0.819206026021522 1.16090452410855

DOK3 0.116898333481052 1.20267197140624 0.954905367149935 1.51472587814983

DOK5 0.572431429495662 1.07640126519877 0.833592079849863 1.38993605113209

DOK6 0.960787022316816 1.01360725609472 0.591395753915968 1.73724559705556

DPEP1 0.123815394361954 1.06960425260186 0.981760320670547 1.16530810330833

DPEP2 0.0900799288694379 1.25841256635698 0.964703712336163 1.6415425450476

DPP8 0.913444513215038 1.01624871214835 0.759936435378482 1.35901030252459

DPT 0.405887328276302 1.04992296367411 0.935977722758005 1.17773981457807

DSC1 0.814893266189216 1.28280015267391 0.15947241849706 10.3188767512831

DSG1 0.838807666576366 0.942722707276852 0.534036030664939 1.66416880469437

DSP 0.922655863480603 0.987518760156685 0.766356118960003 1.27250670743622

DUOXA1 0.932940377786464 1.01205420845354 0.765588163847827 1.33786514632181

DUSP16 0.229289774921525 0.838881360978846 0.629951984888376 1.11710408837336

DUSP4 0.910863491324103 1.00657819400466 0.897417158002845 1.12901748268387

DYSF 0.0130074426983784 1.28632523272753 1.0545181198624 1.56908883136825

E2F5 0.377267455743349 0.892429113929483 0.693197213970812 1.14892227974606

EBF1 0.185883739602774 1.19471327552408 0.917885449869898 1.55503043535093

EBF2 0.0721187748922566 1.43244612114444 0.968222634961673 2.11924594188297

EBI3 0.789535810674018 0.971424269967096 0.785148831437363 1.20189329015946

ECM2 0.244952019846799 1.11644880226679 0.927249844286954 1.3442525073072

ECSCR 0.0583197063016526 1.24342349518579 0.992356567104361 1.55801053737315

EDA 0.321667235940352 0.918455630680492 0.776244893335416 1.08671986478911

EDA2R 0.382397121794386 1.09856362652147 0.889651927387286 1.35653282409025

EDAR 0.000117515911255426 1.25362630634822 1.11740025166176 1.40646014141405

EDNRA 0.256518225954469 1.09792016690272 0.934308867315678 1.29018222459448

EDNRB 0.478723934453348 0.92679354109446 0.750978791735788 1.14376900821536

EFEMP2 0.0180337879181389 1.23990430215502 1.03750787202158 1.48178410975039

EFTUD2 0.416281009112844 1.18635585107486 0.785732730596167 1.79124548408678

EGF 0.190844071671553 0.881153495840416 0.72898716107509 1.06508252091398

EGFR 0.385699187956614 0.894024761520766 0.694105042478361 1.15152639052782

EHD2 0.0178834604373279 1.20526512890486 1.03269523976493 1.40667253514669

ADGRL4 0.333085098462582 1.10032340345964 0.906660470636728 1.3353527934782

EMCN 0.508495234695345 1.08110744483506 0.8579665441909 1.36228308107284

EMILIN1 0.0505730384596834 1.15406934456975 0.999642467966939 1.33235241074181

EMILIN2 0.877960452632116 1.02176635643529 0.776226906193613 1.34497590693234

ADGRE2 0.892207201146918 1.01660099177407 0.801178824143455 1.28994619594562

ADGRE4P 0.510162191972786 0.852577216071192 0.530390518997183 1.37047681534361

ENG 0.0512786976444577 1.22387475311269 0.998878768165289 1.49955075535132

ENO3 0.000168107796306109 1.75621476644274 1.30971831811648 2.35492644731966

ENOX1 0.415190077684323 1.12240099382466 0.85019467709268 1.48175944272731

ENPEP 0.112802512165906 1.24395662332112 0.949784832885176 1.62924067338898

ENPP2 0.203343383674274 1.11667914514115 0.942068919539716 1.32365295927864

ENPP3 0.934959099959499 0.996004816817593 0.904703592677042 1.09652001291209

ENPP4 0.646852112621146 0.941877815547843 0.729022469323782 1.21688131264878

ENTPD1 0.925665058446673 0.987365849409905 0.755925885230511 1.2896652166947

EOMES 0.989416746161696 1.00194770415176 0.751588281821452 1.33570363739848

EP300 0.565539454270309 1.08357908178629 0.824057866440054 1.42483152494758

EPCAM 0.265399861890502 0.85161917307191 0.641931556651558 1.12980146937589

EPO 0.764505489638093 1.15036784195344 0.460051357984536 2.87651834699096

EPOR 0.00753541892017607 1.43123890642393 1.10027968448977 1.86174918626395

EPS8 0.195028627690691 0.830764684117002 0.627608347242575 1.09968257020213

EPS8L1 0.0218130023463205 1.33332030567825 1.04273475839697 1.70488518121995

EPSTI1 0.274127880647912 0.913047565962228 0.775692425986 1.0747247617506

ERAP1 0.0162308136925295 0.762653552562074 0.611472539612466 0.951212693221154

ERAP2 0.779599109889628 0.98381117014257 0.877539760445165 1.10295221040047

ERN1 0.663850820027273 1.07529258036759 0.775083159756628 1.49178074486439

ERP27 0.818720142945321 1.01457640795222 0.89647816999528 1.14823240768779

ESAM 0.00681002188983661 1.36773189445744 1.09017156244102 1.71595976226662

ETS1 0.749254278740386 1.03167813414418 0.852097848288925 1.24910510525115

ETV3 0.367668514911929 1.17528537187163 0.827053384280996 1.67014092631549

ETV7 0.124059790263479 0.861390610042829 0.712228572705847 1.0417916543997

EVI2A 0.325747312021517 0.921211368582183 0.782124997896992 1.08503166103486

EVI2B 0.406600086717975 0.938208387660848 0.807008841428565 1.09073771375166

EVPL 0.0320597260372964 1.2282893100799 1.01777695271763 1.4823430863001

F13A1 0.173177541733566 1.08911172890906 0.963222179472835 1.23145457333267

F2R 0.702649221085662 0.961596580990461 0.786457248003938 1.17573839762987

F5 0.0872723438159844 1.12432144393145 0.983014100655147 1.28594158358625

FABP3 0.078208148689839 1.10607341210627 0.988685310087166 1.23739918099982

FCMR 0.327672612746617 1.10032889287924 0.908611571805463 1.3324986276579

FAM107A 0.0889247184887141 1.20687367951363 0.971791301143446 1.49882395179804

PCED1B 0.0819723196222117 1.2188517500595 0.975210861317209 1.52336243119414

DENND6B 0.00210400300433797 1.65358007906526 1.20008073193163 2.2784526116675

FAM13C 0.395468731119477 1.17916652245889 0.806276763088358 1.72451167061032

FAM155A 0.150127800206335 1.28684098992657 0.912760432014704 1.81423260175733

FAM168A 0.509007124826586 1.11313175502841 0.809842147354429 1.53000471524034

FAM171B 0.0141079401429919 1.37036844357555 1.06554137856173 1.7623995735224

FAM177B 0.117897275040482 0.859396025186989 0.710735907448066 1.03915043600237

TVP23A 0.903089448505388 1.04480588912294 0.515963871426956 2.11568950152844

CCSER1 0.360419142996879 0.85442525775738 0.609885174812383 1.19701634216367

CALHM5 0.460290329002037 1.09786080723035 0.856873099970559 1.40662409882384

CALHM6 0.298222885639487 0.927318303763468 0.804421880684909 1.0689903608323

PIEZO2 0.486820595680072 1.12649026609037 0.805248394256876 1.57588680542161

STRIP2 0.505626453172227 0.924291172736776 0.733055736665409 1.16541502817413

FAM49A 0.787655799955671 1.03020549363813 0.829628370093149 1.2792756339842

NXPE4 0.0491720891083606 0.922772740757331 0.851757715747913 0.999708620587137

MINDY2 0.442891321246989 1.09799974503643 0.864752864055269 1.39415952257893

RIPOR2 0.965631623395298 1.0056052230331 0.77983909007265 1.29673143788835

FAM78A 0.982340013703376 1.00275872179367 0.785701119275017 1.27978060545603

FAM83A 0.133429133054574 1.15660141448829 0.956455005646111 1.39863017507305

FAM92B 0.709955305246709 0.904370621100765 0.532471293365109 1.53601936949748

FAP 0.135552462418375 1.11366875702309 0.966847684004753 1.28278540755469

FAS 0.00544617708482746 0.763194017478883 0.630770858486307 0.923417910766087

FASLG 0.293014789118962 0.866366096155931 0.663104944804868 1.1319327633567

FAT4 0.605211354364113 0.936982645973087 0.732063110015634 1.19926337885811

FBLN2 0.0529760150897307 1.12020361918125 0.998543545940132 1.25668645451543

FBLN5 0.102700359251369 1.16066908630893 0.970491166733536 1.38811436321201

FBN1 0.155683795717292 1.10584932020914 0.962439041910897 1.27062875231971

FBP1 0.702310833733312 0.960296076965705 0.78014895858349 1.18204164126566

FBXL7 0.108882890258486 1.19193184885754 0.961686660119785 1.47730190220587

FBXO6 0.0980380139255652 0.846831740503436 0.695441570676297 1.03117792631622

FCAR 0.394340704665197 1.1176643881436 0.86525628335249 1.44370368474461

FCER1A 0.23683141044316 0.8757500211457 0.702930759585325 1.0910578162622

FCER1G 0.928941617346934 1.00636031839076 0.87545876050559 1.15683472040039

FCER2 0.0617350228113251 1.257796368052 0.98879648994396 1.59997706259502

FCGBP 0.0693954967750798 0.937490048287151 0.874396181440714 1.00513658372722

FCGR1A 0.3252592988765 1.108390571653 0.902912590561896 1.36062966910754

FCGR1B 0.542658763428489 1.19152658826958 0.67778997954882 2.09465417517447

FCGR1CP 0.192817572023817 1.26767657953068 0.887113588588122 1.81149734483069

FCGR2A 0.644889517145095 1.03730377021669 0.88769222137508 1.21213083295806

FCGR2B 0.330439900656313 1.11266368912184 0.89741348701376 1.37954298994309

FCGR2C 0.644889517145095 1.03730377021669 0.88769222137508 1.21213083295806

FCGR3A 0.808020916896258 1.01454072339064 0.903020407505282 1.13983346429742

FCGR3B 0.987312299976467 0.998829506015324 0.86457340687638 1.15393369047895

FCGRT 0.573985704810272 1.05344581776564 0.878576167106703 1.26312109583225

FCN1 0.149300074257145 1.1408433312839 0.953772844953579 1.36460532863912

FCRL1 0.101622924829422 1.30581938513478 0.948725258760153 1.79732145934661

FCRL2 0.429198239041999 1.16812575707955 0.794638603131548 1.71715516836875

FCRL3 0.642980089105787 1.09266533940502 0.751194466432862 1.5893588109169

FCRL4 0.909874626993849 1.06233745567239 0.372851495239296 3.02683745173175

FCRL5 0.323089575487979 0.862549854164985 0.643296954076771 1.15653003827413

FCRL6 0.599908430678697 0.902845972982544 0.616254272499293 1.3227183766612

FCRLA 0.845016148787859 1.02848889254772 0.776036425702427 1.36306668999019

FERMT2 0.217816342143632 1.11284691469518 0.938822627080335 1.31912910897556

FERMT3 0.358658724213614 1.08082290999966 0.915548527317344 1.27593254527209

FGD2 0.859087112755078 1.02682060642761 0.766649540747188 1.37528362275776

FGD3 0.0684852141086473 1.27701799232203 0.981626212187644 1.66129931379873

FGD5 0.323436712431123 1.10121214521323 0.909410216380488 1.33346664346001

FGF14 0.778326849175652 1.11373699460568 0.526084166702713 2.35781681271212

FGF7 0.446954696559791 1.07130200248077 0.897063061103175 1.27938383630236

FGL2 0.0492189512012187 0.8681928140878 0.754123209124165 0.999516727921828

FGR 0.386520113129029 1.07461218327859 0.913097455705384 1.26469670595971

FHL5 0.616655985843707 0.928769278233104 0.695438106459237 1.24038697934106

FIBIN 0.489076234860598 1.05332326005338 0.909158093795407 1.22034869154358

FICD 0.439553244537234 1.17404969079397 0.781596244650438 1.76356102768878

VEGFD 0.096199563280335 1.52863863312402 0.927175864014952 2.52027275662733

FILIP1L 0.544284458349524 1.05702492002987 0.883551371890507 1.26455768969437

FKBP11 0.54950378025499 0.923451492170323 0.711464267220934 1.19860223159568

FKBP7 0.738662153910648 0.957618306091191 0.742510960852569 1.23504280543953

FLI1 0.554211501598279 1.07600395682378 0.844092690702837 1.3716319639451

ANKRD36BP2 0.393643119325987 0.855204287704666 0.597021660496816 1.22503825589816

FLT1 0.00644945197074702 1.36900166471553 1.09209238596182 1.71612363760168

FLT3 0.560891301121131 0.843264673084554 0.474712550785222 1.49794924885844

FLT3LG 0.0629285328124408 1.45372752320318 0.980030342970148 2.15638600057389

FLT4 0.00134290778518917 1.45890640358267 1.15816593608132 1.83774002334764

FLVCR2 0.873649335550452 1.01869171501428 0.810805015496677 1.27987961396988

FMNL1 0.223823428896668 1.14256366248814 0.921765746513205 1.4162510678841

FMNL3 0.248222821074595 1.15200640914271 0.906025712168509 1.46476943080291

FMOD 0.510482979364985 1.05043754239491 0.907243129387424 1.21623299723162

FN1 0.0267415340695828 1.12156631198391 1.01330979475743 1.24138836778768

FNBP1 0.0874597225080597 1.20497758095417 0.972984207574083 1.49228626662126

FNDC1 0.131825003353604 1.08851732147199 0.974824642745286 1.21546984676931

FNIP2 0.253192424239078 0.863923756707634 0.672219428294648 1.1102985513187

FOLR2 0.141196153172924 1.10446810231556 0.967541282349388 1.26077285929391

FOXP3 0.188029884396606 0.86957952408354 0.706236351472603 1.07070182259613

FPR1 0.578336334548705 1.03763624757771 0.910889112602097 1.18201981711191

FPR2 0.872939371315123 1.01568142913355 0.839346353078818 1.22906206919553

FPR3 0.879957077354867 0.989728354240348 0.865612329408754 1.13164078410989

FRZB 0.672117611829943 0.969792652126561 0.841378167122322 1.11780626699093

FSCN1 0.0405145816310321 1.13852193761974 1.00562049750471 1.28898745168559

FSTL1 0.307214383206278 1.09191900961744 0.922325454329035 1.29269675684195

FSTL3 0.000918314774595242 1.29508252464971 1.11145296864616 1.50905057880775

FUCA1 0.0429078249893944 0.752997209126697 0.572163969528201 0.990983052323514

FUT7 0.30034179429369 1.18427735207407 0.859903828593563 1.63101128288901

FYB1 0.207050467349576 0.892954894369453 0.748941043466831 1.0646611643653

FYN 0.00821548373262447 1.31174928538843 1.07265284768331 1.60414079115471

FZD4 0.156507542728494 1.19367208928176 0.934400333280232 1.52488500483332

GAB3 0.989733485629442 0.997868923720352 0.72102430592276 1.38101084907599

GALM 0.0813586875815033 0.784668719466677 0.597384245491775 1.03066829089645

GALNT15 0.0692691637006071 1.20538972915136 0.985372439807748 1.47453322261284

GAPT 0.417998822890619 0.872684296003581 0.627669019362094 1.21334311077718

GAS7 0.212778561966073 1.12227242003386 0.936032651636411 1.34556778822486

GATA1 0.187454908205969 0.635596275397837 0.323988579139338 1.24690390745491

GATA2 0.0974394881300411 1.19892369874391 0.967422309211234 1.48582270816325

GATA3 0.767655173688343 1.0413212996577 0.79602729975938 1.3622020871

GATM 0.915109239993383 1.00861699220235 0.861414045101108 1.18097475046383

GBGT1 0.0446510125965266 1.24642960717125 1.00527577202133 1.54543341128102

GBP1 0.141086254148381 0.88339066298907 0.748934124190935 1.04198625519875

GBP2 0.0377426220503667 0.814731806105336 0.671536101852209 0.988461996382361

GBP4 0.0890097152452648 0.887744628618665 0.773910821213337 1.01832214260258

GBP5 0.188209710690326 0.896114876359897 0.761047145890301 1.05515390993827

GCSAM 0.473017871581234 1.1104015096048 0.834175760996847 1.47809558870326

GDF5 0.373340782064745 1.21337907806171 0.792624070227322 1.85748684954223

GFI1 0.0807793589758293 0.839913749480093 0.690547505178485 1.02158808955999

GFRA3 0.0256575207805484 1.27761458505558 1.03024630441941 1.58437746483022

GGT1 0.365846435253864 1.06454245857481 0.92958512338982 1.21909292392288

GGT5 0.0228480072129999 1.2144827166813 1.02734702858208 1.43570597673632

GGTA1P 0.594075772414082 0.930621299743622 0.714382699371902 1.21231379804964

GHR 0.807260579853425 1.02947360600588 0.815203005649553 1.30006378548406

GHRL 0.454995251380891 0.82364976365472 0.495111587270169 1.37019401405827

GIMAP1 0.564454520331832 1.08285390314377 0.826013185966247 1.41955672799831

GIMAP2 0.616136631500874 0.949005498749538 0.773393902711586 1.16449254836279

GIMAP4 0.845987949607452 0.982608527971814 0.823183851592123 1.17290872187971

GIMAP5 0.523651461055322 0.850468065738675 0.51697768878394 1.39908538904775

GIMAP6 0.74268677662861 0.968326655084902 0.799045202968197 1.17347117217502

GIMAP7 0.727581486435776 0.966898091326707 0.800064631324461 1.16852049497997

GIMAP8 0.701263991250099 1.04235958450469 0.843261463322639 1.28846573769384

GIPC3 0.0237901488933457 1.38720372594604 1.04447435815663 1.84239484890256

GIT2 0.608031379976531 0.896034002879704 0.589026927289456 1.36305641918825

GJA4 0.00902455360412094 1.30065665546116 1.06775041433839 1.58436626451102

GJA5 0.182699890775367 1.1300493505405 0.944052472001133 1.35269126720265

GJB2 0.0770156717722206 1.19665850590413 0.980725486267173 1.46013497130899

GJB3 0.135046038082536 1.12680756081079 0.963498721330718 1.31779653775435

GJB5 0.270753423577154 1.05374234080656 0.96000930091315 1.15662725324881

GJD3 0.106954320826118 1.3294606736483 0.940387511210087 1.87950782173089

GLIPR2 0.716885589703938 0.964315040509734 0.792361178560398 1.17358538317436

GLIS3 0.130765535631065 1.20930279152238 0.945110746377754 1.54734590331205

GLRX 0.206363013460848 0.860881117801529 0.682395605982613 1.08605080761041

COLGALT2 0.0947740599029107 1.1534897224508 0.975583616820677 1.36383854429178

GLT8D2 0.817601034589518 1.02193288362877 0.849862598364189 1.2288419571022

GMFG 0.809007146511162 1.02583902929744 0.834141273272671 1.26159170844182

GMIP 0.636709398950158 1.08857665174467 0.765423707248705 1.54816099305713

GMPR 0.0066119990486715 1.21464092448898 1.05560705784615 1.39763424702156

GNA15 0.551635232505901 1.05284441112116 0.888656274154195 1.24736794896776

GNAI2 0.0036531220085292 1.48075629079568 1.13638697091238 1.92948286882465

GNG11 0.550243621649265 1.06442448496201 0.867271487786662 1.30639540229569

GNG2 0.792736165574937 0.96865938364663 0.763869184699829 1.22835299593265

GNG7 0.177531512393934 1.18159878171854 0.927109312274995 1.50594505143382

GNGT2 0.405518120365155 0.875798563051058 0.640756215451468 1.19705920059139

GNLY 0.247561717920783 0.917187176126051 0.792177768371428 1.06192366112407

GNS 0.825539802601608 1.03438982210169 0.765801028122574 1.39718055314013

GPBAR1 0.0293476074987544 1.31395918299992 1.02780211822621 1.6797870951749

GPC5 0.846065360654834 1.05652774058029 0.606442678843181 1.84065354494013

GPC6 0.566668412626223 1.05286377124069 0.882760256613611 1.25574538781754

GPIHBP1 0.0128898197849856 1.35985502965676 1.06728588832084 1.73262452161917

ADGRG5 0.200378351766351 0.853878115159108 0.670481308845936 1.08743946464763

ADGRF4 0.204951347210823 0.896869930711933 0.757945591503706 1.06125780218526

ADGRF5 0.222866209745844 1.13283316981017 0.926982015016444 1.38439685973777

ADGRA2 0.0400343126714542 1.20371598326902 1.00847211142598 1.43675977943357

GPR132 0.0294580526013282 1.32083953699148 1.02816598271603 1.69682435696934

ADGRD1 0.0597694301880608 1.36593920669358 0.987248225295643 1.88988935971392

GPR137B 0.234614962141646 1.18286709666716 0.896731187996919 1.56030545954715

GPR141 0.0441342030990945 0.64377539925054 0.419267086894114 0.988502979688602

GPR15 0.0615033439874578 0.847085805361848 0.711839006765422 1.00802899929027

GPR157 0.950016603567909 0.992003909051427 0.77179044770327 1.27505044730957

GPR171 0.091116825621524 0.812320667675287 0.638272191105088 1.03382988688564

GPR174 0.467113585404055 0.899257162403752 0.675441990497784 1.19723596624261

GPR18 0.0999316591662171 0.748632748450342 0.530250467635446 1.05695520562488

GPR183 0.533976941955155 0.952458781180071 0.81692726463261 1.11047552104287

GPR25 0.291455079940808 0.86072447005603 0.651400716986681 1.13731316842929

GPR34 0.12622729439918 0.877088927090532 0.74137969538859 1.03763967479795

GPR35 0.224754099938799 1.18461288482936 0.901131147069604 1.55727353500894

GPR4 0.00939935053713213 1.37861361134882 1.08196196500347 1.75660101821615

GPR55 0.548383835610191 1.08533863708891 0.830659602569447 1.41810189578774

GPR65 0.443271673514732 0.905853028751365 0.703523935641636 1.1663707062783

GPR78 0.142018552647665 1.77949665845111 0.824500404549849 3.84063893718466

GPR82 0.0163884460052768 0.534999646677258 0.321015942952043 0.891621205204596

GPR84 0.290796121243182 1.11720439114623 0.909555179134797 1.37225940792697

GPRIN3 0.0021842761310408 0.72294439075233 0.587457791062078 0.889678543841166

GPSM3 0.296927615194935 1.12759831323766 0.899812686829229 1.41304737600095

GRAP 0.00245867293246082 1.5421353241026 1.16511797670411 2.04115068636434

GRAP2 0.814916151456911 0.961152776834768 0.689791494800876 1.33926652819033

GREM1 0.575088841943187 1.03221615041759 0.923896885341304 1.15323495304274

GRIN3A 0.23883717213976 0.643752613828481 0.309340628715836 1.33967991702665

GSDMA 0.584923342384771 1.04526174798234 0.891742592152787 1.22521020237182

GTF2A1 0.394954151817198 0.900542436752765 0.707425013125598 1.14637829500754

GUCY1A2 0.797783853889642 1.04178364315585 0.761703005332538 1.42485082971842

GVINP1 0.48090841252888 0.88756249101941 0.637022925283272 1.23663865804245

GYPC 0.0964512251007556 1.17756549233332 0.971156524959676 1.42784448551359

GZMA 0.0213890262004685 0.848701536329744 0.738024134448419 0.97597661668178

GZMB 0.345745123993002 0.945022261492505 0.840220208938933 1.06289644692575

GZMH 0.515053928963759 0.950724864180545 0.816551316025583 1.10694545417008

GZMK 0.664771612534112 1.03893349626045 0.874101178540329 1.23484882088185

GZMM 0.919577572934508 0.990103045289102 0.816258294803333 1.20097283731364

HAMP 0.0411391907662265 1.43294437801695 1.01457824965999 2.02382575338916

HAPLN3 0.0801157776727174 1.15146042888278 0.983222830903332 1.34848487810722

HAVCR1 0.811971445938253 1.01386512874378 0.905124631786149 1.13566956768613

HAVCR2 0.877161457958178 1.01316407435115 0.858339058216839 1.19591603309807

HCK 0.961358767016037 0.996050115957791 0.848686964508261 1.16900090962794

HCLS1 0.938662039508399 0.9928764744744 0.827591890930139 1.19117128184613

HCP5 0.98006392888847 1.00221851102502 0.842318126111969 1.19247338114114

HCST 0.391655620417762 1.07408079963241 0.912060545828962 1.26488265435322

HDC 0.151219396745714 0.790631149474442 0.573649816330196 1.08968502512248

HECW2 0.176120096329043 1.21613355258393 0.915922077602609 1.6147452429486

HEPH 0.576703489552525 0.94523955935803 0.775648601446022 1.15191057253204

HEPHL1 0.231528492432319 1.11373206040695 0.933567393400782 1.32866583724588

HERC6 0.794546031230122 0.970007775130004 0.771329994693939 1.21986061774508

HERPUD1 0.229309254259725 0.81632194869895 0.586355230403633 1.13648090675158

HEYL 0.000102195215084216 1.50743071554602 1.22553259333225 1.85417130032668

HFE 0.428604168272552 0.874093959681998 0.62640554821853 1.21972139698547

HGF 0.414694254963621 0.879986177583566 0.647226044366634 1.196453201286

HIC1 0.329800351451436 1.12673159738557 0.886335658183443 1.4323288032312

HIPK3 0.784634721333887 1.03664431633311 0.80081980912338 1.34191415639691

HIST1H2AE 0.741031973529859 1.02336845700969 0.892353174857977 1.17361940127469

HIST1H2AG 0.662303716050194 0.956644759959296 0.784087091953527 1.16717799100284

HIST1H2AM 0.342794790164561 0.908140260974961 0.74420954024076 1.10818081334575

HIST1H3H 0.98327342562598 1.00185608706603 0.842396852142242 1.19149972680783

HK3 0.343156012987886 1.07649584059115 0.924323609213754 1.25372032398456

HLA-A 0.678243699128888 0.957253731693985 0.778738382290321 1.17669133521717

HLA-B 0.638149698015839 0.955846873161689 0.791871920498471 1.15377654047621

HLA-C 0.726266939314148 0.96472743371378 0.789034523774258 1.17954157051086

HLA-DMA 0.894204997132759 1.01044210112911 0.86700386211529 1.17761094771045

HLA-DMB 0.694408941914781 0.971121228397854 0.839042966541529 1.12399063915909

HLA-DOA 0.730813897067291 0.976400162723732 0.852195337857859 1.1187074552219

HLA-DOB 0.493267904742656 1.07598164880971 0.87260729328873 1.32675548036267

HLA-DPA1 0.572051624045378 0.965603832591272 0.855205034154739 1.0902540610469

HLA-DPB1 0.988689683313323 1.00096368054661 0.876155406212941 1.14355088454468

HLA-DPB2 0.307381410971279 1.1128137316468 0.906325323810805 1.36634646391077

HLA-DQA1 0.663194407386807 0.970984208269615 0.850475032085272 1.10856909037918

HLA-DQA2 0.898860864418324 0.993504431668005 0.89851762584217 1.09853276925849

HLA-DQB1 0.943321890898938 1.00427975204574 0.892737834501924 1.12975812314682

HLA-DQB2 0.921736593450301 1.00649052989199 0.884622190994494 1.1451478349457

HLA-DRA 0.289317365868306 0.937082257646662 0.830950921276786 1.05676898010643

HLA-DRB1 0.84829038964241 0.987784501329844 0.870911295625913 1.12034167654952

HLA-DRB5 0.621696738651254 0.973658730463612 0.875706562242219 1.08256733965846

HLA-DRB6 0.919151012112643 0.993739071878105 0.880244742852668 1.12186678874882

HLA-E 0.64241451151854 1.06598786068044 0.813967823490872 1.39603813114463

HLA-F 0.74007619522065 1.02745937779237 0.875507945426647 1.20578320108682

HLA-G 0.238348071874637 1.10140896649956 0.938038976640911 1.29323166914633

HLX 0.0259590192207107 1.32943956445155 1.03471397871988 1.70811411837279

HMCN1 0.140535947616014 1.18239001667403 0.946233262157632 1.47748573997763

ARHGAP45 0.082835731402024 1.27399417272518 0.969007329688372 1.67497303932642

HMSD 0.220523021145011 1.26765252834856 0.867404217379686 1.8525883324419

HNMT 0.593357285432294 0.937745425411485 0.740666581533508 1.18726361470162

HPGD 0.241987736383552 0.918440978020915 0.796447369000797 1.05912061856177

HPGDS 0.213717437880299 0.821229885111458 0.602056053514162 1.12019224831914

HRH2 0.910006100828657 0.981435671467222 0.70916201968935 1.35824529583558

HS3ST1 0.46894731240986 1.11321146347451 0.832758990827562 1.48811333898606

HS3ST2 0.232099766518481 1.14039785608598 0.919334432995402 1.41461825369486

HSD11B1 0.661933081500914 1.03396540006822 0.890189599459598 1.20096263670935

HSD17B14 0.0181831307226761 1.27429548833044 1.04210897845972 1.55821418406683

HSH2D 0.607024908369964 1.08846751328412 0.787990290696597 1.50352300207603

HSP90AA1 0.765719185104843 1.0408078710975 0.800046530361895 1.35402252672541

HSP90AB1 0.101712435609841 1.35489589370315 0.941756045724325 1.94927645127221

HSPA12B 0.0695425122827726 1.22285779296796 0.984045076724324 1.51962670937826

HSPA1A 0.00144713049456968 1.2432433280822 1.08735238258427 1.42148396194012

HSPA1B 0.0258447449166154 1.26070330408274 1.02831315452836 1.54561167862733

HSPA1L 0.0648996226374053 1.53853957342821 0.973740491518045 2.43093928990708

HSPA2 0.941119240767539 1.0058457995867 0.861708530038271 1.17409279040242

HSPA4 0.926637041672429 0.983244876466174 0.6862100156769 1.40885510996707

HSPA5 0.334934282321238 1.15916037531247 0.858538441528181 1.56504672441093

HSPA6 0.47493073989573 1.05634641483082 0.908867595580289 1.22775611491954

HSPA8 0.357875307098427 0.8768106940799 0.662515605992997 1.16042095657593

HTR2A 0.715749566496329 0.908732097379502 0.542903162728046 1.521070572988

HTRA3 0.0313598512914981 1.15947989153286 1.01332218590756 1.32671882404802

HTRA4 0.640030386244798 1.08258723264783 0.776296586578734 1.50972596885588

HVCN1 0.37220438332987 1.13563845136122 0.858836725025419 1.50165293894709

HYDIN 0.357405220689298 2.65452667548491 0.331926209289573 21.2291517622025

ICAM1 0.285043138472735 1.10215219513495 0.922141982432383 1.3173019821054

ICAM2 0.017381091628525 1.32278968142942 1.05046167002054 1.66571764704364

ICAM3 0.668277861967226 0.946977524917528 0.738108611583143 1.21495186294533

ICK 0.734149651015195 0.950443159637435 0.708815817538373 1.27443854574068

ICOS 0.0573621878861609 0.781927594596901 0.606726235470028 1.00772099086574

ICOSLG 0.874422096311438 1.03394350751738 0.68346514857799 1.56414585141843

IDO1 0.318155574580873 0.942648028320687 0.839436338896372 1.05854996278232

IDO2 0.522925735552455 0.632149946041346 0.154787964325892 2.58168363425677

IFFO1 0.1021624170314 1.24070647090349 0.957956298776629 1.606913122141

IFI27 0.935216432224961 0.993124699379397 0.840924487184998 1.17287186132378

IFI30 0.140507784719275 1.26633691685612 0.925084635374134 1.73347294471506

IFI35 0.21345279550923 1.16581500187645 0.915510050935399 1.48455455755132

IFI44 0.529161585544647 1.04831792108603 0.905035992923595 1.21428371055174

IFI44L 0.341475783162974 1.07309702514438 0.927919631411547 1.24098810542676

IFI6 0.250165687329763 1.07621676560265 0.949576532277858 1.21974636818985

IFIH1 0.802976344625453 0.97156097773678 0.774531579572095 1.21871174572681

IFIT2 0.918990466696292 1.00915966017796 0.846543102580038 1.2030140185735

IFIT3 0.533937392678417 0.952788853443622 0.818115223878218 1.10963171537503

IFIT5 0.0795682752401377 0.819041323497529 0.655219993991519 1.02382206853912

IFITM1 0.261768199009417 0.909012411632551 0.769505716264903 1.07381082042225

IFITM3 0.562816609721916 1.06084441073984 0.868496291297519 1.29579236558011

IFNAR1 0.618470458481121 0.919535323924608 0.660984469033519 1.27922099770586

IFNAR2 0.43425229526865 1.16567431268791 0.793775781473464 1.7118141356469

IFNE 0.0454648786817523 1.39521975731226 1.00674315582397 1.93359960773833

IFNG 0.164137056597888 0.819427663951454 0.61897031584066 1.08480435856926

IFNGR1 0.0183679446343831 0.731591840769711 0.564228480899783 0.948599086361754

IFNGR2 0.553805469206001 1.12205733596496 0.766384248953658 1.64279559099983

IGDCC4 0.33712469278698 1.14625485152502 0.867428290572701 1.51470755441603

IGF1 0.143693155197835 1.33738810814183 0.905766859807707 1.974688003245

JCHAIN 0.144219490992141 0.953485089079077 0.894435294009889 1.0164332972823

IGLL1 0.457439511657427 0.884348639291569 0.639491188184486 1.22296058220466

IGSF10 0.0946498202891996 1.44095568286344 0.938869559710966 2.21154606462653

IGSF21 0.0587075202168355 1.35074795558134 0.988993475924357 1.84482515195754

IGSF6 0.196413259720885 0.890010220381497 0.745791745187682 1.06211713590928

IKZF1 0.485833704087147 0.925540304190326 0.744547351791241 1.15053106108005

IKZF3 0.623097875174693 0.947056613447876 0.762365167269764 1.17649161790466

IL10 0.9282153060376 0.983831711522675 0.69009618135817 1.40259410607471

IL10RA 0.983969668763598 0.998165254997254 0.83445561352081 1.1939926583751

IL10RB 0.402198416606772 1.16056853490853 0.819145415862015 1.64429818947632

IL11 0.966997919945805 0.997007201041437 0.865035150426466 1.14911325677162

IL11RA 0.00105385932010466 1.6324262450844 1.21755737819584 2.18865697285581

IL12A 0.335042529511608 0.85473386393959 0.621202762923094 1.17605719383374

IL12B 0.765697632223432 0.855888059778136 0.307560172339221 2.38179204186044

IL12RB1 0.777384199532284 0.968702699734939 0.777068077524666 1.20759679571829

IL12RB2 0.139389398110242 1.45329518566415 0.885253627957785 2.38583252298778

IL13 0.0634038963409734 0.320455810220838 0.0963717741447613 1.06558094645055

IL13RA1 0.352992586705267 0.875846382311418 0.662123171245077 1.15855617009371

IL15 0.00254731828376655 0.593257221359089 0.422634557487037 0.832762310747632

IL15RA 0.501059895794287 0.905630728613696 0.678496942860908 1.20879987041815

IL16 0.862789721081766 1.02399723905035 0.782532290303229 1.33997070609882

IL17A 0.481289337105197 0.890072908294122 0.643680640384416 1.23078081330211

IL17B 0.621252284652148 1.11277424773782 0.728305661600859 1.70020170337092

IL17RA 0.389781100986064 1.16680722429456 0.820935106845193 1.65840038672229

IL17RB 0.16037070842125 0.872849790008545 0.721907718753861 1.0553520015454

IL18 0.0508452079592046 0.818962628537779 0.670207453651129 1.00073459835115

IL18BP 0.638901368472089 1.05510015723904 0.84333002196097 1.32004827625757

IL18R1 0.190631410872143 0.834323910566012 0.636097137026615 1.0943240383002

IL18RAP 0.48937248463427 0.892238348059602 0.64578278059764 1.23275084698199

IL19 0.398692240037686 1.31926501143759 0.693233537708705 2.5106404634664

IL1A 0.0510925259157812 0.845362673771106 0.714068258048125 1.00079795194771

IL1B 0.989933131691387 1.00078897772794 0.885394020681983 1.13122356210431

IL36G 0.619827100599433 1.08178413529726 0.792973299381318 1.47578350531333

IL1R1 0.632095903058416 1.04326224439643 0.877195855250091 1.24076750256963

IL1R2 0.378600677370121 0.940989020623403 0.821832189000341 1.07742231173842

IL1RAP 0.467388494990994 1.12351453689625 0.820674050091547 1.53810750379689

IL1RL1 0.960565269558033 0.990439440368505 0.676779835190573 1.44946736594368

IL1RN 0.75452975276844 1.02001662278422 0.900851687637529 1.15494473178448

IL20RA 0.253346863238066 0.87371960818793 0.693065527611683 1.10146288239542

IL20RB 2.36332349254861e-06 1.59524923901422 1.3140054950629 1.93668911137515

IL21R 0.727375146168532 0.954474056326907 0.734507995706422 1.24031423691304

IL22 0.283176949972888 0.7398767488038 0.426785779269584 1.28265192986831

IL22RA1 0.711101076982441 0.9654408739709 0.80148809658143 1.16293190767182

IL22RA2 0.424250118109255 0.801911799699039 0.466624543628669 1.37811553909236

IL23A 0.0528398671988142 1.16655445620401 0.998113191202767 1.36342181556538

IL23R 0.148601815748291 0.73712747949926 0.487322872345771 1.11498341626658

IL24 0.920800849979726 1.00768055152371 0.866600690329758 1.17172776949063

IL27 0.704703036782914 1.09238763991065 0.691678167246898 1.72523987648667

IFNLR1 0.771416263782422 1.03865707802148 0.804165317718952 1.34152580564429

IL2RA 0.474561285187402 0.933996714740283 0.774576927702897 1.12622753395548

IL2RB 0.0988382421600443 0.86444396717569 0.727130877947526 1.02768758011728

IL2RG 0.377192623143799 1.0778016087498 0.912683388762972 1.27279221044893

IL32 0.264816832350069 0.893927960273607 0.734019113887872 1.08867355500636

IL3RA 0.21336289565549 1.1569136880668 0.919597813246468 1.45547244932126

IL4I1 0.327421661734498 1.07698663375855 0.928423598430495 1.24932219652258

IL4R 0.573010159578367 1.10676437035545 0.77777912143736 1.5749038997403

IL5 0.262741850282966 0.598744543620262 0.244004892961281 1.46921245784986

IL5RA 0.152015025426889 0.372411796879935 0.0963988911388595 1.43871516380372

IL6 0.162289746125243 1.09694831010969 0.963434893475733 1.24896410042971

IL6R 0.714514679429826 1.04226227304363 0.834940748212267 1.30106315703962

IL6ST 0.469166090451432 0.922303445183321 0.740902672040685 1.14811793383614

IL7 0.000128155785770527 0.64530419529985 0.515718567019924 0.807451061686324

IL7R 0.234636140659957 0.90860843695952 0.775774742759559 1.06418686534874

CXCL8 0.894710605411101 1.00648609275686 0.914589669663585 1.10761611301119

IL9R 0.657902928813532 0.88817758199032 0.525472527683779 1.50123817248329

INHBA 0.086444552242143 1.12620298544355 0.983124778254771 1.29010395473248

INHBB 2.68755469998512e-05 1.26886160434411 1.13536962034765 1.41804901428111

INHBC 0.894944125335046 0.929599585441787 0.314577801478506 2.74703232456975

INHBE 0.0451376223715874 1.52193949231925 1.00914107885299 2.29531813422335

INMT 0.661395694029771 1.05066117589588 0.842206404602687 1.31071065299685

INPP5D 0.0143477583555795 1.31808033602657 1.05664446065739 1.64420089907917

IPCEF1 0.611054593070876 0.882218973817481 0.544294820935844 1.42994253817327

IQGAP2 0.0117348812803427 0.813456582603414 0.692781418775267 0.95515207805462

IRF1 0.0638935204044002 0.809540372559345 0.647404979930791 1.0122807749696

IRF4 0.172001422770964 0.866442761037054 0.705333970823716 1.06435119986748

IRF7 0.0135036523913155 1.30712648988528 1.05688142798307 1.61662379082621

IRF8 0.185860854622381 0.882347891162442 0.732998550841597 1.06212734001305

IRF9 0.168292141252929 1.2043269758924 0.924433387790152 1.56896482106657

ISG15 0.0185091997017993 1.18862192918788 1.02942636958861 1.37243627352477

ISG20 0.151478991275798 1.17026309043477 0.944009092471378 1.45074418430506

ISLR 0.0479455591452818 1.13889358514612 1.00117686205882 1.29555391004509

ITGA11 0.0986856025752321 1.13086377615675 0.977270739470769 1.30859630660389

ITGA1 0.667562324207377 0.950706074269813 0.754853209495218 1.19737457333981

ITGA2B 0.00603432003781337 1.66679863069803 1.15746633569849 2.40025786462312

ITGA4 0.538902800687734 0.935836022403048 0.757419691101652 1.15627976287934

ITGA5 0.00788302961637767 1.21612424700174 1.05267423624994 1.40495333999454

ITGA8 0.611002855499856 0.922337807556675 0.675460357515452 1.25944775556868

ITGA9 0.0321669929301777 1.20508756393875 1.01600697861785 1.42935636006695

ITGAD 0.661054189425435 0.844014370255934 0.395479727994417 1.80125606136903

ITGAL 0.85125078026432 0.982805461013436 0.819859566697939 1.17813661440594

ITGAM 0.181806616429955 1.10631986320705 0.953823594614394 1.2831970677149

ITGAX 0.310167354535653 1.08601893381165 0.926033331086646 1.27364435491042

ITGB1 0.301919239580989 1.14199279190454 0.887532595130895 1.46940804643866

ITGB2 0.432588022177994 1.05507427868267 0.922855445024333 1.20623629577046

ITGB3 0.464874197248843 1.09737541520118 0.855325427619268 1.40792353764093

ITGB7 0.514557045414075 1.07279614963463 0.868452449759813 1.32522117818792

ITGBL1 0.0454604979200472 1.17137522014035 1.00320007500616 1.36774302608623

ITK 0.429492228365113 0.883594476836297 0.650008009583994 1.20112242923789

ITM2A 0.160843166113587 0.882773164584834 0.741584196072354 1.05084286347857

JAK2 0.07701265428557 0.820745740720437 0.659352778560056 1.02164363723749

JAK3 0.251882273282604 1.14557208118486 0.9079457025037 1.44538972933225

JAKMIP1 0.617619882463525 0.904905205100338 0.61126103426701 1.33961333098814

JAM2 0.345424667686981 1.10580385950884 0.897313556683906 1.36273676753933

JAM3 0.0497396319677622 1.23773712102496 1.00024267779636 1.53162149023503

JMY 0.854454279637758 0.975349220786929 0.747034254109216 1.27344375074746

JSRP1 0.651132319273494 1.03449449114649 0.893080445174321 1.19830062117591

JUP 0.0554478116787586 1.33381096740318 0.993313260998794 1.79102783242433

KCNA3 0.703219749039043 0.935334950930755 0.663141051181994 1.31925397903401

KCNAB2 0.0379829331941495 1.25070475857211 1.01248334508136 1.54497592549459

KCND2 0.475591077152785 1.15757598891243 0.774389062582955 1.73037331601392

KCNE4 0.00675928580851751 1.28630919700846 1.07205881180521 1.54337740811293

KCNH2 0.046944248138114 1.17196189815068 1.00214582821075 1.37055371788476

KCNJ10 0.0146863580092247 1.53775463291412 1.08835052431739 2.17272768121463

KCNJ8 0.00586132575393829 1.32306533498142 1.08417566994322 1.61459247717776

KCNK13 0.856421513535471 0.968502817382818 0.684757465589404 1.36982472541731

KCNK6 0.736336589349662 0.960382562436616 0.759019982022558 1.21516519733061

KCNMB1 0.134551195188208 1.12830097723488 0.963294686951441 1.32157180193537

KCNN3 0.857597314721817 0.963120145734267 0.638882730557299 1.45191029707447

KCNN4 0.195022997168327 1.10938815356074 0.948190607196583 1.29799015716862

KCNT2 0.770876347512754 1.1090940748116 0.552499693242126 2.22640787285129

KCTD12 0.282558334870686 0.928682469963301 0.811446224476627 1.06285679075457

KDR 0.336444327645586 1.11117947793841 0.89624745516942 1.377655049473

FAM30A 0.96368123134721 0.99156111593553 0.688489892737963 1.42804339904742

TESPA1 0.737278781387237 0.937634066478044 0.643627677165422 1.36594132572425

KIAA0754 0.330887028339464 1.11928751207784 0.891841951148475 1.40473828695778

JCAD 0.0832615710279983 1.15634200348929 0.981068710036124 1.36292882991282

KIAA1549 0.183067275180973 1.22259833316422 0.909472916321207 1.64353072799807

SHISAL1 0.685216328344558 1.05192857622115 0.82352895756274 1.34367312686313

KIAA1755 0.219114479656686 1.16516976814433 0.913070552289865 1.48687369797741

KIF21B 0.0777823409445637 1.23996020102629 0.976350316130073 1.57474348574322

KIR2DL4 0.00717000064963241 0.602895415987326 0.416922157786008 0.871824334184451

KIRREL1 0.0946960819291591 1.17620954206982 0.972338716769281 1.42282608210116

KIT 0.930553126790456 0.992390278852559 0.835742624674723 1.17839923019885

KITLG 0.0680760111529685 0.845790625584906 0.706521448493147 1.01251247765091

KL 0.156326187638011 1.19818507074731 0.933152056531237 1.53849252510721

KLHDC10 0.746001017332033 1.06745907380807 0.719122500479081 1.58452679967051

KLHL11 0.612377352958742 0.934140562445089 0.717728254337157 1.21580637954837

KLHL23 0.114701188020409 0.807764086205573 0.619560477654696 1.05313822055508

KLHL6 0.81825273733293 0.971309530273673 0.757753071557267 1.24505229871465

KLK7 0.343328620178736 1.04890584317099 0.950269200774378 1.15778083404332

KLRB1 0.07249004397167 0.836384534760387 0.688223421384752 1.01644185340108

KLRC1 0.0386985117477451 0.561631873573362 0.325028306672996 0.970470432690259

KLRC2 0.390929286582566 0.858101615301162 0.604932248416882 1.21722454722735

KLRC3 0.617840996422422 0.762885109089619 0.263452998953702 2.20909874619783

KLRC4 0.471720093314521 0.775518913724306 0.38807133500316 1.54979131746289

KLRD1 0.0939779671203553 0.70411920937349 0.467034594610764 1.06155703823599

KLRG1 0.999777266430617 1.00005337405044 0.687510035407366 1.45467949476125

KLRK1 0.802998764737071 0.924726997080534 0.500030373723852 1.71013615185271

KMO 0.62353563676347 0.888339519320109 0.553672608970637 1.42529554252833

KRT1 0.579843214420701 0.8760000278923 0.548214998343951 1.39977208063515

KRT14 0.624897923194722 1.04289412947665 0.881291528417996 1.2341298313048

KRT16 0.0188389332537104 1.13954856241754 1.02185664770741 1.27079559449097

KRT6A 0.0119340372024316 1.1259351531344 1.02648817694417 1.23501663003833

KRT6B 0.112120126361841 1.0739526797602 0.983467293545462 1.17276330990746

KRT6C 0.775385898914522 1.04676028511718 0.764746880943595 1.4327709230362

LAD1 0.132853433256749 1.29390891328304 0.924638528034176 1.810653812396

LAG3 0.605342240289221 1.05185032468693 0.868326160935804 1.27416304531426

LAIR1 0.682710576508173 1.03970234013721 0.862647440219988 1.25309704253123

LAIR2 0.345111835704034 0.910101892699659 0.748447839900083 1.10667091404269

LAMA2 0.0750007865413852 1.16933404467572 0.984352134345119 1.38907821736717

LAMA4 0.26198351638014 1.11941166939037 0.919158269469863 1.3632934905651

LAMC2 0.116872952963444 1.18169545389481 0.959129612994037 1.45590765506302

LAP3 0.548391693146869 0.920002791587983 0.700706911683098 1.20793033780217

LAPTM5 0.767307699892119 1.02241618536541 0.882786937530373 1.18413040752678

LAT 0.0376156955103738 1.89858716577649 1.03739511450666 3.47469655066328

LAT2 0.0460893929402018 1.28556636329898 1.00436831046121 1.64549285081172

LATS1 0.293967297202428 0.857597034306704 0.643699789132991 1.14257094016183

LATS2 0.0069459797280922 1.37604565277022 1.09138005266709 1.7349608267812

LAX1 0.311948609065766 0.884873918973494 0.698100629613298 1.12161745637336

LCK 0.700775148051662 1.03072556612671 0.88329924178178 1.20275795836095

LCN10 0.716927066701967 1.18902885374598 0.466358016906206 3.031554221839

LCOR 0.158766616896303 0.847175904832897 0.672665341465174 1.06696000148626

LCP1 0.959061154114007 0.996324095511449 0.86563174889789 1.14674826167195

LCP2 0.628291532367856 0.955891857143916 0.796334598403403 1.14741874130047

LDB2 0.250479533514195 1.15050628621357 0.905818770786739 1.4612908865504

LEP 0.0659232349864541 1.27519874814436 0.984129048351416 1.65235631444169

LEPR 0.911414732640121 1.01701054917236 0.755567049732843 1.36891948569433

LGALS2 0.414084414081087 0.953774721833675 0.851373841628318 1.06849209539849

LGALS9 0.260511656854857 0.884183459774093 0.713549606685094 1.09562164033692

LGI2 0.554350092256136 1.06656295033157 0.861416806079787 1.32056458498515

LGMN 0.0908194122234025 1.25749714701972 0.964226374876133 1.63996662605903

LHFPL6 0.0387973266644973 1.20759163671187 1.00975707567408 1.44418652385572

LHFPL2 0.334029255474212 0.87689775372129 0.67173645889895 1.14471927240906

LIF 0.0010723455986005 1.4345716834415 1.15560696852261 1.78087876846506

LIFR 0.784596323654699 0.955368357814271 0.68862760446803 1.32543147151039

LIG3 0.420891487798365 1.16758267222901 0.800634952990665 1.70271019444903

LILRA1 0.658172243184779 0.902984557755791 0.574576452978247 1.41909942065846

LILRA2 0.745255314177847 1.06239196634687 0.737432717844217 1.53054870342328

LILRA4 0.202091342546492 1.3262625303661 0.859431319206738 2.04667000159669

LILRA5 0.807715152419412 1.02281830104777 0.85288116898525 1.22661551808321

LILRA6 0.562319337201273 1.0854338154605 0.822557369511879 1.43232145430086

LILRB1 0.678648230357832 1.04746988333697 0.841120425959989 1.30444241113953

LILRB2 0.811344763486727 1.0226352540125 0.850943732126973 1.22896828928422

LILRB3 0.602696064996057 1.06978525161506 0.829821796790563 1.3791400623596

LILRB4 0.804959350895022 1.01947237049103 0.874777814609121 1.18810044886542

LILRB5 0.682874594075409 1.05780944522466 0.807819230957082 1.38516239713778

LILRP2 0.0520254198753889 0.32987036223436 0.107761138099877 1.00977456065631

LIMD2 0.221856180372382 1.145383716565 0.921232346122491 1.42407489673382

LIME1 0.017804287235129 1.29494096017682 1.04570043721202 1.6035874430869

LIMS1 0.716288038929766 0.947918167532883 0.71038685166604 1.26487258348261

LIPA 0.795476953213159 0.97141697729751 0.780143647844006 1.20958614018033

LMOD1 0.0628924636573961 1.11278540600987 0.994270082339742 1.24542755718303

LMTK2 0.635010422354416 1.07690700608392 0.793077330082434 1.46231477784404

UNQ6494 0.682216616943227 1.1205721322948 0.6497923605799 1.93243562074984

FAM83A-AS1 0.100184380064881 1.45134155109922 0.930903137493042 2.26274057214986

LINC00426 0.927523061710634 1.01481085598499 0.739272119447041 1.39304735879298

PCED1B-AS1 0.980218179001097 0.996923848792878 0.781444073436971 1.27182122697635

LINC00654 0.986886781280311 1.00142519026179 0.845008050492073 1.18679628094297

LINC00926 0.651993964412409 1.06931786941605 0.799120258166489 1.43087438238144

SMIM1 0.013365985172904 1.27062613079353 1.05101243941005 1.53612907299324

LGALS17A 0.214246391949721 0.813882659128496 0.588056351239251 1.12643113442129

LOC730101 0.852512138999759 0.980362097167352 0.795390590100941 1.20834952477926

LOXL2 0.0306241086141626 1.23142598024186 1.01963707533499 1.48720557686315

LOXL3 0.00639206087954516 1.51429599736778 1.12379441484542 2.04049098069195

LPAR4 0.751484127457535 0.875725056439847 0.385194100655462 1.99092969796681

LPL 0.0539191602754811 1.13508421980197 0.997867240177725 1.29116994141823

PLPPR4 0.414201302835854 1.13130163183579 0.841336821259211 1.52120215097541

LPXN 0.966651482047725 0.994831663292911 0.780281710836458 1.26837528618888

LRCH2 0.657853250845823 1.06974251728311 0.793786503445031 1.44163329600181

LRMP 0.402765563858618 0.899212158249229 0.701105903785823 1.15329581619134

LRP6 0.380025535093199 1.13854805542128 0.852189767205208 1.52113029795562

LRRC15 0.838543341432647 1.01490706006762 0.880260406355245 1.17014957521491

LRRC17 0.204591578165586 1.17453711774759 0.916056008893953 1.50595315960262

LRRC25 0.632912559446184 1.04426701930623 0.874214855427897 1.247397708744

LRRC32 0.102883407021876 1.17224103349175 0.968459184072912 1.41890237936795

NRROS 0.171400281575283 1.17277447371827 0.933316447509078 1.47366948249542

LSAMP 0.201966982062593 0.864145497532948 0.690526643134127 1.0814172752512

LSP1 0.458805367882295 1.06693565571847 0.898862677202518 1.26643559946915

LST1 0.673089281893534 0.961135158790614 0.799485949982537 1.15546845255209

LTA 0.534097779645246 0.893723550422592 0.62716608538122 1.27357298680214

LTB 0.802526595897185 1.02212471854801 0.861029755217519 1.21335985653936

LTBP2 0.260927512180725 1.0948469834642 0.934853547972906 1.28222214035539

LTBR 0.0816246592493716 1.43905543413279 0.955329294505174 2.16771384947401

LUM 0.654753283618619 1.02758707335999 0.912046413107106 1.15776475644398

LY86 0.445967886376364 1.07515336304902 0.892361381840014 1.29538859211062

LY9 0.815585536310249 0.946157079137559 0.594245225837456 1.50647103161916

LY96 0.635964537302334 1.03717953698129 0.891679016389072 1.2064222350875

LYL1 0.175201053044074 1.21152410811709 0.918026797340395 1.59885383389812

LYN 0.332012429565327 0.886248944911412 0.694376925692667 1.13113953429989

LYPD3 0.0276620515094956 1.17768012612413 1.01814210353488 1.36221699765924

LYPD5 0.0306978927523041 0.770074184928272 0.607602197407578 0.975990957279487

LYVE1 0.0951057014550594 1.15207184635226 0.975628663120894 1.36042491301122

LYZ 0.452142008927236 0.967768274752295 0.888544888464388 1.05405528271695

MAGEL2 0.02965288758725 1.66369918352928 1.05154824918996 2.63220919763615

MAN1A1 0.671037441952158 0.961845785863293 0.803790103462352 1.15098122233388

MAN1A2 0.2834969477057 0.866844229210153 0.667620614775566 1.12551784813827

MAN1C1 0.680844901476877 1.05514659271159 0.817002446815255 1.36270623968233

MAOB 0.0185351347902101 1.15466835399088 1.02440407935227 1.30149716755426

MAP1LC3C 0.0869831769206775 1.6316871441128 0.931397767173636 2.85850259695397

MAP3K2 0.5348878956798 0.917269462388984 0.698310210855741 1.20488466809086

MAP4K1 0.917816846032718 1.01163720364477 0.812044354419838 1.26028809415144

MAP7D1 0.019618766944479 1.40043007078006 1.05539741415132 1.85826150116363

MARCO 0.0453265493433378 1.10267460230767 1.00203570928212 1.21342110596584

MBNL3 0.924901699029365 1.01279386938774 0.777538810483527 1.31922858131225

SLC25A53 0.00452457404003939 2.20700261369427 1.27778208995192 3.81196494704087

MCOLN2 0.561160369484195 1.04529491536778 0.900225266252194 1.21374227213441

MED13 0.469292981674324 0.913422983718651 0.714745117522363 1.16732738249083

MED13L 0.991087081775768 0.998476941216274 0.764179802521659 1.30460946344149

MEF2B 0.0309261964408547 1.67565127591913 1.04851570241373 2.67788760056306

MEF2C 0.708368623442386 1.04501455504275 0.829704994840987 1.31619723521187

MEI1 0.612251811421986 0.922286152329155 0.674537640061342 1.26102932773442

MEOX2 0.0117609984454877 1.25806658576609 1.05228711469556 1.50408715655428

MET 0.477587341249794 1.0963784984856 0.85050774617107 1.41332729461085

MFAP3 0.467608755382453 0.909704946831372 0.704697516020819 1.17435221705113

MFAP4 0.554405524983828 1.03749015872747 0.91831322849829 1.17213364247901

MFNG 0.0356292417834302 1.25127557990994 1.01517493878549 1.54228647404563

MGAT4A 0.0765926450732694 0.816451721176949 0.652309220451902 1.02189788540932

MGAT5 0.0319418487898844 1.27703023216357 1.02133343382499 1.59674221938687

MZB1 0.509959876486244 0.962460378685636 0.858921605717725 1.07848024123883

MGP 0.0773720728276496 1.10848011100061 0.98876118982426 1.24269456480418

MICAL2 0.525281789773192 0.908174271991186 0.674686015096416 1.22246569493345

MICB 0.810490715077811 1.02293629695506 0.849866091768708 1.23125122623777

MIR155HG 0.227247679060206 0.852443932836404 0.65784490600356 1.10460786729215

MITF 0.249914211581026 1.12586979984426 0.919980396587818 1.37783675706871

MLPH 0.754465472261492 1.02079416735114 0.897273759574846 1.16131862876704

MMP12 0.129232844990754 0.926325056221653 0.839122822878028 1.02258940692497

MMP1 0.169629770057877 0.940661490720273 0.862025789182569 1.02647049685504

MMP14 0.0241407159328451 1.22533477802167 1.0269349377332 1.46206469666288

MMP16 0.0981301298465432 1.3666578074903 0.943851828107441 1.97886310875648

MMP2 0.162611445397142 1.0955376662866 0.963839613132369 1.24523080593479

MMP25 0.647601937032449 0.944944530043588 0.741228408506249 1.2046491400117

MMP3 0.117179484237004 0.931404278492939 0.852177919138363 1.01799625466956

MMP9 0.956461370049648 1.00309306397749 0.89782315682855 1.12070588439042

MMRN1 0.44993556027585 1.07386999232821 0.892600850549699 1.29195122289297

MMRN2 0.0174115224164115 1.26886385136718 1.04274011884044 1.54402371618418

MNDA 0.649788536427496 0.966475497171764 0.83419219192658 1.11973583027209

MPEG1 0.20529629650488 0.909903808270592 0.78622075039202 1.05304386826793

MPL 0.918553555730673 0.960104185478232 0.439956854632758 2.09520555769558

MPP1 0.268975688656029 1.09123551245391 0.934732659720961 1.27394173216974

MR1 0.213370177783826 1.17184983026675 0.912835506413424 1.50435868789955

MRC1 0.817384968065432 1.01498347384616 0.89461289174358 1.15154997394796

MRGPRF 0.249086700444729 1.08889335886608 0.942087393748423 1.25857617334734

MRO 0.795930800126221 1.07099788304383 0.636841764100623 1.80113260490111

MRPL27 0.998913014284796 1.0002524263328 0.695682101814517 1.43816394553643

MRPL55 0.0450338505285819 1.28356178014868 1.00554206991423 1.63845043658788

MRPS12 0.99130873434563 1.00136326084828 0.783677126710961 1.27951722207982

MRPS21 0.00934925409563091 1.29177067081362 1.06497249825214 1.56686812918919

MRVI1 0.31114583885577 1.09366178637425 0.919683445589859 1.30055195481764

MS4A14 0.992420818851336 0.997951392449003 0.653658331318529 1.52358951760319

MS4A1 0.667701961979172 0.958123094946082 0.788136279386793 1.16477300827124

MS4A2 0.0655107108670761 0.737638046608379 0.533586189689593 1.01972258337637

MS4A4A 0.483332196852291 1.05427350481914 0.909437073168651 1.2221765042973

MS4A6A 0.474510316441133 0.943795669645137 0.805431620931046 1.10592909800492

MS4A7 0.923299808798095 1.00807893410974 0.855765925005048 1.18750128709535

MSR1 0.524221244676596 1.04577129899222 0.911210325864125 1.20020326674718

MSRB3 0.203450167568847 1.09711624062306 0.951083604898374 1.26557122763932

MVP 0.200331528318561 1.21613712915251 0.901399168837842 1.64077089044817

MX1 0.260709689070969 1.08175830442457 0.943308659021284 1.24052823855729

MXD1 0.0219129147916332 0.762593935362815 0.604829568046582 0.961509722698206

MXRA8 0.0149293962208024 1.2167487786657 1.03895286761851 1.42497088802308

MYCT1 0.329093294259321 1.12033626487763 0.891749808071039 1.40751737206987

MYEF2 0.105161058385947 1.16874771075227 0.967843800542541 1.41135502508046

MYO1F 0.262971547606372 1.13170298382634 0.911279466144623 1.40544332576696

MYO1G 0.606007228256918 1.06428920272673 0.839910874835903 1.34860916911215

MYO7A 0.750420715902629 1.03927861943233 0.819659224204833 1.31774280934495

MYO9A 0.566569037235247 0.909347600843823 0.657040135334189 1.25854268969403

N4BP2 0.189168403370895 0.836699376360969 0.641194477430155 1.09181515288252

N4BP2L1 0.971662275303966 0.996197039907217 0.807321380227942 1.22926082056653

NAALADL1 0.116642965438096 1.17528429241325 0.960556579245175 1.43801333293534

NAIP 0.584615845931947 0.793759916311929 0.346766378678739 1.81694317408792

NAP1L3 0.363780096819624 1.12094772110317 0.876142486925806 1.43415461776687

NAPSB 0.72441753499243 1.03329395074362 0.861291559056044 1.23964571278689

NBEA 0.053143566203159 1.30772526264166 0.996370017260939 1.71637577699544

NBEAL1 0.279060967478761 0.82470113384708 0.581747307514566 1.16911922304278

NCCRP1 0.135496626529701 1.15180143025105 0.956732960331189 1.38664244855651

NCF1 0.201012102885048 1.16281670995179 0.922773065093503 1.46530360723748

NCF1B 0.387320448342476 1.13326192617899 0.853400116211685 1.50490088872727

NCF1C 0.29105521780926 1.11235743462077 0.912847270454651 1.35547216101093

NCF2 0.195370464085187 1.09935497058594 0.952509360734462 1.26883934287017

NCF4 0.898696050961713 1.01147746392983 0.848503325497699 1.20575444938631

NCKAP1L 0.776096459028968 0.97272686827335 0.803955829946052 1.1769272950286

NCOA2 0.902067495148367 0.983176217093475 0.750347321915942 1.28825071486898

NCR1 0.162217450119725 0.628060146460463 0.327139646248659 1.20578337751246

NCR3 0.768400798457124 1.0483713739247 0.765538507500927 1.43569856629771

NEGR1 0.414615464826066 1.13197645194626 0.840412499136708 1.52469256356503

NFAM1 0.614952552029908 1.05697884719291 0.851714242559516 1.31171257634004

NFATC2 0.611263167832927 1.05320318093312 0.862386904920698 1.28624047280685

NFKB2 0.085986613732053 1.26357707191246 0.967431225909319 1.65037779834132

NFKBID 0.464753957712824 1.13960666121329 0.802770840172554 1.61777592968192

NFKBIE 0.0871964880177987 1.33414982657261 0.958802952625387 1.85643541759008

NFYA 0.201104281315187 1.27667292111845 0.877902679135819 1.85657680088359

NFYB 0.862670283560492 1.03069200535516 0.731757735186503 1.45174551469864

NFYC 0.143659447275112 1.45761330358491 0.879644998213859 2.41533408034131

NHLRC2 0.180190343513542 0.824560495404146 0.621870645872628 1.0933142046399

NHSL2 0.58170341357921 1.10150327794107 0.780926937783012 1.55367859989491

NID2 0.221187169949813 1.12386926604737 0.932104046858206 1.35508705430821

NIPAL4 0.277446796757511 1.1766831769603 0.877293516255929 1.57824408055635

NKG7 0.62652624212375 0.96706889538977 0.845053774800305 1.10670146246183

NLRC3 0.561220145729718 0.910306912105991 0.663006728210836 1.24984957010034

NLRC4 0.807057482920034 0.958237044578535 0.680437566041733 1.349452586729

NLRC5 0.163152105922769 0.856260600775588 0.688473449352137 1.0649389851279

NLRP12 0.187808609189053 1.52833157841426 0.812985824200455 2.87310964600817

NLRP3 0.397902463914698 1.12387873414729 0.857291340117696 1.47336541262053

NMI 0.0498556793322403 0.782098249612487 0.611772437198286 0.999845097383262

NOD2 0.582817548604491 1.0622065571455 0.856422264692404 1.31743745644928

NOTCH4 0.00140295672103086 1.52022468885933 1.17566471619612 1.9657671722045

NOVA2 0.0202845715811048 1.51231492329855 1.06646460829207 2.14455914378093

NOX4 0.0523663850057296 1.24506425205882 0.997759418838955 1.55366610676416

NR1H3 0.158959061970765 1.23474276291295 0.92077344998945 1.65577068993829

NR5A2 0.453749212026923 0.91809675032137 0.734174212093894 1.14809486504119

NRP1 0.1884789621948 1.12797784117798 0.942657135646947 1.34973147932023

NRXN3 0.268483364156776 1.11955926542006 0.916587509063367 1.36747766731922

NT5E 0.498445620370028 0.950730466852959 0.821346031540913 1.10049648490622

NTM 0.0479245118570109 1.17226853720065 1.00145328698167 1.37221929487332

NTNG2 0.129021197836574 1.34793604277343 0.916735309552774 1.98195875786007

NTRK1 0.0659122073763695 1.68539339403951 0.966253144160402 2.93975849893895

OAS1 0.424536207150071 0.91411032405982 0.73329143040746 1.13951650040209

OAS2 0.887698443374122 1.00961650576158 0.884033720622047 1.1530391487657

OAS3 0.98031123442604 1.00244038675745 0.826016873794054 1.21654503785956

OASL 0.405170651945792 0.928189333708312 0.778817823303984 1.106209197364

TENM3 0.0281190105440979 1.2573451228036 1.02488358615724 1.54253300490993

OGFRL1 0.754802199113922 0.966866640287739 0.782587042605946 1.19453945594141

OGN 0.585875162410589 1.03986387812587 0.903451248071581 1.19687353062941

OLFML1 0.807417854462661 1.02594695543116 0.834976036622605 1.2605956448954

OLFML2B 0.0223111030410565 1.19289329442349 1.02540928954769 1.38773309973445

OLFML3 0.602674530704193 1.04143932622789 0.893799195764258 1.21346704646182

OLR1 0.224110578463387 1.08204983035891 0.95286664074828 1.22874679972038

OMD 0.422977188011347 1.07916588568431 0.895677684454288 1.30024341237703

OSCAR 0.047229479032168 1.18892500380663 1.00211910228966 1.41055355740342

OSM 0.103794328096897 1.11213060940328 0.978464453519115 1.26405664296066

OSMR 0.475523468173125 1.06494557143232 0.895905079453898 1.26588083505969

OTOA 0.533613561987495 1.56506242177823 0.381980321272155 6.41242558230418

OVOL1 0.680793958935644 1.05447863860171 0.818987692862777 1.35768242790142

P2RX1 0.728840536767245 1.05062520750045 0.794672083776894 1.38901736850901

P2RX4 0.638598065106209 1.08764819479545 0.765978290183648 1.54440225108492

P2RX5 0.00515864857515812 1.37819779151018 1.10075113792514 1.72557546122902

P2RX7 0.796018378635321 1.03327729017749 0.806166924432956 1.32436834858675

P2RY10 0.0641683956574589 0.79945859251742 0.630768832674422 1.01326192424576

P2RY11 0.054387795910332 1.30199255355071 0.9950424915632 1.70363037144112

P2RY12 0.701469510078366 0.925548213959694 0.623161989376447 1.37466583483559

P2RY13 0.0793857271432225 0.83786201049302 0.687600681517651 1.02095993720941

P2RY14 0.247673073845199 0.87689327086703 0.701804684913112 1.09566354431943

P2RY8 0.351112682207703 1.12109686812297 0.881645159419647 1.42558281445391

P4HA3 0.0182307911003173 1.26414846788586 1.04060902610756 1.53570775263771

PABPC5 0.78336522937926 0.924739969190146 0.5293958575798 1.61532055525933

PADI2 0.349605856453936 0.946111014903829 0.842423705548883 1.06256037980215

PAFAH1B2 0.493619239654702 0.877969451400846 0.604861801923008 1.27439086935633

PAG1 0.569973093395333 0.93839167600591 0.75354126812918 1.16858754104257

PALM2-AKAP2 0.325167792658771 1.57532112578568 0.637089241466378 3.89527320165497

PARM1 0.13192974818879 0.887746436296134 0.760351095483119 1.03648661761412

PARP12 0.88830380373002 1.02005153119715 0.773218456103583 1.34568066512661

PARP14 0.508245692601273 0.926188917419377 0.737979314008019 1.16239831451584

PARP15 0.485082988043799 1.11538916610773 0.820886521616257 1.51554808016701

PARP9 0.197442001205333 0.859483930738293 0.682707169862654 1.08203437697301

PARVG 0.284685393036874 1.14222017138369 0.895266897902783 1.45729382262658

PATL2 0.621062891344807 1.11680962549595 0.720696972209616 1.73063546496716

PATZ1 0.0645647805061056 1.37970868390972 0.980734817440473 1.94098957088513

PBX4 0.0173391553728305 1.41201815359373 1.06271159577728 1.87613956034795

PCDH12 0.00900434155131301 1.36991915536209 1.08173015643445 1.73488598895477

PCDH17 0.26812238122631 1.14171397771675 0.90299956706345 1.44353425456539

PCDH18 0.179598848025703 0.874456727520039 0.718865695641986 1.06372382621786

PCDHGA12 0.0969424248187385 1.30775926867482 0.952646468658848 1.7952455197914

PCOLCE 0.0546516783871483 1.17318142323664 0.99681932055261 1.38074636340769

PCYOX1L 0.863539694882238 0.972743073690815 0.7097933931024 1.33310495224199

PDCD1 0.704134847680595 1.04139912013772 0.844675931791467 1.28393871141029

PDCD1LG2 0.287082556226938 0.896669676374666 0.73353811018967 1.09608007731453

PDE1A 0.681399656717055 1.05637456752304 0.81304071011618 1.3725354868762

PDE1B 0.0120096535881406 1.43682626022672 1.08288982240094 1.90644482880062

PDE3A 0.345818934140682 0.928832137259526 0.796668766980203 1.08292075071086

PDE3B 0.0831515308949814 0.80249125047864 0.625670887859477 1.02928267814725

PDE4B 0.557639982291138 0.941528289131077 0.769781599887066 1.15159354206979

PDE6G 0.857130916582308 1.03208359835755 0.731814033191228 1.45555633765815

PDGFA 0.693719499482739 0.958718746526093 0.77726714336804 1.18252989693833

PDGFB 0.0110333544931181 1.3346678110092 1.06829882044916 1.66745308676382

PDGFC 0.708624887240575 1.03562214450262 0.861935724329001 1.24430766229018

PDGFRA 0.250469185473082 0.901259559424198 0.754826143145445 1.07610050450649

PDGFRB 0.0361497635669456 1.19133626407738 1.01136752820339 1.40332970411565

PDGFRL 0.162875499370897 1.12228815266931 0.954402585639438 1.31970587315417

PDIA2 0.00636930797893381 1.24125203064537 1.0627437416684 1.44974422635742

PDIA3 0.523662476827007 1.09169591411306 0.833678129244946 1.42956847143218

PDZK1IP1 0.137612027139842 0.899842600780271 0.782822306213923 1.03435568934558

PDZRN3 0.191749672902424 1.1324477785476 0.939539523137389 1.36496436770958

PECAM1 0.321503931902732 1.09870095647142 0.912136518652619 1.32342447327334

PEG3 0.721405048137484 1.07504587793237 0.722249651402811 1.60017195912056

PFDN2 0.00158194109119603 1.69412455165966 1.22154267604425 2.34953559365623

PGLYRP4 0.957072195397398 1.01261759151519 0.641456572924579 1.59854061822293

PGM5 0.103019625300237 1.115660576205 0.978118726329792 1.27254339150481

PHACTR1 0.231947481114857 1.22734723576252 0.877187095410474 1.71728613543841

PIK3AP1 0.893509711103961 1.01181940249179 0.851898571690014 1.20176102799169

PIK3CG 0.731130975667053 0.956538577566518 0.742390597341906 1.23245910393931

PIK3R5 0.345660525734429 1.11717058711366 0.887381746182087 1.40646359481884

PIK3R6 0.293826112633797 1.18073321081667 0.865846288558939 1.6101367339066

PILRA 0.355212608244186 1.08011037718489 0.917311417404823 1.27180192546063

PIM2 0.855175877737838 1.02319658674583 0.799860110835405 1.30889294383598

PIP4K2A 0.320403969314993 1.15749087999709 0.867415286648205 1.54457173847318

PKD2L1 0.664443202935223 1.13281422289895 0.644845067992915 1.99004090640926

PKHD1L1 0.567733661635635 1.27936730838225 0.549521987152222 2.9785536302915

PKIB 0.382716312585714 0.936860458774619 0.809236389106318 1.08461202564641

PKP3 0.0350068427922783 1.37732533397329 1.02277728882325 1.85477825557433

PLA1A 0.0308990609677597 1.19997358188339 1.01689891731535 1.41600760183671

PLA2G2D 0.917760784649362 1.00867086359364 0.856204620712732 1.18828710620111

PLA2G4E 0.905508393391995 0.96842448756305 0.57015930196591 1.64488413129114

PLA2G7 0.944727322021775 0.99430923088125 0.846160627978519 1.16839618144081

PLAC9 0.126964328287355 1.20719135235107 0.947888136289949 1.53742926554091

PLCB2 0.0298279398161106 1.29933476264054 1.02593323831178 1.64559521259323

PLCB4 0.536297054715226 0.966266183550088 0.866690518861332 1.07728227914518

PLCL1 0.70216961494064 0.932603939768812 0.652198714907974 1.3335661181041

PLCL2 0.664182047103624 0.956562984091738 0.78278872994255 1.16891404734665

PLD4 0.890549588606514 1.02578445029609 0.713811902956475 1.47410506060644

PLEK2 0.586741271071424 0.933470238769315 0.728265774083954 1.19649545217759

PLEK 0.879033022335372 0.989780930033499 0.867144613196015 1.1297611431238

PLEKHM3 0.58593512807861 1.1022630933347 0.776501980693321 1.56468876723656

PLEKHN1 0.0407524122925215 1.26260185743758 1.00985784976182 1.57860183072383

PLEKHO1 0.316259761929412 1.10939073758849 0.905549343463862 1.35911733306475

PLEKHO2 0.1620582699613 1.15938115919024 0.942311033275914 1.42645541102533

PLIN3 0.949868217788136 1.00889806660575 0.765443014491162 1.32978587501707

PLVAP 0.0300432925952668 1.24847727766406 1.02166723149049 1.52563913650193

PLXDC1 0.0412171011442885 1.30722027564505 1.01073684626315 1.69067235984847

PLXNA4 0.100748154027681 1.27436395235526 0.954008212808704 1.70229507593154

PLXNC1 0.584220398189444 0.945793699294845 0.774655691472162 1.15473975273565

PLXND1 0.0054029973995251 1.30974828240424 1.08299445129219 1.58397908799447

PML 0.567219950099945 1.080294203812 0.829163406514011 1.40748561456212

PMP22 0.37100059628507 1.08482446367083 0.90759284483855 1.296665265346

PNMA2 0.380066025305348 1.07770794914316 0.911864094474938 1.27371439525222

PNOC 0.811455612824706 0.961564170211801 0.696825913906813 1.32688184377534

PODN 0.0559325370205574 1.15739857515407 0.996305513770485 1.34453884200552

POSTN 0.0755600471470803 1.09985355575022 0.99025112731235 1.22158693964793

POU2AF1 0.116044391755474 0.872583894136915 0.736184749586054 1.03425485618287

POU2F2 0.244070419509468 1.18180666341405 0.892242685571311 1.56534428611825

PLPP3 0.255472462554454 1.16005032976119 0.898145851497689 1.49832765506294

PLPP4 0.0321836237419591 1.1602773633873 1.01272168465767 1.32933221474769

PLPP7 0.122559179047317 1.22589686285893 0.946626519793252 1.58755653570279

PPBP 0.444834605002722 1.02691609599757 0.959279839918401 1.09932120361103

PPFIA2 0.771316869904392 1.09649719551945 0.589147586196764 2.04075536919956

PPL 0.0355604268029205 1.22093292856809 1.01357242347947 1.47071603521388

PPM1H 0.679359003841768 0.94988400916928 0.744370286138932 1.21213816252077

PPM1M 0.00811166824301458 1.43497365999309 1.09831320044774 1.87482896867171

PPP1R13L 0.0300087255540109 1.30499434937351 1.02609756480144 1.65969622218755

PPP1R16B 0.559786596644839 0.938685643113378 0.758860561638453 1.16112337513591

PPP1R9A 0.990733678069165 1.00107925086232 0.834470308234504 1.20095305563039

PRAM1 0.24905870762308 1.16357363653158 0.899325678857177 1.50546530524048

PRELP 0.0294012560875623 1.13049715855636 1.01235803305986 1.26242276326011

PREX1 0.135953101317788 1.17834501653709 0.949696179342966 1.46204334417605

PREX2 0.383344670570901 1.1575454079616 0.833076501310995 1.60838934885858

PRF1 0.990887259095568 0.999116793085198 0.858547558744224 1.16270130414784

PRG2 0.338795179171034 0.448800610002662 0.0869185296196726 2.3173653353332

PRKAR2A 0.00570672980358664 0.623885317654888 0.446500327273162 0.871741554955711

PRKAR2B 0.717219507581757 0.967206957084598 0.80752515144745 1.15846459538261

PRKCB 0.49979171339067 0.919659033641684 0.721079825418594 1.1729252550753

PRKCQ 0.868205278191835 0.983831215336954 0.811527196808281 1.19271894285024

PRKG1 0.810755952734055 0.972246780790879 0.772190237414435 1.22413332486992

PRL 0.0249377082126596 1.53642389699784 1.05557499523641 2.23631518548556

PRLR 0.324003697800942 0.910083525539059 0.754683756466862 1.09748224519255

PROCR 0.609558547316509 0.952827400035671 0.791545130580911 1.14697194030168

PROM1 0.707218563485626 1.02253286450629 0.910286992894042 1.14861957509827

PRRX1 0.260495122863222 1.08538811641475 0.941025359123244 1.2518975730388

PRSS27 0.286999409263865 1.26746010575211 0.819316061763705 1.96072699492185

PRTG 0.486157670534706 1.22815169745978 0.68876495381409 2.1899438750777

PSAP 0.192731540562711 1.22769000516657 0.901643435879851 1.67163946279396

PSMB10 0.460697083733743 0.92505172543571 0.752097033236969 1.13777964400235

PSMB8 0.628252357719943 0.941994286341232 0.739600583775506 1.19977357368997

PSMB9 0.353601131670914 0.920680456611543 0.773170727254686 1.09633289687029

PSMC1 0.839365474623171 0.963266823976071 0.67079821303508 1.38325200655301

PSMC2 0.917163121136211 0.979628342266138 0.664687961844572 1.44379279309937

PSMC3 0.0209337502843969 1.57002538559036 1.07059856378472 2.30243136389433

PSMC4 0.519774442870658 1.12600468567759 0.784525076711008 1.61611985366139

PSMC5 0.0133906221680587 1.67014538364346 1.11231120089027 2.50773848206599

PSMC6 0.843894363770862 0.971252081055035 0.726511164952875 1.29843924011123

PSMD10 0.461012109635619 1.14223031695196 0.802051752841963 1.62669066221871

PSMD11 0.684097009978534 0.9252564385713 0.636420760561507 1.34517842623851

PSMD13 0.633773010163432 1.09632541867027 0.750986001748754 1.60046847853851

PSMD14 0.526454491164005 0.883861575591351 0.603233363647064 1.29503991636625

PSMD1 0.960892663318794 0.990407275186529 0.673726913081977 1.45594090379324

PSMD2 0.0969253354602514 1.40924126110736 0.939880061302044 2.11299400186899

PSMD3 0.415243354738195 1.12762263663831 0.844638709196382 1.50541621738945

PSMD4 0.00139626872622585 2.07779363306421 1.3267703851579 3.25393634791479

PSMD5 0.166131616020383 0.814118424331517 0.608519788665394 1.08918201376764

PSMD6 0.104972303305382 0.691710747872473 0.442997458116188 1.08005982868824

PSMD7 0.923218439729386 0.983238065996217 0.697216787066419 1.38659468956803

PSMD8 0.977872771799205 1.00454663349278 0.72904184186188 1.38416464037857

PSME1 0.55748741735255 0.92338000095058 0.707463941647616 1.20519305078616

PSME2 0.817427912964997 0.970311651190131 0.751254619781345 1.25324314239738

PSME3 0.587519783386004 0.908086004574866 0.64095583506604 1.28654760061554

PSTPIP1 0.586947011540706 1.07597415807492 0.826168960943443 1.4013118908788

PTAFR 0.838768002932083 1.01684863655174 0.865681951996965 1.19441227493762

PTCRA 0.529088111871199 1.17258132328564 0.714216580959859 1.92511206876556

PTGDR 0.214600033228552 0.901418119947849 0.765138998028962 1.06196995456185

PTGDS 0.15308422449776 1.0986174829003 0.965632836776246 1.24991645661473

PTGER2 0.0445043068847732 0.851476885927435 0.727881324537181 0.996059196503879

PTGFR 0.230594974246395 0.843509247111302 0.638637032635442 1.11410365137474

PTGIR 0.0779237153384014 1.25957322128166 0.974527826991131 1.62799322485052

PTGIS 0.32192066486072 1.06923332915038 0.936582485297162 1.22067188967694

PTH1R 4.37058190485811e-05 1.76147299770526 1.34264894548858 2.3109444446148

PTPN22 0.5484767234829 0.927363354702272 0.724896881114174 1.18637948934588

PTPN6 0.000821703154717314 2.07896149594953 1.35403248113084 3.19200681067199

PTPN7 0.840542467453704 1.01623693492693 0.868668757958791 1.18887377777511

PTPRB 0.973733358613754 1.00525430827054 0.735865840483772 1.37326149510096

PTPRC 0.296680016758369 0.925675347137366 0.800689652732789 1.07017100242688

PTPRJ 0.0264059593700554 1.41856778511349 1.04183298793762 1.93153277373694

PTPRM 0.14194567121137 1.1843894887984 0.944950661626136 1.48449915761966

PTPRO 0.930851442053658 1.00473458909346 0.903061329511749 1.11785496901589

CAVIN1 0.00537031103416372 1.25956942761759 1.0706886400411 1.48177078158595

PUS10 0.313433905229574 0.843825753356094 0.606546529042339 1.17392791473262

PVR 0.1861604706661 1.23914429906136 0.901704272657603 1.70286272390697

PVRIG 0.589241363836315 1.17640728499139 0.652279282375536 2.12168949340328

NECTIN2 0.0403272676658655 1.37398440179627 1.01410908671978 1.86156811047401

NECTIN3 0.857341473639807 0.983231187528786 0.817670277698972 1.18231467438171

PYHIN1 0.209685846881127 0.793270608215561 0.552425678375622 1.13911840540984

QPRT 0.497737046082557 1.03672934800111 0.934078741683956 1.15066074522705

RAB20 0.106356780012441 1.31885631877412 0.942554774559458 1.84539088498427

RAB33A 0.754451895362003 0.947885643805533 0.677797431056107 1.32559840531212

RAB37 0.542665758485865 1.08471265500754 0.834873096969397 1.40931783309893

RAB39A 0.901292875420712 0.98186968262486 0.735333545107427 1.31106227925043

RAB39B 0.922436298381249 1.02692236376397 0.601568979881643 1.75303178266615

RAB42 0.654781752416267 1.0558619532928 0.832006396191418 1.33994698780512

RAB8B 0.507993640725491 0.929615491951568 0.748953852529956 1.15385608867242

RAD23B 0.672375564664181 0.911986420006725 0.595040952448781 1.39775124191687

RAD54L2 0.3745666860133 1.19577049302754 0.805854843911853 1.77434817547826

RAET1E 0.0428943950971934 0.684815455892836 0.474686883784939 0.987961169034921

RAET1G 0.809648015057198 1.03985911875044 0.756586237510993 1.42919198531247

RAET1L 0.693022696442573 1.03426008454947 0.874969746357027 1.22254961036766

RAI2 0.16312456192778 1.11108946653533 0.958198289732103 1.28837612827604

RAMP3 0.059081549854282 1.22387960314054 0.992283408611511 1.50952970692054

RAPGEF2 0.436993927214115 0.891849742239178 0.668265042668032 1.19024026688047

RAPGEF6 0.803435249372648 0.953062600146604 0.652702826456877 1.39164146833707

RARRES2 0.198217628996719 1.10830858585605 0.947607540910252 1.29626229050723

RASAL3 0.523587882329425 1.07460151564153 0.861444918215396 1.34050174654385

RASGRF2 0.237364019242971 1.14530150512611 0.914507771048371 1.43434050444473

RASGRP2 0.029816755732044 1.33270785764523 1.02849852942192 1.72689623078763

RASGRP3 0.147233420294398 1.22710191378311 0.930440868665737 1.61835013649978

RASGRP4 0.677893510670295 1.06696989867777 0.78578476346254 1.44877429242584

RASL12 0.296298306382047 1.11475107944005 0.909164121369771 1.36682689066141

RASSF2 0.981922284875089 1.00215058693116 0.832207023791732 1.20679803243021

RASSF3 0.364282204882009 1.14138487845289 0.857743703833942 1.51882133898254

RASSF4 0.274756698922189 1.1466917769761 0.896946293914576 1.46597632467596

RASSF5 0.111848424698259 1.17960107240244 0.962288237732217 1.44598950236799

RASSF6 0.183611241657292 0.857828595544102 0.684281654190401 1.07539036714904

RBM38 0.0589111010644603 1.22684073689175 0.992333113979572 1.51676707397279

RBP5 0.147358823196096 1.18122826449656 0.942947753650591 1.47972165737042

RC3H2 0.975206791800801 1.00511867624906 0.728419478642772 1.3869255051046

RCAN2 0.105977654141565 1.18252678978635 0.96500984186599 1.44907289842604

RCN3 0.00983276827932784 1.2572122564742 1.05666836164947 1.49581715057849

RCSD1 0.8437386453948 1.023239544171 0.81427228265212 1.28583421916941

RDH12 0.298790314057471 1.26819901498246 0.81010450200478 1.98533490138905

RECK 0.211885269525444 1.17752709797389 0.911061690855038 1.52192774691417

REL 0.398985898901794 0.874190669925342 0.639603879086081 1.19481659253925

RELB 0.328037357228739 1.1374802493046 0.878694593672167 1.47248125443774

RELN 0.839042691405007 0.959633682692854 0.644814067585601 1.42815867589055

RENBP 0.0148398638340275 1.2307298192863 1.04142305290455 1.45444820321191

REST 0.352919108939709 0.882734879186599 0.678481866057203 1.14847707199721

RFTN1 0.449660589769529 1.08168980768766 0.882444976006718 1.3259215836325

RFX5 0.876223693027775 1.02573365313631 0.745044041536902 1.41217091677694

RFXANK 0.0458929022001327 1.47424493257477 1.00710472692472 2.15806565406462

RFXAP 0.750983557583378 1.04846548866023 0.78272317355306 1.40442996713834

RGL1 0.0931420186852502 1.20187348644199 0.969704221223954 1.48962935892862

RGL4 0.297249884916553 1.29253126978222 0.797801198805479 2.09405185886689

RGS13 0.171278206418571 0.699380117253966 0.419032925103203 1.16728905798919

RGS1 0.78124645620528 0.980812211030575 0.855458857012659 1.12453402688009

RGS18 0.218029970357377 0.834895257005593 0.626521527369977 1.1125716511235

RGS5 0.984734611220527 0.998331650700937 0.841379937741033 1.18456126665813

RHOD 0.187492737236419 1.12200685209335 0.945475563630303 1.33149858607734

RHOH 0.609569855263318 0.934911648685995 0.722089719970162 1.21045870987456

RHOJ 0.145019430758582 1.16635605086084 0.948311924967383 1.43453477865575

RIF1 0.665357670627135 0.946790851281243 0.739011704697358 1.21298879350898

RIMKLA 0.10927845700734 0.804052588334533 0.615693702893223 1.05003601915932

RIN1 0.601415744021196 1.06882159682644 0.832626406094461 1.37201942849867

RIN3 0.497204868057319 0.915579268325643 0.709761386763166 1.18108058880278

CARMIL2 0.0392829249158009 1.28469227716759 1.01238155619402 1.63024922462906

RNASE1 0.00147946209600732 1.35744496475531 1.12431027970366 1.63892198230656

RNASE2 0.767625730747945 0.967267831929035 0.77566952924693 1.20619287390732

RNASE6 0.303782008269536 1.12006438826393 0.902380870716228 1.39026022666163

RNASE7 0.648444196321889 0.936403759352715 0.705970773979909 1.24205141749232

RNF125 0.243124321098304 0.885902388520735 0.722831621029117 1.08576191073292

RNF166 0.053710905938954 1.43837197773971 0.994212561306492 2.08095735948868

RNF180 0.381518480197016 1.15928313014772 0.832576942950453 1.614190000365

RNF222 0.403099806580972 0.528506377552979 0.118530510498677 2.35651554978572

LAMTOR2 0.0459074945408488 1.53403536955301 1.00780682109359 2.33503531211077

ROBO4 0.0248764502824728 1.31650560456661 1.03534290829468 1.67402219397054

ROCK2 0.283088566752378 0.881067398810883 0.69919732295623 1.11024418395259

ROR1 0.846525180487414 1.02411206552118 0.804575670397707 1.30355112804694

RPS6KA4 0.0573400263275174 1.38168689544776 0.989971670643511 1.92839728010714

RRN3P2 0.0467843912896618 1.34635349763504 1.00423948795702 1.80501539954551

RSAD2 0.728280337852983 1.02785100223629 0.880288810858718 1.20014893949129

RTKN2 0.982189234061608 1.00326166968797 0.753794314194955 1.33528995763262

RTN1 0.034696779137154 1.28604153197864 1.01826104771173 1.62424245304353

RTP4 0.135421747656705 0.862742079860574 0.710740153490271 1.04725178774121

RUFY4 0.805426440549501 1.06919163258775 0.627871599234642 1.82070784630033

RUNX1T1 0.94928912116213 0.989120014886344 0.706050521945503 1.38567761575039

RUNX3 0.115046995333225 1.14063428796591 0.968440978513621 1.34344436857719

S100A12 0.142428786878943 1.11533792043929 0.963965249072502 1.29048083213244

S100A16 0.114661754483595 1.1766157343931 0.961344862615814 1.44009152205213

S100A2 0.00355351211316523 1.19185778968506 1.05920123035493 1.34112853169266

S100A7 0.216413183756228 1.08510778226722 0.953296455056883 1.23514452706806

S100A8 0.195330576472863 1.07408926629965 0.963962462241384 1.19679738285413

S100A9 0.175152126680799 1.08515031111997 0.964242924454262 1.2212183961736

S100B 0.715059495262539 0.967703363053345 0.811327280156769 1.15421953848736

S1PR1 0.163593566473888 1.1444644060657 0.946571566434232 1.38372926379499

S1PR4 0.86108292245817 0.98364056924618 0.817718891807326 1.18322907683895

SALL2 0.0359581629214117 1.31787139524888 1.01825115915703 1.70565483652683

SAMD14 0.00699607591193737 1.73028025278217 1.16164770683342 2.57726136379938

SAMD3 0.0755939945188282 0.69930815819134 0.471334057261356 1.03754840665332

SAMD9 0.522395510388289 0.945052813338355 0.794788212650258 1.1237268064414

SAMD9L 0.181401181810699 0.88892804425955 0.747958273648784 1.05646677857615

SAMHD1 0.875344315759415 1.018334968292 0.811532001401794 1.27783760326766

SAMSN1 0.325899446137845 0.914844139138228 0.766005951796158 1.09260221405993

SARDH 0.00280761979777706 1.59077049718413 1.17318458485122 2.15699286147061

SASH3 0.444585050528829 0.935448938853403 0.788353750288622 1.10998992125247

SBNO1 0.515360217998421 0.884382332995077 0.6107583547145 1.2805917510198

SBSN 0.571134577198648 1.07491951208494 0.837142534765341 1.38023324520814

SCARF1 0.017573133316137 1.36812310185722 1.05624361843462 1.77209195792293

SCARF2 0.0114808864447221 1.22852172398251 1.04731228883772 1.44108461476366

SCEL 0.20103629974409 1.10107896817517 0.949981696240495 1.27620868797325

SCML4 0.843872857030321 0.976914616642308 0.774300885009036 1.2325469164332

SCN7A 0.495720480986374 1.10186672238369 0.833530023361041 1.4565885329492

SCUBE3 0.0404604512393676 1.19820800229054 1.007889951783 1.42446346866864

SDC2 0.370296301973305 1.08091782424666 0.911729121818779 1.28150271260764

SDCBP2 0.468592962005394 0.945430325240484 0.81231701306347 1.10035673943774

SDS 0.192331465472609 1.12920148480988 0.940668061829559 1.35552172444

SDSL 0.120128730561786 1.22927921378901 0.947538471202915 1.5947926457649

SEC24A 0.332607157980687 0.882901539867509 0.686272840375604 1.13586766551009

SEC24D 0.361932212701598 0.891388745655341 0.696178701509374 1.14133611694569

SECISBP2L 0.928734208065977 0.988509610673518 0.767344803075831 1.27341873754418

SECTM1 0.739704532156238 1.02233344090271 0.897438072562378 1.16461034620895

SELENBP1 0.485081067937262 0.957864922020996 0.848826152832508 1.08091062672445

SELL 0.625885093172808 1.03817077598842 0.893027170855913 1.20690455485626

SELP 0.324060167858926 1.08174366768207 0.925342303570698 1.26457999170125

SELPLG 0.169386218316901 1.13063688290471 0.949006947760075 1.34702887476402

SERINC5 0.98068153879919 0.996353490735944 0.741301485347763 1.33915862590777

SERPINA1 0.0579824532593028 0.89914851671577 0.805565842808165 1.00360270030056

SERPINE1 0.00165723955359906 1.22005153825707 1.07784980307193 1.38101408170328

SERPINF1 0.25182468341608 1.09587622242964 0.937032274963177 1.28164709687704

SERPING1 0.300988260913911 1.07506490237041 0.937276985084184 1.23310884903986

SFMBT2 0.720097719896947 1.05187712829645 0.797667668704738 1.38710083966402

SFN 0.748266690811821 1.03001262645915 0.859825411180114 1.23388538751042

SFRP2 0.0132028579362584 1.0951599518914 1.01919220721383 1.17679012038907

SFTPB 0.629260838515924 1.05470464490094 0.849616666119131 1.30929857232773

SGCD 0.481970330459601 1.07466614004565 0.879224664735082 1.3135519951645

SGIP1 0.988248172820069 0.997575788220942 0.722239182320311 1.37787796287585

POMK 0.486831990766489 0.92665193830306 0.747594899367573 1.1485950686491

PEAK1 0.949339435606866 0.990444714492195 0.736549162230905 1.33186049590264

SH2B3 0.852426791082316 1.02392435322358 0.79814923693243 1.31356522390948

SH2D1A 0.257908685823852 0.869253757826642 0.681903939678913 1.1080770347969

SH2D2A 0.0578859939598901 1.28695030036339 0.991601678804475 1.67026852717945

SH2D3C 0.0542791034352791 1.25054394191596 0.995900530255881 1.57029753790863

SH2D5 0.392320577568466 1.17557651630364 0.811501411808193 1.70299167145658

SHE 0.39754327617202 1.13379948047146 0.847602721938303 1.51663182366577

SEM1 0.103066876951075 1.30853296687393 0.947050572326866 1.80799059250757

SIGLEC10 0.891797681094778 0.987734931834153 0.826832765445425 1.17994875909379

SIGLEC11 0.379644206961239 1.37097738023702 0.678155158000136 2.77160610657951

SIGLEC12 0.429987168391044 0.930943897355105 0.779375833922272 1.1119879553632

SIGLEC14 0.551654382399207 0.942724894310466 0.77633241755974 1.14478051701904

SIGLEC1 0.0962184137480181 1.15702995505863 0.974337827138498 1.37397756672817

SIGLEC5 0.132353927769419 1.34901113197495 0.913446312680712 1.99226928712606

SIGLEC6 0.589335192103932 0.866132937337053 0.513995946337266 1.45951786290522

SIGLEC7 0.683949893829263 1.04866443760687 0.834214581506446 1.31824248470396

SIGLEC8 0.646542284509487 0.920585166923899 0.646354704011782 1.31116404707848

SIGLEC9 0.508926860755336 1.07493171825492 0.867486631131443 1.33198386861987

SIGLEC17P 0.794992417660788 0.94137674204208 0.596838789687786 1.48480659395704

SIRPB1 0.696565040952701 1.06077263345349 0.788575776317869 1.42692511446138

SIRPB2 0.823843667575589 1.03122241576362 0.786663209968997 1.35181060623805

SIRPG 0.119210538702475 0.833621302351631 0.663061289174244 1.04805466263891

SIT1 0.65971988212595 0.95977506867039 0.799462503428511 1.15223438058796

SKAP1 0.0111302044844629 1.23534505382218 1.04935231830154 1.4543041220636

SLA2 0.405627107894794 0.893182905169305 0.684409029745164 1.16564169584923

SLA 0.702206859693385 0.9633435269701 0.79550045395003 1.16659990116547

SLAMF1 0.0449042468489423 0.730734862964534 0.537794562323381 0.992894828919288

SLAMF6 0.663579812736292 0.959680879031856 0.797245279622336 1.15521209484694

SLAMF7 0.0615612470370495 0.874181579900399 0.759233854038271 1.00653234912077

SLAMF8 0.727519567849636 0.973240285161517 0.835518176448125 1.13366373031871

SLC11A1 0.00575665390086167 1.26311973233776 1.07013907204627 1.49090104258153

SLC12A3 0.809916311788312 0.853794032186542 0.235500458591937 3.09538356636691

SLC15A3 0.261060771580425 1.11168828579388 0.924256563908385 1.33712963805776

SLC17A9 0.831102446811345 1.0150636818283 0.884758144687339 1.16456037658849

SLC18A2 0.0718525594722529 0.605139017427298 0.350211800120545 1.04563361453506

SLC1A7 0.993434367788504 0.999416467869567 0.869697000342879 1.14848421445065

SLC24A4 0.878755185934944 0.886340463583149 0.188087912502719 4.17676716664838

SLC25A45 0.0266296727946341 1.47612995075573 1.04616836743477 2.08280014894828

SLC29A3 0.321172489576503 1.17177571082479 0.85670369014952 1.60272254253894

SLC2A5 0.499027993682954 1.07359778560288 0.873823006795099 1.31904538595157

SLC34A2 0.898946714553688 1.01930807538955 0.758788844732735 1.36927283494832

SLC39A2 0.651087087808485 0.977690152833308 0.886615972846837 1.07811957399999

SLC45A3 0.418058373485566 0.919818575381286 0.751362842546549 1.12604212466634

SLC6A12 0.540342714371773 1.10829143294877 0.797473015080785 1.54025261936069

SLC7A7 0.30305961735541 1.10995638311756 0.910092335864759 1.35371228157061

SLC8A1 0.620486179766028 0.918445203270595 0.655857408474239 1.28616613994366

SLCO2B1 0.404182491350573 1.0933738436635 0.886499303654297 1.3485248742775

SLCO5A1 0.092811173059607 0.77395621688863 0.574045029687555 1.04348647698699

SLIT2 0.170845889806785 1.13666395321363 0.946266639142695 1.36537091036601

SLIT3 0.134141188756641 1.13984609215912 0.960434693532422 1.35277195061734

SMAP2 0.340922318443546 1.17366620157565 0.844148708412359 1.63181242711578

SMPDL3B 0.605431596043598 0.946460561006304 0.768134388109025 1.16618603125632

SNAI3 0.260414604441835 1.1638840059533 0.893561554184621 1.51598507452574

SNED1 0.0242317849966203 1.3420530624246 1.03905792050269 1.73340329429556

SNRPF 0.37698485301378 1.15873497303173 0.835671614435731 1.60669180875966

SNTB1 0.00280342557672817 1.37243826904915 1.11511697614789 1.68913830803417

SNX20 0.408038800743389 0.928346976088197 0.778425719492884 1.10714238549768

SOD3 0.301557496258608 1.06153673865865 0.947833138592589 1.18888040694092

SON 0.479312681035475 1.11516714529284 0.82447718968442 1.50834708042874

SOX17 0.103024663533739 1.22089343102536 0.960454740244072 1.55195316079349

SOX5 0.691109973543404 1.10580714243298 0.673327730260885 1.81606873042644

SP100 0.999791798457678 0.999961548592908 0.749119465336144 1.33479791266089

SP110 0.884865632979097 1.02427231533831 0.740354137295476 1.41707018724986

SP140 0.452681075374413 1.09949613500395 0.858379585714646 1.40834168357133

SPAG4 0.166168865978484 1.18123510165819 0.93313055690122 1.4953066910841

SPARC 0.0592748671194168 1.15578094692739 0.994350166964591 1.34341969424936

SPARCL1 0.1131807609423 1.11348860065981 0.974812660905905 1.27189245023463

SPATA13 0.528620944073382 1.09600275962703 0.824095587531726 1.45762465821214

SPI1 0.610129986302918 1.04067576715479 0.892804596557814 1.21303816817107

SPIB 0.79813335138471 0.973591633207277 0.793060572062043 1.19521850113746

SPN 0.916576538013744 1.01144769403992 0.817419407306648 1.25153186801557

SPNS3 0.529174298257446 1.08518591154991 0.841240475187025 1.39987137728316

SPOCK2 0.528289150732232 1.06149405360427 0.881791418755181 1.27781876969032

SPON1 0.386465646691851 1.05008096932846 0.940141124480922 1.17287714943286

SPRR1A 0.454032707961666 1.03333989783286 0.948325588398231 1.12597546403528

SPRR1B 0.363967191597455 1.04789874454821 0.947220096332552 1.15927838004843

SPRR2D 0.177982183836467 1.08387438682243 0.964011680650879 1.21864051026489

SPRY1 0.174590507852378 1.25901332722306 0.902852531427311 1.75567382595626

SRGN 0.967125569746736 1.00278159681746 0.87869207691023 1.14439512696153

SSC5D 0.0517892250721207 1.15006257751761 0.998916327144585 1.32407880046093

SSTR3 0.204024364159378 1.62958943280915 0.767054153968923 3.4620263846858

ST3GAL2 0.0546387565816621 1.35724471818888 0.993942441741597 1.85333993970903

ST3GAL5 0.964663736267599 1.00565176225752 0.783722601718984 1.29042529169559

ST3GAL6 0.414473449177622 1.14125364512618 0.830926960848415 1.56747818265992

ST6GAL1 0.534721669685216 1.04975277079835 0.900561739890955 1.22365944608333

ST6GALNAC3 0.929017452337452 1.01812692071609 0.685715437621743 1.51168016616629

ST8SIA4 0.577580384873708 0.932954328346893 0.730786903074441 1.19105005182686

STAB1 0.0731988944904027 1.18003762915524 0.98456218315326 1.414322863552

STAC3 0.423252663358876 1.12454498679289 0.843741180578807 1.49880254327934

STAP1 0.984045713426436 0.996915150989514 0.736452200464045 1.34949670548369

STARD13 0.282982921277053 1.13479053379803 0.900877159783723 1.42943967622264

STARD8 0.140486548488221 1.21408198443777 0.938043157538117 1.57135101204175

STAT1 0.398380958916971 0.919499446415801 0.756765409540799 1.11722763923895

STAT4 0.139385802192598 0.788109536990667 0.574722891179619 1.08072368758586

STAT5A 0.476270853832263 1.10176815664852 0.84387275193429 1.43847880882781

STK17B 0.0518445585724738 0.784085208795894 0.613595501944092 1.00194609103982

STK33 0.0548365073825394 1.27920547311609 0.994909809312695 1.64473867594148

STRN 0.211099880517844 0.84740414666271 0.653714309985314 1.09848258912565

STXBP6 0.0407640952585374 0.804284916102853 0.652820768978004 0.990891002569142

SUCNR1 0.15779354310466 0.828836201607522 0.638762815553174 1.07546875360968

SULF1 0.407508036200304 1.0503069130811 0.935138493759634 1.17965907619829

SULT1C2 0.816721071979416 0.981028140956955 0.834322618992715 1.15353005113465

SULT1C4 0.536421711348454 1.04989599681587 0.899719067202773 1.22513976230046

SULT2B1 0.146305422325232 1.12508903523104 0.95968008624274 1.31900761028914

SUSD3 0.436215998685653 1.05596597615575 0.920695592505706 1.21111054715042

SVOPL 0.192273252732878 1.34146765617029 0.862591075215805 2.08619764828984

SYNE1 0.492160954656341 1.10130282551807 0.836234435700425 1.45039221265529

SYT11 0.252729107452075 1.12317716777107 0.92044595694074 1.37056058608281

SYTL3 0.176575075659221 1.24505523759708 0.906008103994129 1.71098088177583

TAGAP 0.635830503269798 0.95339244349681 0.782487560291172 1.16162504996065

TAOK1 0.834856391002264 0.973310384139178 0.754741832842039 1.25517503158119

TAP1 0.278083905996379 0.90144084673285 0.74732159712561 1.08734392701066

TAP2 0.954247203052358 1.00686534858523 0.797012579906489 1.27197218179491

TAPBP 0.937018553161913 1.01156698788892 0.760513310049154 1.34549620297971

TAPBPL 0.603539681271938 0.93764937840399 0.735390290810198 1.19553707440544

TARP 0.406701426935734 0.728165058826051 0.344180497946014 1.54054153579124

TBC1D10C 0.835662580066979 0.976011094530214 0.775928898157044 1.22768678793719

TBCEL 0.37327590842602 1.11905023777096 0.873605696130652 1.43345383415168

TBX21 0.507131939724795 0.890477543103236 0.632073781242702 1.25452166867637

TBXA2R 0.0104017012779513 1.46689580370478 1.09423506169063 1.96647262938379

TBXAS1 0.655954658368753 1.04186301740433 0.869869783584445 1.24786326358176

TCEAL7 0.618541589767362 1.05118651587866 0.863657027671701 1.27943507174837

TCIRG1 0.0862787431677027 1.25544256662448 0.968081932880014 1.62810190394082

TCL1A 0.217270914696138 1.12586277635921 0.932607564631259 1.35916438946369

TCN2 0.312072711780737 1.10340003285342 0.911757851967472 1.33532344127743

TEK 0.478335826753646 1.07960874880816 0.873579017354729 1.33422967739372

TESC 0.0095083742157495 1.16551711710707 1.03811033659902 1.30856047028679

TFEC 0.290381053696302 0.861650448243878 0.653807590651827 1.13556573153071

TGFB1 0.0566567711917918 1.17768743440087 0.995375643140924 1.39339123144421

TGFB2 0.00838766830092704 1.3332387693798 1.07655470098622 1.65112428987491

TGFB3 0.0222461114679161 1.17800961608297 1.023649248263 1.35564663183088

TGFBI 0.254450507162025 1.09420607888024 0.937247003265835 1.27745080953744

TGFBR1 0.381147765259064 1.11300953449067 0.87586358853437 1.41436433719101

TGFBR2 0.112886095302721 1.21859741922988 0.954358769526327 1.55599730161305

TGFBRAP1 0.29388085747204 1.2441887272532 0.827387157123023 1.87095675307147

TGM1 0.286887472578484 1.16129924582725 0.881905113025503 1.52920752861079

TGM2 0.762634231118918 1.0266028200368 0.865778645263554 1.21730116106836

THBS1 0.242197993543424 1.09832995948264 0.938575758246161 1.28527579079104

THBS2 0.0420939591908588 1.11239887236635 1.0038132540621 1.23273053651609

THEMIS 0.0601016504273974 0.711463740339826 0.498908461951385 1.01457620469797

THPO 0.0426023478196002 1.54343077249107 1.01458020618224 2.34794502687598

THSD7A 0.281027938898406 1.1871908318853 0.869026023715172 1.62184104140759

THY1 0.0341159438194731 1.19629309608393 1.01351844531833 1.41202873844943

TIE1 0.00353775912267069 1.31827946610811 1.09487457336278 1.58726925717587

TIFAB 0.26461855497222 0.699843446859062 0.373829280708196 1.31017251827816

TIGIT 0.100584113128905 0.810990545046573 0.631560392861824 1.04139789573352

TIMD4 0.14146210683732 1.22202181178407 0.935416172314216 1.59644161890158

PAM16 0.071152893691173 1.36802540544915 0.973363476671464 1.9227077600591

TIMM50 0.856663351280381 0.968926990643968 0.687913179095599 1.36473546622939

TIMP2 0.173054202969179 1.10910035068219 0.955603900340798 1.28725258179113

TIMP3 0.0287827961843944 1.18845345035922 1.01804523773482 1.38738589535906

TLR10 0.83078265305694 0.959875952101039 0.659322956227887 1.39743631663178

TLR1 0.506863443622208 0.927862274222567 0.743813023027668 1.15745271092605

TLR4 0.359108356515317 0.930350225498963 0.797328513182073 1.08556451672803

TLR5 0.328174855992155 1.14984659858199 0.86913862778572 1.52121555526644

TLR7 0.432303163379876 0.889561027369141 0.664233489675667 1.19132629371101

TLR8 0.25772339028525 0.881855583468774 0.709316478473697 1.09636430802854

TLR9 0.972617038101066 0.95393322256002 0.0645700438347022 14.0930459244118

TM4SF18 0.419129666436327 1.12467631911317 0.845745550355783 1.49559973710986

TM6SF1 0.915369423168572 1.01453352174166 0.777484675590088 1.32385666117001

DCSTAMP 0.623673318381388 0.923541787361403 0.672153733568533 1.26894992976444

TMC8 0.00936456293073824 1.2908069662956 1.06473278411704 1.56488336706847

TMEM106A 0.235062285153931 1.15963325097718 0.90813925920072 1.48077430102016

TMEM119 0.930665875711745 0.992686483188749 0.841395362401581 1.17118122816004

TMEM140 0.422457481881051 1.10767500273412 0.862775414727751 1.42208956205504

IGFLR1 0.865128252951612 1.0313260406498 0.722461056635634 1.47223631274403

TMEM150B 0.145255959881508 0.905340447924806 0.791944845951651 1.03497273937525

TMEM156 0.471908735435733 0.880890159185447 0.623532981530228 1.2444690105172

TMEM170B 0.794465261865119 0.965874219983513 0.743827851234488 1.25420553597241

TMEM176A 0.860294899833766 1.01324487749278 0.875139571317475 1.17314450793234

TMEM176B 0.770492713315403 1.02600065093212 0.863481981236435 1.21910747252163

TMEM200A 0.35838163713125 0.937008245186938 0.815538226031078 1.07657056839767

TMEM204 0.0300827621774684 1.24984980806893 1.02172629590007 1.5289070556355

TMEM229B 0.799907147010064 0.97119031940072 0.774700389292193 1.21751666777841

TMEM233 0.239931629068746 1.25861270394369 0.857608330118249 1.84712051282201

TMEM26 0.412799605914069 0.811417312105061 0.492094971345986 1.33794916168914

TMEM47 0.424098452540057 1.07017765170264 0.906212218472957 1.26381015711052

TMEM79 0.0113079277167191 1.58835281785337 1.11035561579276 2.27212312713123

SYNDIG1 0.363353351298939 1.09210779684927 0.90314562767694 1.32060589498374

TMIGD2 0.441364873822911 0.897754824404442 0.682194399756691 1.18142823369541

TNF 0.663066031617343 1.06190645967447 0.810466071264836 1.39135414680412

TNFAIP6 0.0853061807990664 1.12760529893741 0.983438853798557 1.29290571069117

TNFAIP8L2 0.964340520150993 0.995418248835631 0.813900737933178 1.21741810018649

TNFRSF10A 0.574487769909253 1.07838221442855 0.828642978395499 1.40338870987305

TNFRSF10B 0.884568082304438 1.02060467414831 0.774959876525716 1.34411333082587

TNFRSF10C 0.707651078016842 1.0440512502327 0.833439748262698 1.30788460159796

TNFRSF10D 0.516535930125724 1.07695900357889 0.860820611124312 1.34736631581669

TNFRSF11A 0.0156057218647754 0.79874664277018 0.665740205487172 0.958326076866221

TNFRSF11B 0.798068997062632 1.01785476694961 0.888806417296656 1.16564001613892

TNFRSF12A 0.314403244676467 1.12446605496752 0.894728790046217 1.4131923805748

TNFRSF13B 0.341937477749657 1.21697248185998 0.811708349202177 1.82457408878463

TNFRSF13C 0.0686206815283246 1.24352207445072 0.983494018727909 1.57229949567598

TNFRSF14 0.336754829235057 1.16772881536768 0.851020578597744 1.60230036797329

TNFRSF17 0.0303937385028834 0.844836781946475 0.725234035142098 0.984163943698298

TNFRSF18 0.926681153476022 1.0088116050622 0.836872429673304 1.21607645134809

TNFRSF1A 0.963654669664551 0.99155751301961 0.688563461471346 1.42788044478098

TNFRSF1B 0.438116520992832 0.913162898948488 0.725811695686953 1.14887440498845

TNFRSF25 0.125877886465402 1.17881953465773 0.954894235800482 1.45525592593588

TNFRSF4 0.239415336455018 1.13081173430376 0.921387018755058 1.38783719806131

TNFRSF8 0.645878879507826 1.08586240542907 0.76414938010436 1.54301919784739

TNFRSF9 0.332910180093433 0.869905796633588 0.656073128487888 1.15343253999876

TNFSF10 0.040287411795244 0.776860549947819 0.61029898885978 0.988879754155856

TNFSF11 0.204041628356021 0.88783245613766 0.738922609070859 1.06675105145665

TNFSF12 0.410407781508717 1.09211540059516 0.885425497617409 1.34705410158913

TNFSF12-TNFSF13 0.314801386116153 0.3488043582528 0.0447399173843686 2.71937203841719

TNFSF13 0.700524177256358 0.960857545180921 0.783953609848835 1.17768093740793

TNFSF13B 0.82200598903481 0.980241010552402 0.823801129425786 1.16638883396354

TNFSF14 0.642786679782828 0.927132931886489 0.673425935195709 1.27642169459753

TNFSF15 0.855415236606175 1.02462695331801 0.788707300464844 1.33111534893489

TNFSF18 0.212632595819701 0.762581787007868 0.497938563836824 1.16787697139819

TNFSF4 0.743312280420031 1.03201320380636 0.85462721309067 1.24621734075026

TNFSF8 0.659835064230304 1.06849560550252 0.795511832764593 1.43515509380996

TNFSF9 0.353806071044945 1.0556599167903 0.941454678512527 1.18371907363449

TNIK 0.342475667857254 1.09474061963663 0.908126518652881 1.31970270624867

TNIP3 0.0957829747759006 0.766824863380747 0.561072132953268 1.04802989947797

TNN 0.194247065387864 1.4049036102446 0.840857147842822 2.34731209592721

TNNT2 0.331881807487217 1.19096602674115 0.836740395570053 1.69514951633867

TNS3 0.472196525730097 1.09784998627437 0.851158811906566 1.41603961035527

TOX 0.146129243701608 0.885946027510975 0.752455984208917 1.04311797651217

TPK1 0.00980856362422275 0.635685821375882 0.450729152414565 0.896539443551804

TPSAB1 0.649948335129389 0.971333297945931 0.856674615153233 1.10133807983765

TPSB2 0.715539122392129 1.02361272675772 0.902867281814054 1.16050612917898

TPSD1 0.84869341167599 0.985102777582805 0.84433872620028 1.14933432790476

TPSG1 0.0832317288565508 0.908494028135152 0.815013774210344 1.01269625774965

TRAF1 0.0199655949303604 1.40261019093487 1.05481412064092 1.86508249104494

TRAF3IP3 0.784969780577998 0.959936257599124 0.715621522628035 1.28766057128829

TRANK1 0.771606217317383 0.96489056894018 0.758005421908986 1.22824162350318

TRAT1 0.790753933437362 0.956332604822347 0.68765466916525 1.32998740800597

TREM1 0.220879002154498 1.10044986010007 0.944094099172135 1.28270041689295

TREM2 0.160771460646597 1.0936452780227 0.965043107614034 1.23938504374015

TREML1 0.543247472024061 1.15546297548781 0.725143589541119 1.84114526692294

TRIM21 0.411742033911819 0.872994023586472 0.631188829246071 1.2074335442977

TRIM22 0.485988816130632 0.94331651709211 0.800504460490404 1.11160661225254

TRIM61 0.275995110420564 0.626131315568555 0.269667269132303 1.45379313402424

TRPC4AP 0.704291854798272 1.06629754761443 0.765438573537257 1.48541045533448

TRPV2 0.118584371759948 1.1663929965595 0.96139489507444 1.41510281507964

TRPV3 0.678801119877683 1.10167695733294 0.696639908660301 1.74220871246438

TSHR 0.852977412031226 1.08756629126238 0.447607834203326 2.64249270792012

TSHZ3 0.312459835975861 1.12065451957791 0.898412873796881 1.39787239127914

TSLP 0.265581143976919 1.32553387514906 0.807088488662097 2.17701042556104

TSPAN11 0.363375702468188 0.906173868290855 0.732735364686598 1.12066527582496

TSPAN32 0.251113955975339 1.12984713244311 0.917217135722748 1.39176918198768

TSPAN4 0.0352159583281292 1.24456405191352 1.01527374665671 1.52563747897159

TTBK2 0.999691018704264 0.999930800262839 0.704464733306954 1.41932102210518

TTC16 0.00340400976327109 1.8669140044992 1.22936169555475 2.83510370690576

TTC21B 0.994680347593199 0.998800374070309 0.70182935180196 1.42143127055263

TTC24 0.391513526333844 1.29503943948492 0.716892295618105 2.33944088961842

TTC37 0.477154448642866 0.909962471024047 0.701533435669612 1.18031679827467

TUBA4A 0.309708600415267 1.15698247773104 0.873259230321376 1.53288783822419

TUBB6 0.0164115843991827 1.23769049463701 1.03984510533363 1.47317879620477

TXK 0.983515750922382 1.00463475920767 0.647900259272417 1.55778761462711

NME8 0.558267678272153 1.24457079849245 0.598267864191235 2.5890684844891

TYMP 0.474764629849078 1.05589434101226 0.90959705510223 1.22572171174895

TYROBP 0.673810837896639 1.02989143943045 0.897904410217355 1.18127983885876

UBA7 0.971778345184724 0.99546595055568 0.773905923408512 1.2804559685385

UBASH3A 0.43365953008437 0.882156108445305 0.644507668242286 1.20743233635945

UBD 0.0677885430494888 0.904202379978522 0.811583610298207 1.00739090043774

UBE2L6 0.0961097568479833 0.855527667278372 0.711894614892622 1.02814036539548

UBR1 0.917011677193762 1.01568053080264 0.757977680054421 1.36099910036594

UBXN11 0.429444047400234 1.12946965988849 0.835061200465646 1.52767451283483

UBXN1 0.00591152258351628 1.81555135151658 1.18737290362573 2.77606697940336

UCP2 0.409620876308513 1.07673416171435 0.903206984346849 1.28359996666887

UHMK1 0.932148256437187 1.01025257674216 0.79882702798974 1.27763612528555

ULBP1 0.221228054354824 1.20496701212174 0.893786869116131 1.62448739232141

ULBP2 0.00742379139562414 1.2166268846367 1.0539363190417 1.40443113087389

ULBP3 0.838274740262925 1.02733703647536 0.79292925863777 1.33104104182908

UNC13D 0.306811965106034 1.08505253059303 0.927811775617795 1.26894163782561

UNC5C 0.516913134124431 0.866700444824601 0.56231235962257 1.33585835026524

UNC93B1 0.0753691292037735 1.2729919955988 0.975631196199862 1.66098483440318

USHBP1 0.0285586775876506 1.57369603462643 1.04868780891932 2.36154095464409

USP12 0.978258784877583 0.996860237220424 0.795080306419956 1.24984900836706

USP51 0.351361665360045 1.2245973750893 0.799717735070297 1.8752100463844

UTS2 0.0429544110810416 1.20468016079037 1.00592260693916 1.44270968739616

VAMP5 0.32439025081268 1.08966012497448 0.918596412265919 1.2925798229829

VASH1 0.0997722030764483 1.19564270471455 0.966486345676744 1.47913261654632

VAV1 0.381995906342066 1.0952480019241 0.893156722186186 1.34306573070685

VCAM1 0.925715035301921 0.991599359610175 0.830461309666709 1.184003732063

VCAN 0.207173440898529 1.09257148579817 0.952149854778296 1.25370228813108

VEGFA 0.00105437244187643 1.57061650403834 1.19881207176869 2.05773386909437

VEGFB 0.0255016331093435 1.38023998906385 1.04028462922484 1.83128960468302

VEGFC 0.0298427699188762 1.24850889390051 1.02191985342813 1.52533924546002

VENTX 0.053879429291353 1.12436790463981 0.998046448015564 1.2666777057298

VGLL3 0.169584948072433 1.14488317778364 0.943870074720633 1.38870542236432

VIM 0.186001537211334 1.12361764784757 0.945368572727764 1.33547555416576

VMO1 0.0877990564000355 1.16736965960839 0.977340366039309 1.39434732210723

VNN2 0.85288884067814 0.984955720219102 0.839138810788269 1.15611119199814

VPREB3 0.845612871655384 1.01700293041756 0.858261913074016 1.2051041118362

VPS37D 0.0322085555156479 1.21559855568731 1.01671454282393 1.45338714688275

VSIG4 0.094091297855798 1.11008510761383 0.982331970664799 1.25445265240839

VWF 0.00364718098256312 1.27436630205167 1.08219773787344 1.50065871972352

WARS 0.938619366173832 1.00813081803962 0.820358680992827 1.23888217413782

WAS 0.413782626593993 1.08013731581845 0.897818966586702 1.29947869720217

WDFY4 0.614955219657098 1.06606945607425 0.830846350227877 1.36788719702835

WFDC12 0.55645761572814 0.865558219152443 0.534967355607562 1.40044251838786

WIPF1 0.733157721154464 1.03405712457199 0.852961381120496 1.25360205109572

WNT2 0.28947931370605 0.908994950456984 0.761881616443046 1.08451470953436

XAF1 0.201504277380973 1.13163341635228 0.936041370190948 1.36809571647875

XCL1 0.00177577589237168 1.44833306619407 1.14811599275156 1.82705291440446

XCL2 0.105398036721146 0.799254846254346 0.609386136474093 1.04828165759924

XCR1 0.836247505109673 1.03587289358094 0.741591872380624 1.44693151532413

XKR8 0.304806179354021 1.22664765382141 0.830364155318829 1.81205373207354

XPNPEP2 0.59675681496548 0.972434939311978 0.876777575213038 1.07852862336827

ZAP70 0.306520496024755 1.12554999707503 0.897246555137017 1.41194500961013

ZBP1 0.912771360803212 0.988039804614259 0.796669607743272 1.22537956263643

ZBTB10 0.71965032866913 1.0327307616405 0.866180938687191 1.23130489070222

ZBTB32 0.482643107669669 1.16364023252774 0.762201587290547 1.7765098017843

ZC3H12D 0.276774798072397 0.840644837463616 0.614831284766232 1.14939457419269

ZCCHC24 0.144694282471427 1.13281615082162 0.958032736185562 1.3394870374385

ZDHHC20 0.680831194310545 0.949134142311996 0.740107257930621 1.21719603537083

ZEB1 0.43975072829586 1.07491321931201 0.894922661588941 1.29110422458207

ZEB2 0.786514503116518 1.02934003699318 0.834978036037403 1.2689446500718

ZFPM2 0.254501965327801 1.18866566489099 0.8829852314634 1.60016953007137

ZKSCAN1 0.422690829543982 1.10685219694919 0.86359349454366 1.41863248580703

ZMYND15 0.590586210496056 1.07575358085025 0.824476822264243 1.40361224895809

ZNF185 0.13350096782407 1.12394419538749 0.964847894399348 1.30927430290103

ZKSCAN8 0.428665625129464 1.11037606738332 0.856782968027214 1.43902838528238

ZNF215 0.569675281447744 0.928410850238565 0.718665807398613 1.19937069214511

ZNF366 0.716410928134527 0.937225336827487 0.660584995428326 1.32971735366445

ZNF423 0.928798625494517 1.01083451636307 0.79804345960809 1.28036438012128

ZNF469 0.130817175007972 1.16377471556344 0.955920927437833 1.41682387079326

ZNF521 0.366725624617663 1.10743766100671 0.887327724471149 1.38214792482333

ZNF620 0.469420142560965 0.883497930859152 0.631626885619355 1.23580647310002

ZNF660 0.997762127407671 0.999404954636489 0.659326747270574 1.51489419697707

ZNF671 0.1596801011916 1.26187304224571 0.912461771574652 1.74508524559723

ZNF683 0.270917537440626 0.889053164615556 0.721118436631738 1.0960966872583

ZNF804A 0.973852801743937 0.987697294530392 0.471132154282139 2.07064182895581

ZNF80 0.895560845781544 1.0618249726051 0.433576245597443 2.6004013916727

ZNF827 0.813631563822314 0.965731249016752 0.722689951862652 1.29050755849544

ZNF831 0.84554538353431 0.941931108850486 0.515967062226667 1.7195559150451

ZNF835 0.666540610657942 0.865739643318296 0.449357156408746 1.66794969062677

LRP1 0.262176792747762 1.11572059879482 0.921358253883561 1.35108406456235

EIF2A 0.395085360901735 0.885720189156263 0.669626196583272 1.17154952641022

EIF2AK3 0.522184689232016 0.900358770321729 0.652847704207734 1.24170753771589

EIF2AK2 0.855966468108269 1.02542089092821 0.781952115526121 1.34469615552421

EIF2AK4 0.273722914996273 1.23284167876575 0.847462300850324 1.79347046278865

EIF2AK1 0.883626274363249 0.974157571540123 0.686077098360474 1.38320164957662

HMGB1 0.561104917592884 0.939700029516428 0.761906730935907 1.15898194571464

ANXA1 0.869941695333787 0.986915256229478 0.842959741037397 1.15545461492601

PANX1 0.212716622712035 1.23174402016095 0.887454733495784 1.70960080997695

P2RY2 0.232057348720063 0.869083347789802 0.690430809713278 1.0939631528309

TLR3 0.0137737094763027 0.750257647181583 0.596914345550838 0.942993817035855

VTCN1 0.00918903145662984 1.40706747739701 1.08823421212722 1.81931321758236

HHLA2 0.00564801416127457 0.845837256374582 0.751262377190348 0.952317973045533
