# Supplementary material for: Identification and Validation of Immune Molecular Subtypes and Immune Landscape Based on Colon Cancer Cohort
Source: Front Med (Lausanne). 2022 May 6;9:827695. doi: 10.3389/fmed.2022.827695 (PMC9121983; doi:10.3389/fmed.2022.827695)
Supplement: Supplementary Table 3 — 1553 significant high-frequency mutation genes identified by Chi-squared test. [file Table_3.DOCX]

Tag IS1 IS2 IS3 IS1.pvalue IS2.pvalue IS3.pvalue

DNAJC11 6 1 0 0.0416382254462463 0.890056051673045 1

EMC1 11 0 3 0.0140669844729861 1 0.766627874208378

TIE1 11 1 3 0.0312007424711093 0.991715000861244 0.809038983816177

POMGNT1 10 1 0 0.00272109672732751 0.969561061532513 1

LPPR4 7 3 10 0.904466197661763 0.939561585206236 0.0182961548166117

KIAA1324 9 1 2 0.0428170497206664 0.977978533343354 0.872738552446782

ATP1A1 12 0 1 0.000648772870822505 1 0.983283424470725

GABPB2 4 0 0 0.0463274983021047 1 1

NR5A2 2 1 7 0.982883938494014 0.957970850346816 0.00452284166020484

SLC45A3 7 0 0 0.00447375108354491 1 1

EPRS 13 1 4 0.0226878877662642 0.996913500187308 0.751017378945341

NLRC4 12 0 3 0.00783652701912318 1 0.809038983816177

CTNNA2 11 5 13 0.8795118041806 0.929605661497302 0.0211131571716205

KDM3A 3 0 5 0.809178333533066 1 0.0335780283108114

EIF2AK3 10 0 2 0.00965178637516871 1 0.872738552446782

ANKRD36C 8 0 2 0.0327516496754004 1 0.793128963052146

IWS1 7 0 1 0.0215160238576014 1 0.917693895037003

SAP130 9 1 2 0.0428170497206664 0.977978533343354 0.872738552446782

DNAH7 27 1 8 0.000273785622468814 0.999993367470317 0.790939762179268

MAP2 13 3 16 0.814519027447064 0.997363523744506 0.00257664620008072

IKZF2 8 0 1 0.0109413172055553 1 0.940037380594825

RQCD1 6 0 1 0.0416382254462463 1 0.887140871224982

STK16 5 0 0 0.0213233416842564 1 1

CCR8 4 0 0 0.0463274983021047 1 1

TLR9 12 1 1 0.00266588964761834 0.988511036350553 0.987885178408559

ZBTB20 19 2 5 0.004374119642335 0.997571736862308 0.868089053018954

PARP14 17 1 3 0.00103443305952306 0.998861639184504 0.949703306717485

MASP1 5 2 7 0.866192167096457 0.926551100093222 0.0485352203130862

UGDH 2 0 4 0.85760924819628 1 0.0450453231390708

ADGRL3 20 1 14 0.128352291904363 0.99999057334079 0.0489419735944764

KIAA1109 32 10 13 0.0429762107146214 0.962463756741368 0.754062357985789

FAT4 42 18 35 0.746340339768975 0.98582640323889 0.00700282129704549

ABCE1 2 0 4 0.85760924819628 1 0.0450453231390708

KIAA0922 7 3 9 0.867486309361261 0.923580742095784 0.0374715266911755

FBXO8 4 0 0 0.0463274983021047 1 1

TRAPPC11 6 0 0 0.00978296170678797 1 1

PDZD2 21 4 9 0.0466303454473859 0.992915641090312 0.575356912368988

C5orf42 14 1 17 0.699029241894504 0.999973138238664 0.000740181055957768

MIER3 6 0 7 0.621445429270755 1 0.0309628117503546

SYNPO 14 1 3 0.00611185694213879 0.996913500187308 0.899494782383858

RPP40 5 0 0 0.0213233416842564 1 1

PHACTR1 8 1 0 0.0109413172055553 0.942028759099056 1

OR2B2 9 1 2 0.0428170497206664 0.977978533343354 0.872738552446782

MDC1 7 0 7 0.50323080825299 1 0.0485352203130862

BTBD9 2 0 4 0.85760924819628 1 0.0450453231390708

UBR2 4 0 6 0.770176249907343 1 0.0248091206279707

SHPRH 15 2 5 0.030356903667279 0.992060914657446 0.741140107958061

GRM1 15 2 5 0.030356903667279 0.992060914657446 0.741140107958061

SASH1 5 1 7 0.81044724850275 0.984085326617886 0.0309628117503546

SYNE1 60 21 31 0.0489731897069979 0.993614739124032 0.420352918226651

ZAN 17 2 14 0.34056637729058 0.99972062174041 0.0286700060021326

DLD 8 0 0 0.00203914068725113 1 1

ZNF212 9 0 1 0.00548873408809398 1 0.956360538099567

ANGPT2 9 1 2 0.0428170497206664 0.977978533343354 0.872738552446782

ZFHX4 38 12 30 0.478157107815933 0.998485188693561 0.0102972381662713

VPS13B 31 5 14 0.014108122466998 0.999560131867264 0.461890711284131

SH3GL2 6 0 1 0.0416382254462463 1 0.887140871224982

DNAI1 3 2 7 0.96900618253642 0.876968871613257 0.0182515077914787

PCSK5 16 3 3 0.00998430132325486 0.962736190496774 0.960446763764443

IKBKAP 9 2 10 0.717636778362154 0.98939503064284 0.0271870539926272

KIAA1958 6 0 1 0.0416382254462463 1 0.887140871224982

MYO3A 12 5 15 0.898032578624759 0.962206633405786 0.00788690685778564

EPC1 7 0 1 0.0215160238576014 1 0.917693895037003

CTNNA3 15 0 7 0.030356903667279 1 0.360471214474326

TET1 16 3 5 0.0337541897751258 0.977420662701415 0.812900912715202

METTL15 2 4 0 0.85760924819628 0.0466863691393309 1

LRP4 12 1 3 0.0184626250071734 0.994031992145811 0.844842365134364

ARHGEF12 3 1 6 0.920063533056499 0.957970850346816 0.0248091206279707

SNX19 3 1 6 0.920063533056499 0.957970850346816 0.0248091206279707

PIANP 6 0 1 0.0416382254462463 1 0.887140871224982

ATN1 10 1 1 0.00965178637516871 0.977978533343354 0.976958233729919

TESPA1 9 0 2 0.0179431022115431 1 0.837233316198664

OR6C74 5 0 0 0.0213233416842564 1 1

DCN 6 0 1 0.0416382254462463 1 0.887140871224982

RNF17 15 2 4 0.0164664929781717 0.98939503064284 0.856112084063353

NOVA1 13 0 5 0.0226878877662642 1 0.545199966591366

MDGA2 11 1 11 0.536135403154565 0.999417866962482 0.0197231928754207

SLC24A4 8 0 2 0.0327516496754004 1 0.793128963052146

NUTM1 10 1 2 0.0247944696393595 0.984085326617886 0.90105594484024

CREBBP 21 3 9 0.0305468726444202 0.998012173298243 0.532719541183317

KIAA0556 12 2 11 0.523631809469409 0.996722425678259 0.0393240535266177

ABCC11 11 2 1 0.0140669844729861 0.926551100093222 0.987885178408559

SLC9A5 4 1 6 0.840736001584801 0.969561061532513 0.0429142007986453

DDX19A 5 0 0 0.0213233416842564 1 1

PFAS 11 2 0 0.00510898139591144 0.90469701404897 1

MAP2K4 7 3 10 0.904466197661763 0.939561585206236 0.0182961548166117

NCOR1 21 0 8 0.00319235679193738 1 0.523475384636954

NEK8 9 2 1 0.0428170497206664 0.876968871613257 0.976958233729919

SRCIN1 13 0 4 0.0107163099488403 1 0.705280176200014

KANSL1 19 3 8 0.0422429232533498 0.995402134881724 0.568776376722477

DDX5 4 0 0 0.0463274983021047 1 1

MYO9B 14 3 4 0.0470605620908893 0.952433770201283 0.856112084063353

DMKN 5 0 0 0.0213233416842564 1 1

CIC 18 3 7 0.0396841904048422 0.992078693950593 0.651876832457989

EPS8L1 13 0 3 0.00429449435643979 1 0.844842365134364

PLCB1 5 2 7 0.866192167096457 0.926551100093222 0.0485352203130862

NCOA6 14 0 5 0.0136206572299904 1 0.600319159643428

SON 4 2 8 0.953525872893264 0.926551100093222 0.0133904031876229

AGPAT3 4 0 0 0.0463274983021047 1 1

SGSM1 6 2 8 0.842024768729386 0.956960131592732 0.0349966212690756

MEI1 6 1 9 0.842024768729386 0.994031992145811 0.00980209135077389

SREBF2 9 2 1 0.0428170497206664 0.876968871613257 0.976958233729919

TAB3 9 1 1 0.0179431022115431 0.969561061532513 0.968273260289657

AKAP4 10 1 2 0.0247944696393595 0.984085326617886 0.90105594484024

HS6ST2 4 0 6 0.770176249907343 1 0.0248091206279707

AFF2 12 4 13 0.782329929348216 0.975813132897399 0.0211131571716205

CRYZ 3 0 5 0.809178333533066 1 0.0335780283108114

DYSF 17 5 17 0.714667609114608 0.992364600327103 0.0113969599964393

FRAS1 20 3 15 0.269313432658508 0.999534738085446 0.0470872226371549

APC 117 84 69 0.986178341831276 0.00121121378066061 0.803953659464755

SH3TC2 5 1 10 0.937293353596679 0.994031992145811 0.00216738531492487

OR11A1 4 0 0 0.0463274983021047 1 1

COL9A1 3 2 7 0.96900618253642 0.876968871613257 0.0182515077914787

FNDC1 18 4 19 0.702709038853318 0.998897979049274 0.0030570921343284

LPA 18 4 5 0.0242321024235943 0.961551533380204 0.890187128278008

MEOX2 4 0 0 0.0463274983021047 1 1

NACAD 9 0 3 0.0428170497206664 1 0.659707134958072

LRRD1 3 1 6 0.920063533056499 0.957970850346816 0.0248091206279707

PTPRZ1 17 4 5 0.0368596151824635 0.951843034689561 0.868089053018954

GSDMD 4 0 0 0.0463274983021047 1 1

PIGO 11 2 2 0.0312007424711093 0.943655533200879 0.941063952426992

ITIH5 14 0 4 0.00611185694213879 1 0.751017378945341

RET 7 1 10 0.819617606155775 0.996913500187308 0.00716008835023087

LRRC18 3 0 5 0.809178333533066 1 0.0335780283108114

SLC43A3 8 0 2 0.0327516496754004 1 0.793128963052146

SPTBN2 6 4 10 0.963022991528306 0.833022631136556 0.0182961548166117

KRAS 67 50 35 0.821843232751059 0.0190307865973761 0.920649549668517

TUBA3C 14 1 5 0.0266645160157442 0.998410848301568 0.651585258628802

UBE3A 6 2 8 0.842024768729386 0.956960131592732 0.0349966212690756

APBA2 14 1 6 0.0470605620908893 0.998861639184504 0.501165529800654

SCAPER 8 0 8 0.489294238525221 1 0.0349966212690756

CCNF 7 0 1 0.0215160238576014 1 0.917693895037003

TP53 97 68 37 0.311739947097629 0.000760386437336674 0.999975887571332

DCC 20 5 7 0.0445525762336056 0.962206633405786 0.796216507354531

CACNA1A 20 3 15 0.269313432658508 0.999534738085446 0.0470872226371549

SIN3B 4 1 6 0.840736001584801 0.969561061532513 0.0429142007986453

LSS 8 0 1 0.0109413172055553 1 0.940037380594825

SLITRK4 5 1 7 0.81044724850275 0.984085326617886 0.0309628117503546

ZBED4 13 3 2 0.0226878877662642 0.903877879164331 0.973733484103117

RPAP2 1 1 5 0.988247552057685 0.890056051673045 0.015948402149609

ERBB4 16 2 16 0.548576834841053 0.9997965739684 0.0056971059087254

COL6A6 12 7 20 0.989202467794591 0.939918834926978 0.000427077750916995

YTHDC2 10 1 11 0.628378023831211 0.999185483209602 0.0132375397681892

ATG5 1 0 4 0.957655037862292 1 0.0188689109478078

EZH2 3 1 6 0.920063533056499 0.957970850346816 0.0248091206279707

BTBD10 0 2 4 1 0.509971524650284 0.0450453231390708

NUMA1 6 3 11 0.963022991528306 0.939561585206236 0.00521869813587479

MMP8 1 1 4 0.977664195795495 0.848827071050437 0.0450453231390708

GALR1 2 1 5 0.949159299785454 0.920123595552516 0.0335780283108114

TAS1R3 10 1 1 0.00965178637516871 0.977978533343354 0.976958233729919

TP73 9 1 2 0.0428170497206664 0.977978533343354 0.872738552446782

CA6 7 1 0 0.0215160238576014 0.920123595552516 1

NMNAT1 4 0 0 0.0463274983021047 1 1

HTR6 6 0 0 0.00978296170678797 1 1

CSMD2 31 7 15 0.042389807877164 0.996934671284579 0.436104371614322

OSCP1 6 0 1 0.0416382254462463 1 0.887140871224982

SZT2 18 3 6 0.0242321024235943 0.989653086230853 0.770857521879112

EPS15 4 0 6 0.770176249907343 1 0.0248091206279707

JAK1 12 1 4 0.0370537811471727 0.995705739391037 0.705280176200014

CELSR2 20 3 4 0.00258822032220131 0.989653086230853 0.958903761010028

AMPD2 9 0 2 0.0179431022115431 1 0.837233316198664

OR6N1 7 0 7 0.50323080825299 1 0.0485352203130862

RCOR3 7 1 0 0.0215160238576014 0.920123595552516 1

MIA3 12 1 11 0.445831670983997 0.999584431039691 0.0283125738357982

GALNT2 8 1 1 0.0327516496754004 0.957970850346816 0.956360538099567

CAPN9 7 0 0 0.00447375108354491 1 1

KIDINS220 7 2 9 0.819617606155775 0.975173805854432 0.0252911634314708

DTNB 6 0 1 0.0416382254462463 1 0.887140871224982

MSH6 12 1 4 0.0370537811471727 0.995705739391037 0.705280176200014

KIAA1841 2 0 6 0.949159299785454 1 0.0053706264528876

GGCX 9 0 2 0.0179431022115431 1 0.837233316198664

NCAPH 3 0 6 0.875097348111752 1 0.0126373532473112

FER1L5 12 1 12 0.523631809469409 0.999703681263084 0.0142979633202099

AFF3 11 1 10 0.455360783967448 0.999185483209602 0.038811649179659

KCNJ3 2 3 9 0.998277937520503 0.773572497783455 0.00283038030605685

ZDBF2 11 5 13 0.8795118041806 0.929605661497302 0.0211131571716205

SP140 6 1 8 0.783532659638071 0.991715000861244 0.0223993563099247

GIGYF2 7 1 9 0.759395689219841 0.995705739391037 0.0162256258908508

SH3BP4 6 2 9 0.887095175734593 0.967252036222198 0.0162256258908508

SRGAP3 13 0 3 0.00429449435643979 1 0.844842365134364

TRIM71 14 0 5 0.0136206572299904 1 0.600319159643428

AMIGO3 7 1 0 0.0215160238576014 0.920123595552516 1

PXK 4 0 0 0.0463274983021047 1 1

CASR 13 1 2 0.00429449435643979 0.994031992145811 0.954809893468796

ABTB1 12 1 1 0.00266588964761834 0.988511036350553 0.987885178408559

MED12L 13 1 13 0.512041063558512 0.99984987173628 0.0103521630188767

AP2M1 6 0 1 0.0416382254462463 1 0.887140871224982

ABCF3 5 2 8 0.90752192082565 0.943655533200879 0.0223993563099247

CHRD 8 2 10 0.798809373946043 0.985874910600787 0.0182961548166117

FAM193A 14 3 4 0.0470605620908893 0.952433770201283 0.856112084063353

RGS12 10 0 9 0.37934729540708 1 0.0374715266911755

PHOX2B 6 0 1 0.0416382254462463 1 0.887140871224982

BMP2K 6 0 1 0.0416382254462463 1 0.887140871224982

SPATA5 9 0 2 0.0179431022115431 1 0.837233316198664

MAML3 7 0 1 0.0215160238576014 1 0.917693895037003

NIPBL 22 2 8 0.00712553579996501 0.999617079410581 0.653310085950871

BHMT 5 0 0 0.0213233416842564 1 1

ADGRV1 25 9 23 0.724266830039783 0.989278145297122 0.00971333172017103

RAPGEF6 13 2 3 0.0226878877662642 0.975173805854432 0.899494782383858

DOK3 6 0 1 0.0416382254462463 1 0.887140871224982

DST 29 8 9 0.0128256641488437 0.962074459641475 0.910872956228967

GRIK2 18 4 5 0.0242321024235943 0.961551533380204 0.890187128278008

ROS1 24 0 7 0.000276719141285509 1 0.764955727672481

SOGA3 7 1 14 0.952823429043638 0.999185483209602 0.000180986000812061

ULBP3 6 0 0 0.00978296170678797 1 1

EIF3B 10 0 1 0.00272109672732751 1 0.968273260289657

TNS3 8 1 11 0.798809373946043 0.998410848301568 0.00521869813587479

GTF2IRD1 4 1 6 0.840736001584801 0.969561061532513 0.0429142007986453

TRIP6 8 0 2 0.0327516496754004 1 0.793128963052146

MUC17 20 4 16 0.386174114384719 0.998549291778623 0.0354650747217132

EPHB6 10 0 3 0.0247944696393595 1 0.716992894282767

WHSC1L1 12 4 1 0.0370537811471727 0.714034999860102 0.995418328247917

CHD7 17 5 18 0.763595395121477 0.994014494614482 0.00600635778301201

EPPK1 14 1 6 0.0470605620908893 0.998861639184504 0.501165529800654

GLDC 10 2 1 0.0247944696393595 0.90469701404897 0.983283424470725

C9orf131 10 0 2 0.00965178637516871 1 0.872738552446782

TRPM6 12 0 10 0.290969037000119 1 0.038811649179659

SPTLC1 2 0 4 0.85760924819628 1 0.0450453231390708

PTPDC1 4 0 0 0.0463274983021047 1 1

ANP32B 4 0 0 0.0463274983021047 1 1

CNTRL 15 2 3 0.00803043203609599 0.985874910600787 0.936333132052609

NUP188 6 1 8 0.783532659638071 0.991715000861244 0.0223993563099247

ABCA2 18 4 6 0.0396841904048422 0.969439688566351 0.803306800812145

GPR158 19 3 7 0.0265028393670753 0.993955489456731 0.692731838582864

APBB1IP 7 1 0 0.0215160238576014 0.920123595552516 1

MKX 7 0 0 0.00447375108354491 1 1

PPRC1 16 1 4 0.00465600765926572 0.998861639184504 0.856112084063353

TRIM8 7 0 0 0.00447375108354491 1 1

TACC2 12 0 11 0.367243017581502 1 0.0197231928754207

MRVI1 6 1 8 0.783532659638071 0.991715000861244 0.0223993563099247

OR4A15 7 0 9 0.686045811152545 1 0.00980209135077389

CTNND1 7 1 10 0.819617606155775 0.996913500187308 0.00716008835023087

NRXN2 12 4 13 0.782329929348216 0.975813132897399 0.0211131571716205

PELI3 7 1 0 0.0215160238576014 0.920123595552516 1

PDGFD 9 1 1 0.0179431022115431 0.969561061532513 0.968273260289657

DSCAML1 22 5 18 0.432637048976545 0.998314134635273 0.0256008914920735

BCL9L 19 4 7 0.0422429232533498 0.980935680631798 0.730457413931678

RNF26 4 0 0 0.0463274983021047 1 1

STT3A 2 0 4 0.85760924819628 1 0.0450453231390708

THYN1 8 0 1 0.0109413172055553 1 0.940037380594825

VWF 23 4 8 0.013654705744713 0.99451747846409 0.760782670257109

METTL25 7 0 1 0.0215160238576014 1 0.917693895037003

MED13L 11 0 3 0.0140669844729861 1 0.766627874208378

VPS33A 12 0 0 8.51201451244521e-05 1 1

HIP1R 7 2 9 0.819617606155775 0.975173805854432 0.0252911634314708

DNAH10 28 2 14 0.0121213772359959 0.999992322150005 0.250864183195227

KBTBD6 5 0 6 0.646696684471791 1 0.0429142007986453

NAA16 4 0 6 0.770176249907343 1 0.0248091206279707

DCAF11 7 0 0 0.00447375108354491 1 1

INSM2 9 0 0 0.000926368622463118 1 1

LTBP2 20 1 9 0.01739015922239 0.999946341102905 0.398897656401766

SNW1 6 0 1 0.0416382254462463 1 0.887140871224982

SNRPN 7 0 1 0.0215160238576014 1 0.917693895037003

SLC24A1 8 0 2 0.0327516496754004 1 0.793128963052146

HCN4 11 3 12 0.744348623800949 0.98653069146967 0.0206272361340397

C15orf39 10 1 2 0.0247944696393595 0.984085326617886 0.90105594484024

SV2B 9 0 3 0.0428170497206664 1 0.659707134958072

SETD1A 13 2 4 0.0423335539281904 0.981243578061332 0.791203570759185

NKD1 5 0 0 0.0213233416842564 1 1

SALL1 9 4 11 0.87268096242383 0.925524095291037 0.0283125738357982

CHD9 16 2 6 0.0337541897751258 0.995586893186678 0.651167241301287

ZC3H18 14 0 6 0.0266645160157442 1 0.446520862687304

PIEZO1 18 2 3 0.0014954100042384 0.99407304064624 0.969029145013482

SGSM2 4 0 0 0.0463274983021047 1 1

DNAH9 23 4 19 0.36832601121367 0.999732551698486 0.0146805411576941

PGAP3 4 0 0 0.0463274983021047 1 1

KRT10 6 0 1 0.0416382254462463 1 0.887140871224982

RNF43 28 3 8 0.000719061152334918 0.999654648587382 0.864736583151562

INTS2 8 0 8 0.489294238525221 1 0.0349966212690756

UNC13D 8 0 2 0.0327516496754004 1 0.793128963052146

ST6GALNAC2 2 1 5 0.949159299785454 0.920123595552516 0.0335780283108114

DSEL 11 4 12 0.797756953567298 0.961551533380204 0.0288472946261702

NR2F6 4 0 0 0.0463274983021047 1 1

SSBP4 6 1 0 0.0416382254462463 0.890056051673045 1

PLD3 2 0 5 0.914164058222356 1 0.015948402149609

MEGF8 14 6 14 0.80114792703677 0.934603699554424 0.0378300864191242

EXOC3L2 6 0 1 0.0416382254462463 1 0.887140871224982

FUT2 6 0 1 0.0416382254462463 1 0.887140871224982

CPT1C 9 0 3 0.0428170497206664 1 0.659707134958072

TPX2 6 0 0 0.00978296170678797 1 1

ZNF341 8 0 0 0.00203914068725113 1 1

SLC19A1 9 1 0 0.00548873408809398 0.957970850346816 1

KREMEN1 6 0 0 0.00978296170678797 1 1

ELFN2 8 1 1 0.0327516496754004 0.957970850346816 0.956360538099567

CELSR1 14 4 13 0.638118864423434 0.985032375640627 0.0386927559058104

MXRA5 14 5 17 0.876048554077831 0.984488010176924 0.0041037446505711

FAM47C 12 3 13 0.728377233320583 0.992078693950593 0.0150029855867926

AMER1 23 7 20 0.59605038409416 0.993837008125207 0.0183816796296234

PIH1D3 4 0 0 0.0463274983021047 1 1

FMR1 7 1 0 0.0215160238576014 0.920123595552516 1

CRMP1 13 2 3 0.0226878877662642 0.975173805854432 0.899494782383858

DAB2 2 2 6 0.982883938494014 0.79854993786922 0.0248091206279707

DMGDH 4 1 6 0.840736001584801 0.969561061532513 0.0429142007986453

SDK1 35 9 9 0.00173769797388879 0.976217080802599 0.97355676102721

SLITRK1 9 3 11 0.830922495983734 0.970933591775118 0.0197231928754207

MAGEL2 4 3 7 0.953525872893264 0.773572497783455 0.0485352203130862

ADAMTSL3 20 4 6 0.01739015922239 0.980935680631798 0.857576200433114

SDK2 17 3 17 0.601010965665597 0.999374732089805 0.00589577431552852

SNPH 1 5 0 0.977664195795495 0.00607048063289141 1

CHD5 9 2 10 0.717636778362154 0.98939503064284 0.0271870539926272

KCNK3 6 0 1 0.0416382254462463 1 0.887140871224982

ABCA12 22 4 9 0.0323278286235972 0.99451747846409 0.616300537236606

SH3BP5 6 1 0 0.0416382254462463 0.890056051673045 1

SPOCK3 11 1 1 0.00510898139591144 0.984085326617886 0.983283424470725

ACOT12 3 0 6 0.875097348111752 1 0.0126373532473112

FSCN1 6 0 0 0.00978296170678797 1 1

ITFG2 4 0 0 0.0463274983021047 1 1

MDM1 6 0 1 0.0416382254462463 1 0.887140871224982

KLHL1 6 1 7 0.710106395034079 0.988511036350553 0.0485352203130862

ASB2 8 2 0 0.0327516496754004 0.79854993786922 1

MYOCD 12 0 4 0.0184626250071734 1 0.653890998104032

KCNN1 7 0 1 0.0215160238576014 1 0.917693895037003

POLD1 18 1 7 0.0138211211596331 0.999788959039115 0.561690417930081

PRL 7 0 1 0.0215160238576014 1 0.917693895037003

ESR1 4 1 9 0.953525872893264 0.988511036350553 0.00283038030605685

NEIL2 4 0 0 0.0463274983021047 1 1

OR4N2 2 1 5 0.949159299785454 0.920123595552516 0.0335780283108114

METTL2A 4 0 0 0.0463274983021047 1 1

TLE2 9 3 0 0.0428170497206664 0.667568072872785 1

COL5A3 7 4 10 0.932318426263042 0.862328475235952 0.0271870539926272

FAM21C 5 1 8 0.866192167096457 0.988511036350553 0.0133904031876229

SACS 22 7 18 0.54968645612798 0.987989760230618 0.040998620438515

USP7 12 2 3 0.0370537811471727 0.967252036222198 0.874748548477439

ARAP2 10 3 12 0.813936684996989 0.982528269533504 0.0142979633202099

DAPK1 13 2 12 0.512041063558512 0.998205228912824 0.0288472946261702

ELAVL4 5 0 6 0.646696684471791 1 0.0429142007986453

ADAMTS4 5 2 9 0.937293353596679 0.956960131592732 0.00980209135077389

HEATR1 14 4 2 0.0266645160157442 0.833022631136556 0.984939287865543

APOB 24 3 12 0.0354395069074722 0.999654648587382 0.323997274176602

KLHL29 8 1 0 0.0109413172055553 0.942028759099056 1

NEB 34 7 18 0.0439046177364307 0.999304959051991 0.274393476814792

MCCC1 3 1 6 0.920063533056499 0.957970850346816 0.0248091206279707

DLG1 9 1 2 0.0428170497206664 0.977978533343354 0.872738552446782

GALNT10 3 0 5 0.809178333533066 1 0.0335780283108114

ZNF318 13 1 11 0.361548187624799 0.999703681263084 0.0393240535266177

SERAC1 10 1 1 0.00965178637516871 0.977978533343354 0.976958233729919

OR52E4 8 0 1 0.0109413172055553 1 0.940037380594825

USP28 8 4 10 0.888379134590335 0.887185108329836 0.038811649179659

NOP9 7 0 1 0.0215160238576014 1 0.917693895037003

ITGA11 8 0 8 0.489294238525221 1 0.0349966212690756

MAU2 6 0 1 0.0416382254462463 1 0.887140871224982

MX2 2 1 5 0.949159299785454 0.920123595552516 0.0335780283108114

SEMA3G 9 0 1 0.00548873408809398 1 0.956360538099567

IFT80 11 0 1 0.00133495879775927 1 0.976958233729919

FAM160A1 3 1 6 0.920063533056499 0.957970850346816 0.0248091206279707

DYNC1I1 7 1 0 0.0215160238576014 0.920123595552516 1

ADCY8 11 5 12 0.842649713726937 0.91422260226332 0.0392305264057035

ADAMTSL1 12 2 11 0.523631809469409 0.996722425678259 0.0393240535266177

CLMP 9 0 1 0.00548873408809398 1 0.956360538099567

CACNA1C 22 6 6 0.0205904370997271 0.934603699554424 0.92994047000643

GCN1L1 21 2 6 0.00319235679193738 0.999026205379386 0.832152443132827

CDH2 11 2 11 0.612546253452738 0.995586893186678 0.0283125738357982

SLC2A11 7 0 0 0.00447375108354491 1 1

ARSA 5 0 0 0.0213233416842564 1 1

ZBED1 9 1 1 0.0179431022115431 0.969561061532513 0.968273260289657

SULT6B1 1 1 4 0.977664195795495 0.848827071050437 0.0450453231390708

RIF1 15 3 13 0.491063810243282 0.996513180514699 0.0386927559058104

SLC6A15 4 1 10 0.970254582736032 0.991715000861244 0.00104558770635555

RPGRIP1 5 1 8 0.866192167096457 0.988511036350553 0.0133904031876229

SUPT16H 14 1 5 0.0266645160157442 0.998410848301568 0.651585258628802

MROH9 12 1 4 0.0370537811471727 0.995705739391037 0.705280176200014

NDUFS1 9 0 2 0.0179431022115431 1 0.837233316198664

CDH12 10 0 10 0.465653388936302 1 0.0182961548166117

PCDHB4 12 0 4 0.0184626250071734 1 0.653890998104032

TMEM181 6 1 0 0.0416382254462463 0.890056051673045 1

PDE10A 2 0 5 0.914164058222356 1 0.015948402149609

COL22A1 13 6 14 0.854169378154575 0.921025472341148 0.0286700060021326

ZNF518A 13 1 5 0.0423335539281904 0.997784051416529 0.600319159643428

OR8H1 8 1 1 0.0327516496754004 0.957970850346816 0.956360538099567

ARAP1 14 1 5 0.0266645160157442 0.998410848301568 0.651585258628802

RGS6 8 1 1 0.0327516496754004 0.957970850346816 0.956360538099567

MTOR 15 4 17 0.788217199892873 0.995771161999986 0.0041037446505711

SEMA3D 8 1 10 0.737556972089228 0.997784051416529 0.0117574264762433

SWI5 0 2 4 1 0.509971524650284 0.0450453231390708

DIP2C 12 4 13 0.782329929348216 0.975813132897399 0.0211131571716205

COLEC12 5 2 9 0.937293353596679 0.956960131592732 0.00980209135077389

HELZ2 21 3 6 0.00620641540845805 0.995402134881724 0.857576200433114

ZRANB2 6 0 1 0.0416382254462463 1 0.887140871224982

SPTBN1 21 5 5 0.0112209554076251 0.953282989322206 0.949911687123715

KIAA2018 10 2 10 0.628378023831211 0.992060914657446 0.038811649179659

CYP7A1 5 0 6 0.646696684471791 1 0.0429142007986453

DKK1 1 1 5 0.988247552057685 0.890056051673045 0.015948402149609

TYMS 4 0 0 0.0463274983021047 1 1

ZBTB32 2 0 4 0.85760924819628 1 0.0450453231390708

EPM2AIP1 4 0 0 0.0463274983021047 1 1

DDX60 11 0 4 0.0312007424711093 1 0.596983435777183

PCDHA7 9 2 11 0.779431300729731 0.992060914657446 0.0132375397681892

COL27A1 26 4 10 0.0105913942932801 0.998549291778623 0.65750050728359

TAF5 9 0 3 0.0428170497206664 1 0.659707134958072

ATM 20 10 19 0.847861342323268 0.899179952102326 0.0311141876272494

COL5A2 7 6 11 0.978077381864585 0.662506502396728 0.0283125738357982

DTX3L 1 1 4 0.977664195795495 0.848827071050437 0.0450453231390708

GRM4 5 2 8 0.90752192082565 0.943655533200879 0.0223993563099247

OR5C1 8 1 0 0.0109413172055553 0.942028759099056 1

ANKS1B 6 1 7 0.710106395034079 0.988511036350553 0.0485352203130862

HOMEZ 0 1 4 1 0.792351146650411 0.0188689109478078

AGBL1 5 2 7 0.866192167096457 0.926551100093222 0.0485352203130862

PSG6 1 1 4 0.977664195795495 0.848827071050437 0.0450453231390708

AP4B1 3 0 6 0.875097348111752 1 0.0126373532473112

TDRD3 4 1 8 0.928573385522747 0.984085326617886 0.00735610439815661

ADGRG2 11 0 3 0.0140669844729861 1 0.766627874208378

PABPC5 4 1 7 0.892226881824287 0.977978533343354 0.0182515077914787

CFAP74 6 0 1 0.0416382254462463 1 0.887140871224982

PLCH2 5 2 7 0.866192167096457 0.926551100093222 0.0485352203130862

CEP104 5 0 7 0.737754241105828 1 0.0182515077914787

PGD 6 0 1 0.0416382254462463 1 0.887140871224982

PRAMEF20 3 0 6 0.875097348111752 1 0.0126373532473112

FHAD1 8 0 9 0.581260271056406 1 0.0162256258908508

VWA5B1 11 0 1 0.00133495879775927 1 0.976958233729919

KIF17 5 2 8 0.90752192082565 0.943655533200879 0.0223993563099247

EPHA8 3 1 6 0.920063533056499 0.957970850346816 0.0248091206279707

EPHB2 6 1 7 0.710106395034079 0.988511036350553 0.0485352203130862

LUZP1 6 0 10 0.842024768729386 1 0.00216738531492487

MAN1C1 2 1 5 0.949159299785454 0.920123595552516 0.0335780283108114

SFPQ 2 1 5 0.949159299785454 0.920123595552516 0.0335780283108114

ZFYVE9 4 1 7 0.892226881824287 0.977978533343354 0.0182515077914787

ZYG11B 4 0 8 0.892226881824287 1 0.00362872955307272

NFIA 2 0 5 0.914164058222356 1 0.015948402149609

CACHD1 6 2 9 0.887095175734593 0.967252036222198 0.0162256258908508

RAVER2 3 0 5 0.809178333533066 1 0.0335780283108114

LEPR 7 1 8 0.686045811152545 0.994031992145811 0.0349966212690756

RPE65 4 0 6 0.770176249907343 1 0.0248091206279707

MCOLN3 3 1 6 0.920063533056499 0.957970850346816 0.0248091206279707

SYDE2 12 0 4 0.0184626250071734 1 0.653890998104032

BCL10 0 0 4 1 1 0.0047530382994823

SLC25A24 1 0 4 0.957655037862292 1 0.0188689109478078

CLCC1 2 0 4 0.85760924819628 1 0.0450453231390708

RBM15 7 1 8 0.686045811152545 0.994031992145811 0.0349966212690756

CHI3L2 6 0 1 0.0416382254462463 1 0.887140871224982

RSBN1 2 0 5 0.914164058222356 1 0.015948402149609

CSDE1 5 0 9 0.866192167096457 1 0.00283038030605685

TTF2 4 1 6 0.840736001584801 0.969561061532513 0.0429142007986453

SPAG17 10 7 13 0.957952375648694 0.741715166241768 0.0289305825816211

DENND4B 11 2 11 0.612546253452738 0.995586893186678 0.0283125738357982

GON4L 9 2 11 0.779431300729731 0.992060914657446 0.0132375397681892

NES 10 1 12 0.69933388589017 0.999417866962482 0.00616228183302746

CD1D 0 0 5 1 1 0.00122407013740092

KCNJ10 2 0 4 0.85760924819628 1 0.0450453231390708

NOS1AP 2 0 4 0.85760924819628 1 0.0450453231390708

DUSP27 4 3 8 0.970254582736032 0.815372284893448 0.0223993563099247

ADCY10 6 1 7 0.710106395034079 0.988511036350553 0.0485352203130862

GPR161 7 0 1 0.0215160238576014 1 0.917693895037003

BLZF1 1 0 4 0.957655037862292 1 0.0188689109478078

SERPINC1 1 0 5 0.977664195795495 1 0.00578070485217632

BRINP2 7 1 9 0.759395689219841 0.995705739391037 0.0162256258908508

RALGPS2 1 0 4 0.957655037862292 1 0.0188689109478078

RGSL1 6 0 9 0.783532659638071 1 0.00550756920560882

PLA2G4A 9 0 2 0.0179431022115431 1 0.837233316198664

PTPRC 11 3 11 0.682407602296783 0.982528269533504 0.0393240535266177

GPR37L1 2 1 6 0.970319857406511 0.942028759099056 0.0126373532473112

ADORA1 2 1 5 0.949159299785454 0.920123595552516 0.0335780283108114

TRAF5 3 0 5 0.809178333533066 1 0.0335780283108114

PROX1 11 0 4 0.0312007424711093 1 0.596983435777183

CAPN2 3 0 6 0.875097348111752 1 0.0126373532473112

EPHX1 2 1 9 0.994478844655033 0.977978533343354 0.000513858720453461

PCNXL2 10 3 11 0.761325051916907 0.977420662701415 0.0283125738357982

SLC35F3 3 2 7 0.96900618253642 0.876968871613257 0.0182515077914787

ERO1LB 0 1 4 1 0.792351146650411 0.0188689109478078

ZP4 2 1 6 0.970319857406511 0.942028759099056 0.0126373532473112

CEP170 15 3 4 0.030356903667279 0.962736190496774 0.881661412086751

TRAPPC12 9 1 1 0.0179431022115431 0.969561061532513 0.968273260289657

ADAM17 8 0 2 0.0327516496754004 1 0.793128963052146

ATAD2B 13 1 5 0.0423335539281904 0.997784051416529 0.600319159643428

GCKR 1 0 4 0.957655037862292 1 0.0188689109478078

C2orf16 6 2 8 0.842024768729386 0.956960131592732 0.0349966212690756

ABCG8 9 1 2 0.0428170497206664 0.977978533343354 0.872738552446782

STON1-GTF2A1L 9 0 1 0.00548873408809398 1 0.956360538099567

RTN4 4 3 8 0.970254582736032 0.815372284893448 0.0223993563099247

XPO1 2 1 6 0.970319857406511 0.942028759099056 0.0126373532473112

MEIS1 5 2 7 0.866192167096457 0.926551100093222 0.0485352203130862

HK2 3 0 5 0.809178333533066 1 0.0335780283108114

PROM2 2 0 6 0.949159299785454 1 0.0053706264528876

TSGA10 4 2 7 0.928573385522747 0.90469701404897 0.0309628117503546

IL1RL2 2 0 5 0.914164058222356 1 0.015948402149609

IL18R1 4 1 7 0.892226881824287 0.977978533343354 0.0182515077914787

SLC9A4 5 0 7 0.737754241105828 1 0.0182515077914787

RGPD3 5 2 7 0.866192167096457 0.926551100093222 0.0485352203130862

ZC3H6 6 1 7 0.710106395034079 0.988511036350553 0.0485352203130862

DPP10 7 3 10 0.904466197661763 0.939561585206236 0.0182961548166117

SCTR 2 0 4 0.85760924819628 1 0.0450453231390708

ERCC3 3 0 5 0.809178333533066 1 0.0335780283108114

MYO7B 15 0 6 0.0164664929781717 1 0.501165529800654

RBM43 0 0 4 1 1 0.0047530382994823

CCDC148 2 0 5 0.914164058222356 1 0.015948402149609

PKP4 4 1 7 0.892226881824287 0.977978533343354 0.0182515077914787

SLC38A11 1 1 4 0.977664195795495 0.848827071050437 0.0450453231390708

SCN3A 20 2 10 0.0445525762336056 0.999617079410581 0.329556680075644

TTC21B 6 1 7 0.710106395034079 0.988511036350553 0.0485352203130862

CWC22 3 0 7 0.920063533056499 1 0.00452284166020484

ZNF142 8 0 11 0.737556972089228 1 0.00302248403681973

UGT1A9 1 0 4 0.957655037862292 1 0.0188689109478078

CNTN4 8 3 10 0.848761740459206 0.952433770201283 0.0271870539926272

IRAK2 3 1 6 0.920063533056499 0.957970850346816 0.0248091206279707

C3orf20 7 0 7 0.50323080825299 1 0.0485352203130862

EFHB 4 3 7 0.953525872893264 0.773572497783455 0.0485352203130862

THRB 9 0 3 0.0428170497206664 1 0.659707134958072

TOP2B 10 3 13 0.857429223244514 0.98653069146967 0.00691381170407501

SLC4A7 8 0 10 0.664761248320516 1 0.00716008835023087

DLEC1 14 2 4 0.0266645160157442 0.985874910600787 0.826108148791781

ENTPD3 2 0 5 0.914164058222356 1 0.015948402149609

ZNF662 2 0 4 0.85760924819628 1 0.0450453231390708

ZNF197 2 1 5 0.949159299785454 0.920123595552516 0.0335780283108114

MAP4 5 2 7 0.866192167096457 0.926551100093222 0.0485352203130862

ITIH1 5 2 7 0.866192167096457 0.926551100093222 0.0485352203130862

MITF 4 1 6 0.840736001584801 0.969561061532513 0.0429142007986453

DHFRL1 2 0 4 0.85760924819628 1 0.0450453231390708

IMPG2 5 2 7 0.866192167096457 0.926551100093222 0.0485352203130862

MORC1 10 0 2 0.00965178637516871 1 0.872738552446782

ZBED2 2 0 4 0.85760924819628 1 0.0450453231390708

CD200 0 0 5 1 1 0.00122407013740092

ADCY5 16 2 4 0.00998430132325486 0.992060914657446 0.881661412086751

CCDC37 6 0 7 0.621445429270755 1 0.0309628117503546

RASA2 3 2 7 0.96900618253642 0.876968871613257 0.0182515077914787

SLC9A9 8 1 1 0.0327516496754004 0.957970850346816 0.956360538099567

TSC22D2 1 0 6 0.988247552057685 1 0.00171362593320324

SIAH2 2 0 4 0.85760924819628 1 0.0450453231390708

GPR171 2 0 4 0.85760924819628 1 0.0450453231390708

GMPS 3 0 5 0.809178333533066 1 0.0335780283108114

GFM1 3 1 6 0.920063533056499 0.957970850346816 0.0248091206279707

LRRIQ4 2 1 5 0.949159299785454 0.920123595552516 0.0335780283108114

PRKCI 2 2 6 0.982883938494014 0.79854993786922 0.0248091206279707

TNIK 14 2 5 0.0470605620908893 0.98939503064284 0.698591423887599

KNG1 3 0 5 0.809178333533066 1 0.0335780283108114

ATP13A5 6 1 9 0.842024768729386 0.994031992145811 0.00980209135077389

WHSC1 6 1 9 0.842024768729386 0.994031992145811 0.00980209135077389

OTOP1 4 0 6 0.770176249907343 1 0.0248091206279707

CCDC96 2 0 5 0.914164058222356 1 0.015948402149609

AFAP1 2 1 6 0.970319857406511 0.942028759099056 0.0126373532473112

SEL1L3 7 2 9 0.819617606155775 0.975173805854432 0.0252911634314708

NWD2 10 1 13 0.761325051916907 0.999584431039691 0.00275245738017136

TEC 2 0 4 0.85760924819628 1 0.0450453231390708

FIP1L1 3 0 6 0.875097348111752 1 0.0126373532473112

TMPRSS11D 2 2 7 0.990233400968598 0.842068738489845 0.0097215538190752

UGT2B15 5 0 6 0.646696684471791 1 0.0429142007986453

FGF5 1 0 4 0.957655037862292 1 0.0188689109478078

THAP9 2 2 6 0.982883938494014 0.79854993786922 0.0248091206279707

HERC5 2 3 7 0.994478844655033 0.667568072872785 0.0182515077914787

STPG2 3 1 6 0.920063533056499 0.957970850346816 0.0248091206279707

DNAJB14 2 0 4 0.85760924819628 1 0.0450453231390708

BANK1 4 1 6 0.840736001584801 0.969561061532513 0.0429142007986453

SLC39A8 1 0 6 0.988247552057685 1 0.00171362593320324

ENPEP 6 1 7 0.710106395034079 0.988511036350553 0.0485352203130862

PRSS12 4 1 7 0.892226881824287 0.977978533343354 0.0182515077914787

SYNPO2 12 0 4 0.0184626250071734 1 0.653890998104032

PDE5A 1 0 7 0.993831588096575 1 0.000494625759975115

BBS12 2 1 6 0.970319857406511 0.942028759099056 0.0126373532473112

GAB1 2 1 5 0.949159299785454 0.920123595552516 0.0335780283108114

PLRG1 2 0 4 0.85760924819628 1 0.0450453231390708

TDO2 1 1 5 0.988247552057685 0.890056051673045 0.015948402149609

CPE 2 1 5 0.949159299785454 0.920123595552516 0.0335780283108114

LRP2BP 2 0 6 0.949159299785454 1 0.0053706264528876

FASTKD3 6 2 8 0.842024768729386 0.956960131592732 0.0349966212690756

CAPSL 1 1 4 0.977664195795495 0.848827071050437 0.0450453231390708

UGT3A2 5 0 8 0.81044724850275 1 0.00735610439815661

ADAMTS6 3 0 6 0.875097348111752 1 0.0126373532473112

ENC1 7 3 9 0.867486309361261 0.923580742095784 0.0374715266911755

MSH3 2 1 6 0.970319857406511 0.942028759099056 0.0126373532473112

XRCC4 2 1 5 0.949159299785454 0.920123595552516 0.0335780283108114

TTC37 6 0 10 0.842024768729386 1 0.00216738531492487

FBXL17 2 0 6 0.949159299785454 1 0.0053706264528876

WDR36 4 1 7 0.892226881824287 0.977978533343354 0.0182515077914787

DDX46 6 0 1 0.0416382254462463 1 0.887140871224982

KLHL3 3 0 5 0.809178333533066 1 0.0335780283108114

MATR3 7 0 7 0.50323080825299 1 0.0485352203130862

PCDHB16 5 0 6 0.646696684471791 1 0.0429142007986453

PCDHGA10 4 2 7 0.928573385522747 0.90469701404897 0.0309628117503546

PDE6A 5 1 7 0.81044724850275 0.984085326617886 0.0309628117503546

ZNF879 3 1 6 0.920063533056499 0.957970850346816 0.0248091206279707

FLT4 10 4 11 0.813936684996989 0.939964879266099 0.0393240535266177

HIVEP1 14 2 4 0.0266645160157442 0.985874910600787 0.826108148791781

FAM65B 5 0 8 0.81044724850275 1 0.00735610439815661

LRRC16A 6 2 11 0.945455953368343 0.981243578061332 0.00302248403681973

SLC17A1 1 1 6 0.993831588096575 0.920123595552516 0.0053706264528876

HIST1H2BL 0 0 4 1 1 0.0047530382994823

OR2B6 1 1 6 0.993831588096575 0.920123595552516 0.0053706264528876

ZNF311 1 0 6 0.988247552057685 1 0.00171362593320324

XXbac-BPG32J3.19 2 0 4 0.85760924819628 1 0.0450453231390708

TAP2 4 2 7 0.928573385522747 0.90469701404897 0.0309628117503546

TCP11 2 0 4 0.85760924819628 1 0.0450453231390708

PTK7 3 2 8 0.981136021640521 0.90469701404897 0.00735610439815661

HSP90AB1 1 0 4 0.957655037862292 1 0.0188689109478078

BMP5 2 1 5 0.949159299785454 0.920123595552516 0.0335780283108114

RIPPLY2 0 0 4 1 1 0.0047530382994823

CASP8AP2 7 1 11 0.867486309361261 0.997784051416529 0.00302248403681973

C6orf203 0 0 4 1 1 0.0047530382994823

HDAC2 6 0 1 0.0416382254462463 1 0.887140871224982

L3MBTL3 4 1 6 0.840736001584801 0.969561061532513 0.0429142007986453

ADGRG6 6 0 7 0.621445429270755 1 0.0309628117503546

NOX3 3 1 6 0.920063533056499 0.957970850346816 0.0248091206279707

TULP4 18 4 4 0.0138211211596331 0.951843034689561 0.948723519123803

QKI 2 0 4 0.85760924819628 1 0.0450453231390708

INTS1 6 4 10 0.963022991528306 0.833022631136556 0.0182961548166117

RBAK 3 2 6 0.949811357358387 0.842068738489845 0.0429142007986453

AHR 4 1 6 0.840736001584801 0.969561061532513 0.0429142007986453

RAMP3 0 1 4 1 0.792351146650411 0.0188689109478078

SUN3 2 0 4 0.85760924819628 1 0.0450453231390708

FIGNL1 2 1 5 0.949159299785454 0.920123595552516 0.0335780283108114

ZNF727 1 1 4 0.977664195795495 0.848827071050437 0.0450453231390708

SEMA3E 5 0 6 0.646696684471791 1 0.0429142007986453

PPP1R9A 15 2 3 0.00803043203609599 0.985874910600787 0.936333132052609

PON1 3 0 5 0.809178333533066 1 0.0335780283108114

COPS6 2 1 6 0.970319857406511 0.942028759099056 0.0126373532473112

FBXO24 8 0 2 0.0327516496754004 1 0.793128963052146

CFTR 4 3 7 0.953525872893264 0.773572497783455 0.0485352203130862

WASL 6 0 7 0.621445429270755 1 0.0309628117503546

SND1 8 0 8 0.489294238525221 1 0.0349966212690756

PLXNA4 17 3 15 0.472388122476334 0.998879252432728 0.0211622556560952

AKR1B1 2 0 5 0.914164058222356 1 0.015948402149609

AKR1B15 1 0 4 0.957655037862292 1 0.0188689109478078

NUP205 16 2 13 0.345579509864685 0.999476232181452 0.0386927559058104

TCAF2 1 0 4 0.957655037862292 1 0.0188689109478078

GIMAP7 2 1 5 0.949159299785454 0.920123595552516 0.0335780283108114

ABCB8 4 1 7 0.892226881824287 0.977978533343354 0.0182515077914787

GALNT11 2 0 5 0.914164058222356 1 0.015948402149609

ZNF596 2 0 4 0.85760924819628 1 0.0450453231390708

SOX7 2 2 6 0.982883938494014 0.79854993786922 0.0248091206279707

DLC1 16 2 13 0.345579509864685 0.999476232181452 0.0386927559058104

MSR1 1 1 4 0.977664195795495 0.848827071050437 0.0450453231390708

EBF2 2 0 6 0.949159299785454 1 0.0053706264528876

KIF13B 9 1 10 0.645678369304004 0.998410848301568 0.0182961548166117

BRF2 6 0 1 0.0416382254462463 1 0.887140871224982

ANK1 12 2 11 0.523631809469409 0.996722425678259 0.0393240535266177

PRKDC 16 7 16 0.81816869489837 0.939918834926978 0.0274315720947039

IMPAD1 1 0 5 0.977664195795495 1 0.00578070485217632

TOX 3 1 7 0.949811357358387 0.969561061532513 0.0097215538190752

ARFGEF1 12 3 13 0.728377233320583 0.992078693950593 0.0150029855867926

HRSP12 1 1 4 0.977664195795495 0.848827071050437 0.0450453231390708

SNX31 2 1 5 0.949159299785454 0.920123595552516 0.0335780283108114

RNF139 3 0 5 0.809178333533066 1 0.0335780283108114

DENND3 5 2 7 0.866192167096457 0.926551100093222 0.0485352203130862

SLC1A1 2 0 7 0.970319857406511 1 0.0017372630556758

AK3 0 0 4 1 1 0.0047530382994823

AQP7 1 0 5 0.977664195795495 1 0.00578070485217632

ZCCHC7 2 0 4 0.85760924819628 1 0.0450453231390708

FBXO10 7 2 9 0.819617606155775 0.975173805854432 0.0252911634314708

SPATA31E1 10 0 9 0.37934729540708 1 0.0374715266911755

NOL8 9 0 3 0.0428170497206664 1 0.659707134958072

COL15A1 6 2 9 0.887095175734593 0.967252036222198 0.0162256258908508

FKTN 0 2 4 1 0.509971524650284 0.0450453231390708

PAPPA 11 4 14 0.8795118041806 0.975813132897399 0.00748277440546585

OR1L3 2 0 4 0.85760924819628 1 0.0450453231390708

FAM129B 8 0 2 0.0327516496754004 1 0.793128963052146

SLC2A6 2 0 5 0.914164058222356 1 0.015948402149609

SNAPC4 4 1 7 0.892226881824287 0.977978533343354 0.0182515077914787

IL2RA 0 0 4 1 1 0.0047530382994823

SKIDA1 2 0 7 0.970319857406511 1 0.0017372630556758

ENKUR 1 1 4 0.977664195795495 0.848827071050437 0.0450453231390708

ZNF33A 2 2 6 0.982883938494014 0.79854993786922 0.0248091206279707

AGAP9 3 0 5 0.809178333533066 1 0.0335780283108114

EGR2 5 2 8 0.90752192082565 0.943655533200879 0.0223993563099247

TBATA 6 0 1 0.0416382254462463 1 0.887140871224982

OIT3 1 0 4 0.957655037862292 1 0.0188689109478078

NUDT13 0 2 4 1 0.509971524650284 0.0450453231390708

CFAP70 5 0 6 0.646696684471791 1 0.0429142007986453

ANXA7 0 0 4 1 1 0.0047530382994823

ADK 1 1 4 0.977664195795495 0.848827071050437 0.0450453231390708

ANXA11 1 1 6 0.993831588096575 0.920123595552516 0.0053706264528876

PTEN 11 1 14 0.744348623800949 0.999788959039115 0.0019910439599851

IFIT1 0 1 6 1 0.890056051673045 0.00171362593320324

EXOC6 14 0 4 0.00611185694213879 1 0.751017378945341

CWF19L1 2 0 4 0.85760924819628 1 0.0450453231390708

C10orf90 5 1 7 0.81044724850275 0.984085326617886 0.0309628117503546

DOCK1 7 3 9 0.867486309361261 0.923580742095784 0.0374715266911755

CARS 3 0 6 0.875097348111752 1 0.0126373532473112

OR52A5 9 1 1 0.0179431022115431 0.969561061532513 0.968273260289657

OR56A5 2 0 4 0.85760924819628 1 0.0450453231390708

SMPD1 2 1 8 0.990233400968598 0.969561061532513 0.00155214899799321

COPB1 1 0 5 0.977664195795495 1 0.00578070485217632

E2F8 3 0 6 0.875097348111752 1 0.0126373532473112

NELL1 4 1 9 0.953525872893264 0.988511036350553 0.00283038030605685

PDHX 1 0 4 0.957655037862292 1 0.0188689109478078

CREB3L1 2 2 6 0.982883938494014 0.79854993786922 0.0248091206279707

MTCH2 1 1 4 0.977664195795495 0.848827071050437 0.0450453231390708

OR8H3 1 0 4 0.957655037862292 1 0.0188689109478078

PRG2 1 0 4 0.957655037862292 1 0.0188689109478078

OR5B2 2 0 4 0.85760924819628 1 0.0450453231390708

NAALADL1 12 0 5 0.0370537811471727 1 0.486876169292091

DRAP1 1 1 4 0.977664195795495 0.848827071050437 0.0450453231390708

KLC2 5 0 7 0.737754241105828 1 0.0182515077914787

LRTOMT 0 2 4 1 0.509971524650284 0.0450453231390708

RNF169 3 0 5 0.809178333533066 1 0.0335780283108114

CCDC81 2 0 4 0.85760924819628 1 0.0450453231390708

GPR83 1 0 4 0.957655037862292 1 0.0188689109478078

JAM3 2 0 7 0.970319857406511 1 0.0017372630556758

NCAPD3 6 5 10 0.975304817634412 0.708472793714122 0.0271870539926272

NDUFA9 2 0 5 0.914164058222356 1 0.015948402149609

KCNA6 10 1 1 0.00965178637516871 0.977978533343354 0.976958233729919

NCAPD2 5 2 7 0.866192167096457 0.926551100093222 0.0485352203130862

CHD4 13 5 14 0.814519027447064 0.962206633405786 0.0212679208166849

ST8SIA1 2 1 6 0.970319857406511 0.942028759099056 0.0126373532473112

ITPR2 18 2 8 0.0396841904048422 0.998676500832996 0.476737257630097

KMT2D 35 5 18 0.0161713863910841 0.999955581399479 0.245747058477838

AVPR1A 5 0 7 0.737754241105828 1 0.0182515077914787

CAND1 6 2 8 0.842024768729386 0.956960131592732 0.0349966212690756

SYT1 2 0 4 0.85760924819628 1 0.0450453231390708

MGAT4C 6 1 7 0.710106395034079 0.988511036350553 0.0485352203130862

EEA1 5 0 8 0.81044724850275 1 0.00735610439815661

STAB2 8 4 13 0.959355373009428 0.939964879266099 0.00445242783203005

KIAA1033 8 1 1 0.0327516496754004 0.957970850346816 0.956360538099567

POLR3B 10 0 2 0.00965178637516871 1 0.872738552446782

SELPLG 0 2 4 1 0.509971524650284 0.0450453231390708

RAD9B 2 0 4 0.85760924819628 1 0.0450453231390708

P2RX4 1 0 4 0.957655037862292 1 0.0188689109478078

TMEM132D 15 5 15 0.74003194308307 0.980501401443531 0.0211622556560952

RIMBP2 11 3 11 0.682407602296783 0.982528269533504 0.0393240535266177

POLE 18 1 8 0.0242321024235943 0.99984987173628 0.42911464415598

ZNF84 3 0 5 0.809178333533066 1 0.0335780283108114

ATP12A 8 2 9 0.737556972089228 0.981243578061332 0.0374715266911755

HSPH1 2 0 5 0.914164058222356 1 0.015948402149609

B3GALTL 1 0 7 0.993831588096575 1 0.000494625759975115

RXFP2 4 1 8 0.928573385522747 0.984085326617886 0.00735610439815661

NHLRC3 2 2 6 0.982883938494014 0.79854993786922 0.0248091206279707

NUFIP1 2 0 5 0.914164058222356 1 0.015948402149609

ZC3H13 14 3 14 0.638118864423434 0.996513180514699 0.0154137272096665

KIAA0226L 2 0 4 0.85760924819628 1 0.0450453231390708

WDFY2 2 0 4 0.85760924819628 1 0.0450453231390708

LMO7 9 0 9 0.476884049476269 1 0.0252911634314708

EDNRB 6 0 7 0.621445429270755 1 0.0309628117503546

FAM155A 2 0 4 0.85760924819628 1 0.0450453231390708

ING1 2 2 7 0.990233400968598 0.842068738489845 0.0097215538190752

MCF2L 6 0 8 0.710106395034079 1 0.0133904031876229

OR4K5 2 0 6 0.949159299785454 1 0.0053706264528876

ADCY4 1 1 9 0.999121522838445 0.969561061532513 0.000165179149637874

BRMS1L 0 0 5 1 1 0.00122407013740092

FSCB 9 1 2 0.0428170497206664 0.977978533343354 0.872738552446782

FAM179B 9 2 11 0.779431300729731 0.992060914657446 0.0132375397681892

MAP4K5 3 0 5 0.809178333533066 1 0.0335780283108114

PTGER2 0 0 4 1 1 0.0047530382994823

ARID4A 3 1 7 0.949811357358387 0.969561061532513 0.0097215538190752

KIAA0586 5 2 9 0.937293353596679 0.956960131592732 0.00980209135077389

YLPM1 10 2 13 0.813936684996989 0.996722425678259 0.00445242783203005

ZC2HC1C 6 0 1 0.0416382254462463 1 0.887140871224982

NRDE2 4 1 6 0.840736001584801 0.969561061532513 0.0429142007986453

SERPINA11 2 0 6 0.949159299785454 1 0.0053706264528876

CLMN 5 0 6 0.646696684471791 1 0.0429142007986453

EML1 5 1 8 0.866192167096457 0.988511036350553 0.0133904031876229

TDRD9 2 0 5 0.914164058222356 1 0.015948402149609

ATP10A 18 6 16 0.648471192752373 0.98081534031545 0.0354650747217132

MTMR10 1 1 4 0.977664195795495 0.848827071050437 0.0450453231390708

RPAP1 3 0 6 0.875097348111752 1 0.0126373532473112

MAPKBP1 3 1 10 0.988666062207955 0.988511036350553 0.00044842025758331

STRC 2 0 5 0.914164058222356 1 0.015948402149609

SPG11 14 2 5 0.0470605620908893 0.98939503064284 0.698591423887599

MYEF2 3 0 5 0.809178333533066 1 0.0335780283108114

MNS1 1 0 5 0.977664195795495 1 0.00578070485217632

USP3 2 1 8 0.990233400968598 0.969561061532513 0.00155214899799321

MAP2K1 3 0 7 0.920063533056499 1 0.00452284166020484

LRRC49 5 2 7 0.866192167096457 0.926551100093222 0.0485352203130862

SIN3A 9 1 9 0.564667128138538 0.997784051416529 0.0374715266911755

CTSH 0 1 5 1 0.848827071050437 0.00578070485217632

SAXO2 6 0 1 0.0416382254462463 1 0.887140871224982

SYNM 5 0 9 0.866192167096457 1 0.00283038030605685

MSLN 3 1 6 0.920063533056499 0.957970850346816 0.0248091206279707

TELO2 9 1 2 0.0428170497206664 0.977978533343354 0.872738552446782

ZNF205 2 0 6 0.949159299785454 1 0.0053706264528876

UMOD 10 0 2 0.00965178637516871 1 0.872738552446782

XPO6 5 1 9 0.90752192082565 0.991715000861244 0.00550756920560882

PHKG2 1 0 4 0.957655037862292 1 0.0188689109478078

CES1 3 1 6 0.920063533056499 0.957970850346816 0.0248091206279707

CES5A 3 2 6 0.949811357358387 0.842068738489845 0.0429142007986453

NLRC5 14 2 12 0.428574009876498 0.998676500832996 0.0392305264057035

KCTD19 3 3 8 0.988666062207955 0.773572497783455 0.0133904031876229

NFAT5 1 1 4 0.977664195795495 0.848827071050437 0.0450453231390708

GLG1 12 1 3 0.0184626250071734 0.994031992145811 0.844842365134364

GAN 3 0 5 0.809178333533066 1 0.0335780283108114

METTL16 0 1 4 1 0.792351146650411 0.0188689109478078

GLTPD2 0 0 4 1 1 0.0047530382994823

PITPNM3 6 2 9 0.887095175734593 0.967252036222198 0.0162256258908508

NEURL4 7 1 9 0.759395689219841 0.995705739391037 0.0162256258908508

KRBA2 2 0 4 0.85760924819628 1 0.0450453231390708

MYH8 11 2 15 0.842649713726937 0.998676500832996 0.00143611436652662

NOS2 6 1 8 0.783532659638071 0.991715000861244 0.0223993563099247

EFCAB5 9 1 9 0.564667128138538 0.997784051416529 0.0374715266911755

PSMD11 6 0 1 0.0416382254462463 1 0.887140871224982

SLFN14 6 1 7 0.710106395034079 0.988511036350553 0.0485352203130862

CDK12 10 3 11 0.761325051916907 0.977420662701415 0.0283125738357982

GRB7 2 0 4 0.85760924819628 1 0.0450453231390708

DNAJC7 2 0 4 0.85760924819628 1 0.0450453231390708

EFCAB13 6 1 7 0.710106395034079 0.988511036350553 0.0485352203130862

SAMD14 3 0 6 0.875097348111752 1 0.0126373532473112

MYCBPAP 2 0 6 0.949159299785454 1 0.0053706264528876

BZRAP1 8 1 9 0.664761248320516 0.996913500187308 0.0252911634314708

HSF5 2 0 5 0.914164058222356 1 0.015948402149609

SMG8 7 0 1 0.0215160238576014 1 0.917693895037003

GRIN2C 2 1 10 0.996904812475854 0.984085326617886 0.000164853585145983

CEP131 11 3 1 0.0312007424711093 0.815372284893448 0.991229551692957

EMILIN2 2 0 4 0.85760924819628 1 0.0450453231390708

LPIN2 0 0 6 1 1 0.00031274319444584

ZBTB14 1 1 5 0.988247552057685 0.890056051673045 0.015948402149609

ZNF519 2 0 4 0.85760924819628 1 0.0450453231390708

POTEC 12 0 3 0.00783652701912318 1 0.809038983816177

CABYR 1 1 5 0.988247552057685 0.890056051673045 0.015948402149609

ZNF24 2 0 4 0.85760924819628 1 0.0450453231390708

KIAA1328 0 0 4 1 1 0.0047530382994823

KATNAL2 1 0 4 0.957655037862292 1 0.0188689109478078

POLI 2 1 6 0.970319857406511 0.942028759099056 0.0126373532473112

CCDC102B 4 0 6 0.770176249907343 1 0.0248091206279707

APC2 4 0 6 0.770176249907343 1 0.0248091206279707

GNA11 2 0 4 0.85760924819628 1 0.0450453231390708

UBXN6 1 0 6 0.988247552057685 1 0.00171362593320324

MLLT1 8 0 2 0.0327516496754004 1 0.793128963052146

C3 9 1 11 0.717636778362154 0.998861639184504 0.0085161657229947

VAV1 3 1 7 0.949811357358387 0.969561061532513 0.0097215538190752

ZNF121 2 0 4 0.85760924819628 1 0.0450453231390708

ZNF441 2 0 4 0.85760924819628 1 0.0450453231390708

SYCE2 1 0 4 0.957655037862292 1 0.0188689109478078

MAP1S 8 0 8 0.489294238525221 1 0.0349966212690756

KIRREL2 5 0 8 0.81044724850275 1 0.00735610439815661

ZFP30 3 1 7 0.949811357358387 0.969561061532513 0.0097215538190752

ZNF546 4 0 8 0.892226881824287 1 0.00362872955307272

CYP2A7 4 0 6 0.770176249907343 1 0.0248091206279707

ZNF221 3 0 6 0.875097348111752 1 0.0126373532473112

LIG1 4 1 6 0.840736001584801 0.969561061532513 0.0429142007986453

KDELR1 2 0 5 0.914164058222356 1 0.015948402149609

CEACAM18 2 0 4 0.85760924819628 1 0.0450453231390708

ZNF432 2 1 5 0.949159299785454 0.920123595552516 0.0335780283108114

ZNF480 3 0 7 0.920063533056499 1 0.00452284166020484

ZNF83 5 0 6 0.646696684471791 1 0.0429142007986453

ZNF814 5 2 7 0.866192167096457 0.926551100093222 0.0485352203130862

TBC1D20 1 0 4 0.957655037862292 1 0.0188689109478078

CSNK2A1 1 0 5 0.977664195795495 1 0.00578070485217632

ATRN 5 0 7 0.737754241105828 1 0.0182515077914787

MACROD2 2 0 5 0.914164058222356 1 0.015948402149609

RBM39 0 0 4 1 1 0.0047530382994823

FAM83D 1 1 5 0.988247552057685 0.890056051673045 0.015948402149609

TOMM34 0 0 4 1 1 0.0047530382994823

PREX1 14 2 4 0.0266645160157442 0.985874910600787 0.826108148791781

TPTE 9 1 10 0.645678369304004 0.998410848301568 0.0182961548166117

RWDD2B 1 1 4 0.977664195795495 0.848827071050437 0.0450453231390708

PRDM15 11 0 4 0.0312007424711093 1 0.596983435777183

BCR 5 0 7 0.737754241105828 1 0.0182515077914787

EMID1 2 1 5 0.949159299785454 0.920123595552516 0.0335780283108114

C22orf23 1 0 5 0.977664195795495 1 0.00578070485217632

MKL1 4 2 11 0.988328550831695 0.967252036222198 0.000819957025878434

KIAA1644 2 0 7 0.970319857406511 1 0.0017372630556758

MOV10L1 3 3 9 0.993268932075936 0.815372284893448 0.00550756920560882

MSL3 2 1 6 0.970319857406511 0.942028759099056 0.0126373532473112

ZNF41 1 0 4 0.957655037862292 1 0.0188689109478078

PIM2 0 1 4 1 0.792351146650411 0.0188689109478078

CCNB3 3 0 6 0.875097348111752 1 0.0126373532473112

XAGE2 0 0 4 1 1 0.0047530382994823

EFNB1 1 1 4 0.977664195795495 0.848827071050437 0.0450453231390708

NLGN3 2 3 8 0.996904812475854 0.724464088216835 0.00735610439815661

RGAG4 4 1 7 0.892226881824287 0.977978533343354 0.0182515077914787

KLHL4 15 1 5 0.0164664929781717 0.998861639184504 0.698591423887599

GPRASP1 5 2 10 0.958209261039215 0.967252036222198 0.00409228287070551

IL13RA2 2 0 4 0.85760924819628 1 0.0450453231390708

MCF2 5 4 13 0.995560041953407 0.887185108329836 0.000909507539070351

SLITRK2 8 2 9 0.737556972089228 0.981243578061332 0.0374715266911755

FLNA 22 3 8 0.012463832952437 0.998012173298243 0.691899060276489

PLXNA3 7 3 10 0.904466197661763 0.939561585206236 0.0182961548166117

PADI6 4 0 6 0.770176249907343 1 0.0248091206279707

ZNF644 6 1 7 0.710106395034079 0.988511036350553 0.0485352203130862

PRPF38B 3 0 5 0.809178333533066 1 0.0335780283108114

CFH 9 3 12 0.87268096242383 0.977420662701415 0.00957210628588356

LRRN2 1 1 4 0.977664195795495 0.848827071050437 0.0450453231390708

MARK1 5 1 8 0.866192167096457 0.988511036350553 0.0133904031876229

UQCRC1 1 1 4 0.977664195795495 0.848827071050437 0.0450453231390708

CDHR4 3 1 6 0.920063533056499 0.957970850346816 0.0248091206279707

SPICE1 4 0 6 0.770176249907343 1 0.0248091206279707

WDR19 6 1 7 0.710106395034079 0.988511036350553 0.0485352203130862

SHROOM3 13 2 4 0.0423335539281904 0.981243578061332 0.791203570759185

DCLK2 4 0 7 0.840736001584801 1 0.0097215538190752

ANXA10 1 1 4 0.977664195795495 0.848827071050437 0.0450453231390708

CHD1 11 2 11 0.612546253452738 0.995586893186678 0.0283125738357982

GMPR 2 0 4 0.85760924819628 1 0.0450453231390708

CCDC28A 3 0 5 0.809178333533066 1 0.0335780283108114

HAS2 1 0 5 0.977664195795495 1 0.00578070485217632

RFX3 0 0 4 1 1 0.0047530382994823

RGS3 5 2 8 0.90752192082565 0.943655533200879 0.0223993563099247

BICC1 6 3 10 0.945455953368343 0.923580742095784 0.0117574264762433

WNT11 4 0 6 0.770176249907343 1 0.0248091206279707

SC5D 2 0 4 0.85760924819628 1 0.0450453231390708

GYS2 5 0 6 0.646696684471791 1 0.0429142007986453

FAM186A 9 1 9 0.564667128138538 0.997784051416529 0.0374715266911755

CSRNP2 1 0 4 0.957655037862292 1 0.0188689109478078

OR6C4 1 1 5 0.988247552057685 0.890056051673045 0.015948402149609

GDF11 0 0 4 1 1 0.0047530382994823

STAT2 2 1 6 0.970319857406511 0.942028759099056 0.0126373532473112

TPCN1 4 0 6 0.770176249907343 1 0.0248091206279707

ATP8B4 8 0 8 0.489294238525221 1 0.0349966212690756

PEAK1 5 2 7 0.866192167096457 0.926551100093222 0.0485352203130862

PDILT 6 0 7 0.621445429270755 1 0.0309628117503546

SLC12A3 3 2 6 0.949811357358387 0.842068738489845 0.0429142007986453

RANBP10 4 1 7 0.892226881824287 0.977978533343354 0.0182515077914787

ABR 7 0 9 0.686045811152545 1 0.00980209135077389

OR1E2 1 0 4 0.957655037862292 1 0.0188689109478078

MPP2 3 2 6 0.949811357358387 0.842068738489845 0.0429142007986453

NUP85 2 0 4 0.85760924819628 1 0.0450453231390708

TPM4 0 0 4 1 1 0.0047530382994823

FAM187B 1 0 4 0.957655037862292 1 0.0188689109478078

APLP1 6 3 9 0.920833960524002 0.903877879164331 0.0252911634314708

STRN4 6 0 1 0.0416382254462463 1 0.887140871224982

PLEKHA4 4 1 6 0.840736001584801 0.969561061532513 0.0429142007986453

EPN1 5 2 7 0.866192167096457 0.926551100093222 0.0485352203130862

LZTR1 8 2 9 0.737556972089228 0.981243578061332 0.0374715266911755

STARD8 6 1 7 0.710106395034079 0.988511036350553 0.0485352203130862

ALPP 9 0 0 0.000926368622463118 1 1

DECR1 4 0 0 0.0463274983021047 1 1

CCDC62 8 1 1 0.0327516496754004 0.957970850346816 0.956360538099567

MAP7D1 14 3 3 0.0266645160157442 0.939561585206236 0.936333132052609

RNPEP 4 0 0 0.0463274983021047 1 1

HHAT 7 0 1 0.0215160238576014 1 0.917693895037003

ACTA1 9 1 2 0.0428170497206664 0.977978533343354 0.872738552446782

CACNB4 6 0 1 0.0416382254462463 1 0.887140871224982

ANKMY1 9 1 2 0.0428170497206664 0.977978533343354 0.872738552446782

LIMD1 2 0 5 0.914164058222356 1 0.015948402149609

CCDC66 10 0 2 0.00965178637516871 1 0.872738552446782

ACAD11 9 1 1 0.0179431022115431 0.969561061532513 0.968273260289657

TM4SF4 5 0 0 0.0213233416842564 1 1

FNDC3B 6 1 9 0.842024768729386 0.994031992145811 0.00980209135077389

ATP13A3 6 1 9 0.842024768729386 0.994031992145811 0.00980209135077389

LMLN 4 0 0 0.0463274983021047 1 1

TCERG1 9 1 12 0.779431300729731 0.999185483209602 0.00379479167780028

XPO5 8 1 1 0.0327516496754004 0.957970850346816 0.956360538099567

ARFGEF3 13 1 3 0.0107163099488403 0.995705739391037 0.874748548477439

LTV1 5 0 0 0.0213233416842564 1 1

FBXL18 10 0 3 0.0247944696393595 1 0.716992894282767

TBL2 8 0 1 0.0109413172055553 1 0.940037380594825

CADPS2 7 1 8 0.686045811152545 0.994031992145811 0.0349966212690756

ZNF786 6 1 0 0.0416382254462463 0.890056051673045 1

TRIB1 5 0 0 0.0213233416842564 1 1

ZNF16 9 1 1 0.0179431022115431 0.969561061532513 0.968273260289657

TJP2 12 1 4 0.0370537811471727 0.995705739391037 0.705280176200014

AKR1C1 4 0 0 0.0463274983021047 1 1

NEBL 6 0 0 0.00978296170678797 1 1

KIAA1217 19 3 5 0.00861563052765254 0.989653086230853 0.890187128278008

MYOZ1 4 0 0 0.0463274983021047 1 1

NOLC1 8 0 0 0.00203914068725113 1 1

EIF3M 4 0 0 0.0463274983021047 1 1

OR8H2 6 1 0 0.0416382254462463 0.890056051673045 1

DTX4 6 0 7 0.621445429270755 1 0.0309628117503546

RDX 8 0 2 0.0327516496754004 1 0.793128963052146

TXNRD1 6 1 0 0.0416382254462463 0.890056051673045 1

CHST11 2 0 4 0.85760924819628 1 0.0450453231390708

TNFRSF19 8 0 2 0.0327516496754004 1 0.793128963052146

GNB5 7 0 0 0.00447375108354491 1 1

PRR25 4 0 0 0.0463274983021047 1 1

MKL2 9 0 2 0.0179431022115431 1 0.837233316198664

ZNF688 1 0 4 0.957655037862292 1 0.0188689109478078

LRRC36 10 2 1 0.0247944696393595 0.90469701404897 0.983283424470725

JPH3 15 4 3 0.030356903667279 0.887185108329836 0.960446763764443

RNMTL1 5 0 0 0.0213233416842564 1 1

OR1D2 4 0 0 0.0463274983021047 1 1

FLCN 8 0 1 0.0109413172055553 1 0.940037380594825

SPECC1 13 1 5 0.0423335539281904 0.997784051416529 0.600319159643428

RAB11FIP4 7 0 1 0.0215160238576014 1 0.917693895037003

LIG3 13 3 3 0.0423335539281904 0.923580742095784 0.919799384563485

SLFN12 4 0 0 0.0463274983021047 1 1

ACLY 12 2 2 0.0184626250071734 0.956960131592732 0.954809893468796

SPATA20 8 0 1 0.0109413172055553 1 0.940037380594825

DDX42 8 0 2 0.0327516496754004 1 0.793128963052146

HDGFRP2 9 1 2 0.0428170497206664 0.977978533343354 0.872738552446782

HNRNPUL1 8 0 2 0.0327516496754004 1 0.793128963052146

ZNF155 6 0 0 0.00978296170678797 1 1

SOGA1 12 3 2 0.0370537811471727 0.879770846871731 0.965484739467644

SLC37A1 6 0 1 0.0416382254462463 1 0.887140871224982

MCM3AP 13 2 3 0.0226878877662642 0.975173805854432 0.899494782383858

CECR6 4 0 0 0.0463274983021047 1 1

ZC3H7B 9 2 1 0.0428170497206664 0.876968871613257 0.976958233729919

IL3RA 8 0 0 0.00203914068725113 1 1

FERD3L 6 0 1 0.0416382254462463 1 0.887140871224982

GALNT9 3 2 6 0.949811357358387 0.842068738489845 0.0429142007986453

FPGT-TNNI3K 8 2 9 0.737556972089228 0.981243578061332 0.0374715266911755

TMPRSS11B 6 0 1 0.0416382254462463 1 0.887140871224982

WDR37 6 0 1 0.0416382254462463 1 0.887140871224982

OR5M3 2 1 6 0.970319857406511 0.942028759099056 0.0126373532473112

ZNF417 3 0 5 0.809178333533066 1 0.0335780283108114

EPPIN 0 0 5 1 1 0.00122407013740092

ADAM28 7 0 1 0.0215160238576014 1 0.917693895037003

USP6NL 5 1 7 0.81044724850275 0.984085326617886 0.0309628117503546

FNDC3A 9 0 2 0.0179431022115431 1 0.837233316198664

SEZ6 8 1 1 0.0327516496754004 0.957970850346816 0.956360538099567

ZNF621 5 0 0 0.0213233416842564 1 1

MEF2A 7 0 1 0.0215160238576014 1 0.917693895037003

SIPA1L1 18 3 4 0.00728088933996523 0.982528269533504 0.936318216646134

TRIM55 5 2 7 0.866192167096457 0.926551100093222 0.0485352203130862

KRT31 2 1 6 0.970319857406511 0.942028759099056 0.0126373532473112

MYPN 6 2 11 0.945455953368343 0.981243578061332 0.00302248403681973

KRT84 1 1 5 0.988247552057685 0.890056051673045 0.015948402149609

CXCR2 2 0 4 0.85760924819628 1 0.0450453231390708

UBA3 1 1 4 0.977664195795495 0.848827071050437 0.0450453231390708

ITK 6 1 8 0.783532659638071 0.991715000861244 0.0223993563099247

ADGRA2 9 1 1 0.0179431022115431 0.969561061532513 0.968273260289657

PATL1 1 1 4 0.977664195795495 0.848827071050437 0.0450453231390708

PSMC4 0 0 4 1 1 0.0047530382994823

OR6K2 6 1 9 0.842024768729386 0.994031992145811 0.00980209135077389

RIC1 8 0 2 0.0327516496754004 1 0.793128963052146

CDH11 8 1 10 0.737556972089228 0.997784051416529 0.0117574264762433

TAOK2 12 0 5 0.0370537811471727 1 0.486876169292091

CEP135 9 2 1 0.0428170497206664 0.876968871613257 0.976958233729919

WNK2 16 3 5 0.0337541897751258 0.977420662701415 0.812900912715202

KIF21A 19 2 7 0.015647609100679 0.998676500832996 0.651876832457989

XKR7 8 0 1 0.0109413172055553 1 0.940037380594825

DAGLB 1 0 4 0.957655037862292 1 0.0188689109478078

SALL2 7 1 8 0.686045811152545 0.994031992145811 0.0349966212690756

CYP1A1 1 0 5 0.977664195795495 1 0.00578070485217632

OR3A1 2 0 5 0.914164058222356 1 0.015948402149609

LRRC4B 2 1 6 0.970319857406511 0.942028759099056 0.0126373532473112

TTLL2 11 0 1 0.00133495879775927 1 0.976958233729919

C14orf183 4 0 0 0.0463274983021047 1 1

ACE 13 2 3 0.0226878877662642 0.975173805854432 0.899494782383858

JSRP1 4 0 0 0.0463274983021047 1 1

FAM199X 6 1 0 0.0416382254462463 0.890056051673045 1

CASP9 4 0 0 0.0463274983021047 1 1

RALGDS 8 1 0 0.0109413172055553 0.942028759099056 1

ZSCAN20 5 2 7 0.866192167096457 0.926551100093222 0.0485352203130862

NRD1 4 1 9 0.953525872893264 0.988511036350553 0.00283038030605685

BMPER 7 0 7 0.50323080825299 1 0.0485352203130862

ILDR2 8 0 2 0.0327516496754004 1 0.793128963052146

PLXNA2 8 3 10 0.848761740459206 0.952433770201283 0.0271870539926272

MYLK 16 2 5 0.0192018764472082 0.99407304064624 0.779206397837303

DPPA2 7 0 1 0.0215160238576014 1 0.917693895037003

PHF14 9 1 2 0.0428170497206664 0.977978533343354 0.872738552446782

HEATR5A 8 0 9 0.581260271056406 1 0.0162256258908508

NOSTRIN 2 0 4 0.85760924819628 1 0.0450453231390708

RFC5 4 0 0 0.0463274983021047 1 1

FFAR3 9 1 1 0.0179431022115431 0.969561061532513 0.968273260289657

TTC22 7 0 1 0.0215160238576014 1 0.917693895037003

ATG4C 5 0 0 0.0213233416842564 1 1

BIVM-ERCC5 6 1 0 0.0416382254462463 0.890056051673045 1

SCAP 3 2 6 0.949811357358387 0.842068738489845 0.0429142007986453

PPP1R12B 8 0 1 0.0109413172055553 1 0.940037380594825

DPY19L3 5 0 0 0.0213233416842564 1 1

PDZD11 4 0 0 0.0463274983021047 1 1

ZNF732 3 0 6 0.875097348111752 1 0.0126373532473112

SPOCK2 0 1 4 1 0.792351146650411 0.0188689109478078

OR4C15 4 0 7 0.840736001584801 1 0.0097215538190752

EPB41L1 10 0 3 0.0247944696393595 1 0.716992894282767

ZBTB7B 3 1 6 0.920063533056499 0.957970850346816 0.0248091206279707

ZNF716 4 1 9 0.953525872893264 0.988511036350553 0.00283038030605685

EBF1 6 0 7 0.621445429270755 1 0.0309628117503546

ASAH2 6 0 1 0.0416382254462463 1 0.887140871224982

ZBTB48 6 0 1 0.0416382254462463 1 0.887140871224982

DEGS1 1 0 4 0.957655037862292 1 0.0188689109478078

MGAT5 5 0 7 0.737754241105828 1 0.0182515077914787

GPR160 6 0 1 0.0416382254462463 1 0.887140871224982

SCARB2 2 0 5 0.914164058222356 1 0.015948402149609

SPP1 2 0 4 0.85760924819628 1 0.0450453231390708

FRG2 1 0 4 0.957655037862292 1 0.0188689109478078

KIAA0141 8 0 1 0.0109413172055553 1 0.940037380594825

ZBTB22 2 1 5 0.949159299785454 0.920123595552516 0.0335780283108114

CA3 2 0 4 0.85760924819628 1 0.0450453231390708

DMRT3 1 0 6 0.988247552057685 1 0.00171362593320324

ZNF367 2 0 4 0.85760924819628 1 0.0450453231390708

OR1J2 0 1 4 1 0.792351146650411 0.0188689109478078

GLT6D1 3 0 5 0.809178333533066 1 0.0335780283108114

SLC15A3 2 0 6 0.949159299785454 1 0.0053706264528876

MTA2 6 0 7 0.621445429270755 1 0.0309628117503546

NCAM1 14 0 6 0.0266645160157442 1 0.446520862687304

ULK1 6 1 8 0.783532659638071 0.991715000861244 0.0223993563099247

LATS2 9 2 1 0.0428170497206664 0.876968871613257 0.976958233729919

RIPK3 2 0 4 0.85760924819628 1 0.0450453231390708

SYNE3 12 0 3 0.00783652701912318 1 0.809038983816177

SLC27A2 2 0 4 0.85760924819628 1 0.0450453231390708

LRRC28 1 0 4 0.957655037862292 1 0.0188689109478078

KIFC3 3 1 6 0.920063533056499 0.957970850346816 0.0248091206279707

CTU2 1 0 4 0.957655037862292 1 0.0188689109478078

ATP1B2 2 0 5 0.914164058222356 1 0.015948402149609

HOXB2 1 0 4 0.957655037862292 1 0.0188689109478078

ENTHD2 6 0 1 0.0416382254462463 1 0.887140871224982

SLC27A1 5 0 6 0.646696684471791 1 0.0429142007986453

UBA2 1 0 4 0.957655037862292 1 0.0188689109478078

RINL 1 0 4 0.957655037862292 1 0.0188689109478078

DYRK1B 2 1 6 0.970319857406511 0.942028759099056 0.0126373532473112

ADRA1D 1 1 4 0.977664195795495 0.848827071050437 0.0450453231390708

BRD1 8 0 9 0.581260271056406 1 0.0162256258908508

DDX53 2 0 4 0.85760924819628 1 0.0450453231390708

PSMD14 0 0 4 1 1 0.0047530382994823

KLHL24 4 1 6 0.840736001584801 0.969561061532513 0.0429142007986453

SUN1 4 1 6 0.840736001584801 0.969561061532513 0.0429142007986453

KIAA1549L 8 4 12 0.942193531558868 0.925524095291037 0.00957210628588356

FPR2 0 2 4 1 0.509971524650284 0.0450453231390708

CX3CL1 1 4 0 0.957655037862292 0.0196091101350379 1

SLC9A1 9 0 3 0.0428170497206664 1 0.659707134958072

SASS6 3 0 5 0.809178333533066 1 0.0335780283108114

CCDC181 2 0 4 0.85760924819628 1 0.0450453231390708

KCMF1 2 0 4 0.85760924819628 1 0.0450453231390708

NEUROD1 1 0 4 0.957655037862292 1 0.0188689109478078

NKX6-1 1 0 6 0.988247552057685 1 0.00171362593320324

TCF7 9 1 1 0.0179431022115431 0.969561061532513 0.968273260289657

DAXX 2 0 5 0.914164058222356 1 0.015948402149609

FHL5 2 1 5 0.949159299785454 0.920123595552516 0.0335780283108114

CBX3 1 1 4 0.977664195795495 0.848827071050437 0.0450453231390708

CNOT4 2 0 4 0.85760924819628 1 0.0450453231390708

BLK 3 0 5 0.809178333533066 1 0.0335780283108114

MTSS1 1 1 6 0.993831588096575 0.920123595552516 0.0053706264528876

SURF4 0 1 4 1 0.792351146650411 0.0188689109478078

UPF2 5 0 9 0.866192167096457 1 0.00283038030605685

OR10Q1 2 0 4 0.85760924819628 1 0.0450453231390708

SCYL1 2 0 6 0.949159299785454 1 0.0053706264528876

TAS2R8 1 0 5 0.977664195795495 1 0.00578070485217632

SPATS2 2 0 4 0.85760924819628 1 0.0450453231390708

TNS2 11 0 4 0.0312007424711093 1 0.596983435777183

MYO1H 3 1 7 0.949811357358387 0.969561061532513 0.0097215538190752

PAN3 3 0 6 0.875097348111752 1 0.0126373532473112

CDADC1 1 0 4 0.957655037862292 1 0.0188689109478078

RBM25 9 2 1 0.0428170497206664 0.876968871613257 0.976958233729919

NRXN3 7 4 10 0.932318426263042 0.862328475235952 0.0271870539926272

SEL1L 2 0 5 0.914164058222356 1 0.015948402149609

CD276 3 1 6 0.920063533056499 0.957970850346816 0.0248091206279707

IRX3 2 0 4 0.85760924819628 1 0.0450453231390708

ST8SIA3 3 0 6 0.875097348111752 1 0.0126373532473112

CYP4F3 8 0 1 0.0109413172055553 1 0.940037380594825

SRRM5 2 0 4 0.85760924819628 1 0.0450453231390708

KIAA1671 12 3 2 0.0370537811471727 0.879770846871731 0.965484739467644

IL17REL 6 0 1 0.0416382254462463 1 0.887140871224982

IRS4 8 5 11 0.942193531558868 0.820900615391716 0.0283125738357982

IPO7 10 0 3 0.0247944696393595 1 0.716992894282767

LRRC37B 4 0 0 0.0463274983021047 1 1

TMEM201 1 4 1 0.977664195795495 0.0466863691393309 0.845404287753466

CLCN6 8 1 1 0.0327516496754004 0.957970850346816 0.956360538099567

ST6GALNAC3 8 0 2 0.0327516496754004 1 0.793128963052146

PKN2 9 0 3 0.0428170497206664 1 0.659707134958072

FAM212B 4 0 0 0.0463274983021047 1 1

LRIG2 2 0 5 0.914164058222356 1 0.015948402149609

CTSS 4 0 0 0.0463274983021047 1 1

RORC 5 0 0 0.0213233416842564 1 1

POGK 8 0 2 0.0327516496754004 1 0.793128963052146

SLC26A9 9 1 1 0.0179431022115431 0.969561061532513 0.968273260289657

PPP2R5A 3 0 5 0.809178333533066 1 0.0335780283108114

SUSD4 6 0 1 0.0416382254462463 1 0.887140871224982

ALLC 6 0 0 0.00978296170678797 1 1

PREB 7 0 0 0.00447375108354491 1 1

FEZ2 4 0 0 0.0463274983021047 1 1

FBXO41 9 0 1 0.00548873408809398 1 0.956360538099567

BCL2L11 5 0 0 0.0213233416842564 1 1

ZRANB3 7 0 8 0.599966998128091 1 0.0223993563099247

DNAJC10 9 2 1 0.0428170497206664 0.876968871613257 0.976958233729919

C2orf66 4 0 0 0.0463274983021047 1 1

GPC1 6 0 0 0.00978296170678797 1 1

SUMF1 4 0 0 0.0463274983021047 1 1

TMEM43 4 0 0 0.0463274983021047 1 1

SGOL1 10 1 1 0.00965178637516871 0.977978533343354 0.976958233729919

IFRD2 2 1 5 0.949159299785454 0.920123595552516 0.0335780283108114

ITIH4 5 0 0 0.0213233416842564 1 1

PRKCD 8 0 2 0.0327516496754004 1 0.793128963052146

COPG1 6 0 0 0.00978296170678797 1 1

STIM2 1 1 6 0.993831588096575 0.920123595552516 0.0053706264528876

ARSJ 6 1 0 0.0416382254462463 0.890056051673045 1

GALNTL6 9 0 2 0.0179431022115431 1 0.837233316198664

NKD2 5 0 0 0.0213233416842564 1 1

IQGAP2 11 0 3 0.0140669844729861 1 0.766627874208378

SMAD5 4 0 0 0.0463274983021047 1 1

IK 5 0 0 0.0213233416842564 1 1

PPARGC1B 10 0 2 0.00965178637516871 1 0.872738552446782

HMGXB3 9 0 3 0.0428170497206664 1 0.659707134958072

SENP6 11 2 2 0.0312007424711093 0.943655533200879 0.941063952426992

AEBP1 4 0 8 0.892226881824287 1 0.00362872955307272

DTX2 4 0 0 0.0463274983021047 1 1

PON2 6 0 0 0.00978296170678797 1 1

MEPCE 7 0 1 0.0215160238576014 1 0.917693895037003

CHPF2 7 1 0 0.0215160238576014 0.920123595552516 1

KCTD9 7 0 1 0.0215160238576014 1 0.917693895037003

HMBOX1 4 0 0 0.0463274983021047 1 1

SPIDR 2 1 5 0.949159299785454 0.920123595552516 0.0335780283108114

SLC52A2 4 0 0 0.0463274983021047 1 1

KIAA1161 8 0 1 0.0109413172055553 1 0.940037380594825

TRIM14 4 0 0 0.0463274983021047 1 1

FKBP15 10 1 2 0.0247944696393595 0.984085326617886 0.90105594484024

CIZ1 9 0 3 0.0428170497206664 1 0.659707134958072

KCNT1 9 1 0 0.00548873408809398 0.957970850346816 1

INPP5E 6 0 1 0.0416382254462463 1 0.887140871224982

NSMF 4 0 0 0.0463274983021047 1 1

CUEDC2 4 0 0 0.0463274983021047 1 1

MRPL16 5 0 0 0.0213233416842564 1 1

NXF1 7 0 1 0.0215160238576014 1 0.917693895037003

SYVN1 9 0 3 0.0428170497206664 1 0.659707134958072

B4GAT1 6 0 0 0.00978296170678797 1 1

MMP27 4 0 0 0.0463274983021047 1 1

P3H3 11 2 2 0.0312007424711093 0.943655533200879 0.941063952426992

KRT83 5 0 0 0.0213233416842564 1 1

ITGB7 6 0 1 0.0416382254462463 1 0.887140871224982

SDS 5 0 0 0.0213233416842564 1 1

NRL 4 0 0 0.0463274983021047 1 1

DCAF4 1 0 4 0.957655037862292 1 0.0188689109478078

FAM161B 8 0 2 0.0327516496754004 1 0.793128963052146

LIPC 6 0 1 0.0416382254462463 1 0.887140871224982

ALPK3 14 1 5 0.0266645160157442 0.998410848301568 0.651585258628802

WDR90 9 2 1 0.0428170497206664 0.876968871613257 0.976958233729919

CHTF18 10 1 2 0.0247944696393595 0.984085326617886 0.90105594484024

CIITA 8 2 9 0.737556972089228 0.981243578061332 0.0374715266911755

RABEP2 5 0 0 0.0213233416842564 1 1

BCKDK 7 0 1 0.0215160238576014 1 0.917693895037003

ZNF319 6 0 1 0.0416382254462463 1 0.887140871224982

WDR59 8 1 1 0.0327516496754004 0.957970850346816 0.956360538099567

ZNRF1 4 0 0 0.0463274983021047 1 1

CDK10 5 0 0 0.0213233416842564 1 1

SPATA2L 4 0 0 0.0463274983021047 1 1

NUP88 7 0 0 0.00447375108354491 1 1

PIK3R5 10 0 1 0.00272109672732751 1 0.968273260289657

DDX52 6 1 0 0.0416382254462463 0.890056051673045 1

KRTAP1-5 4 0 0 0.0463274983021047 1 1

COASY 4 0 0 0.0463274983021047 1 1

NSF 6 0 1 0.0416382254462463 1 0.887140871224982

WFIKKN2 6 0 1 0.0416382254462463 1 0.887140871224982

USH1G 6 0 1 0.0416382254462463 1 0.887140871224982

CYGB 5 0 0 0.0213233416842564 1 1

ZNF236 12 1 4 0.0370537811471727 0.995705739391037 0.705280176200014

HMHA1 9 1 2 0.0428170497206664 0.977978533343354 0.872738552446782

ZNF563 5 0 0 0.0213233416842564 1 1

IRGQ 6 0 0 0.00978296170678797 1 1

KLC3 7 0 0 0.00447375108354491 1 1

KCNJ14 4 0 0 0.0463274983021047 1 1

KCNC3 11 1 2 0.0140669844729861 0.988511036350553 0.923460623278883

FAM71E2 8 0 0 0.00203914068725113 1 1

SAMHD1 9 2 1 0.0428170497206664 0.876968871613257 0.976958233729919

TSPEAR 6 1 0 0.0416382254462463 0.890056051673045 1

C21orf58 6 0 0 0.00978296170678797 1 1

GAS2L1 9 0 1 0.00548873408809398 1 0.956360538099567

SUN2 10 0 2 0.00965178637516871 1 0.872738552446782

PRRG1 5 0 0 0.0213233416842564 1 1

UBA1 6 0 1 0.0416382254462463 1 0.887140871224982

DLG3 6 0 1 0.0416382254462463 1 0.887140871224982

GRIA3 3 1 6 0.920063533056499 0.957970850346816 0.0248091206279707

TNFRSF9 4 0 0 0.0463274983021047 1 1

IQSEC1 9 1 2 0.0428170497206664 0.977978533343354 0.872738552446782

QARS 7 0 1 0.0215160238576014 1 0.917693895037003

NAT8L 1 0 4 0.957655037862292 1 0.0188689109478078

PAPD7 5 0 0 0.0213233416842564 1 1

ESRP1 3 3 7 0.981136021640521 0.724464088216835 0.0309628117503546

DENND1A 7 0 1 0.0215160238576014 1 0.917693895037003

LHPP 7 0 1 0.0215160238576014 1 0.917693895037003

RPL10L 4 0 0 0.0463274983021047 1 1

DACT1 10 0 2 0.00965178637516871 1 0.872738552446782

MIEF2 6 0 1 0.0416382254462463 1 0.887140871224982

TOP3A 5 0 6 0.646696684471791 1 0.0429142007986453

DCAF7 2 0 4 0.85760924819628 1 0.0450453231390708

MAPK8IP2 5 0 0 0.0213233416842564 1 1

SLC25A46 2 0 4 0.85760924819628 1 0.0450453231390708

RARS2 7 0 1 0.0215160238576014 1 0.917693895037003

NR5A1 7 1 0 0.0215160238576014 0.920123595552516 1

ZNF556 9 1 1 0.0179431022115431 0.969561061532513 0.968273260289657

PRAMEF8 0 0 6 1 1 0.00031274319444584

MAST2 7 2 9 0.819617606155775 0.975173805854432 0.0252911634314708

IFI44 1 0 5 0.977664195795495 1 0.00578070485217632

TOR1AIP1 1 0 4 0.957655037862292 1 0.0188689109478078

CDC42BPA 10 2 10 0.628378023831211 0.992060914657446 0.038811649179659

LYG1 2 0 4 0.85760924819628 1 0.0450453231390708

ARHGEF4 3 0 6 0.875097348111752 1 0.0126373532473112

GTDC1 9 0 2 0.0179431022115431 1 0.837233316198664

USP37 1 0 4 0.957655037862292 1 0.0188689109478078

FAM124B 2 0 4 0.85760924819628 1 0.0450453231390708

AASDH 11 0 2 0.00510898139591144 1 0.90105594484024

G3BP1 6 1 7 0.710106395034079 0.988511036350553 0.0485352203130862

CUL7 6 1 7 0.710106395034079 0.988511036350553 0.0485352203130862

PLEKHG1 6 1 7 0.710106395034079 0.988511036350553 0.0485352203130862

PARK2 1 0 5 0.977664195795495 1 0.00578070485217632

STEAP2 1 0 4 0.957655037862292 1 0.0188689109478078

KRIT1 2 1 5 0.949159299785454 0.920123595552516 0.0335780283108114

ZNF862 8 2 10 0.798809373946043 0.985874910600787 0.0182961548166117

PURG 4 1 6 0.840736001584801 0.969561061532513 0.0429142007986453

LSM1 1 0 4 0.957655037862292 1 0.0188689109478078

VCPIP1 4 2 8 0.953525872893264 0.926551100093222 0.0133904031876229

OR13C9 0 1 5 1 0.848827071050437 0.00578070485217632

LRRC8A 0 1 4 1 0.792351146650411 0.0188689109478078

ZC3H12C 3 0 5 0.809178333533066 1 0.0335780283108114

NXPE4 0 1 5 1 0.848827071050437 0.00578070485217632

LMNTD1 1 1 4 0.977664195795495 0.848827071050437 0.0450453231390708

ABCD2 3 1 6 0.920063533056499 0.957970850346816 0.0248091206279707

ASB8 0 0 4 1 1 0.0047530382994823

METTL1 1 0 4 0.957655037862292 1 0.0188689109478078

DUSP6 2 0 4 0.85760924819628 1 0.0450453231390708

ERO1L 0 0 4 1 1 0.0047530382994823

SERPINA6 1 0 4 0.957655037862292 1 0.0188689109478078

IGHG4 1 2 5 0.993831588096575 0.679583244516747 0.0335780283108114

MEIOB 2 0 5 0.914164058222356 1 0.015948402149609

CMTR2 2 0 5 0.914164058222356 1 0.015948402149609

CEP95 8 0 1 0.0109413172055553 1 0.940037380594825

OTOP2 10 0 3 0.0247944696393595 1 0.716992894282767

RALBP1 2 0 4 0.85760924819628 1 0.0450453231390708

PGLYRP1 0 0 4 1 1 0.0047530382994823

AURKC 2 0 4 0.85760924819628 1 0.0450453231390708

ZNF584 1 0 4 0.957655037862292 1 0.0188689109478078

LRRC74B 1 0 5 0.977664195795495 1 0.00578070485217632

ENTHD1 3 1 6 0.920063533056499 0.957970850346816 0.0248091206279707

TNMD 2 0 4 0.85760924819628 1 0.0450453231390708

HTR2C 4 4 8 0.981241659115817 0.663051026780764 0.0349966212690756

FMR1NB 1 1 4 0.977664195795495 0.848827071050437 0.0450453231390708

KRTAP19-3 4 0 0 0.0463274983021047 1 1

GLYR1 2 0 4 0.85760924819628 1 0.0450453231390708

KLHL18 1 1 4 0.977664195795495 0.848827071050437 0.0450453231390708

CCDC136 9 1 2 0.0428170497206664 0.977978533343354 0.872738552446782

TIGD3 1 1 4 0.977664195795495 0.848827071050437 0.0450453231390708

HINFP 2 1 5 0.949159299785454 0.920123595552516 0.0335780283108114

OR7E24 2 1 5 0.949159299785454 0.920123595552516 0.0335780283108114

BTNL8 7 1 0 0.0215160238576014 0.920123595552516 1

NHLRC2 2 6 2 0.982883938494014 0.0260985948535137 0.793128963052146

MYOD1 1 1 4 0.977664195795495 0.848827071050437 0.0450453231390708

OR2T27 3 0 5 0.809178333533066 1 0.0335780283108114

OR5L1 1 1 4 0.977664195795495 0.848827071050437 0.0450453231390708

TEX13B 2 0 4 0.85760924819628 1 0.0450453231390708

CPSF3L 6 1 0 0.0416382254462463 0.890056051673045 1

KDF1 5 0 0 0.0213233416842564 1 1

IFI44L 2 0 4 0.85760924819628 1 0.0450453231390708

ADCK3 6 0 1 0.0416382254462463 1 0.887140871224982

ZNF670 4 0 0 0.0463274983021047 1 1

PROC 6 0 1 0.0416382254462463 1 0.887140871224982

DCLK3 9 0 3 0.0428170497206664 1 0.659707134958072

PPP1R2 6 0 0 0.00978296170678797 1 1

OR12D2 7 0 0 0.00447375108354491 1 1

KLHL31 6 1 0 0.0416382254462463 0.890056051673045 1

SLC16A10 7 1 0 0.0215160238576014 0.920123595552516 1

PEG10 4 0 0 0.0463274983021047 1 1

TSGA13 4 0 0 0.0463274983021047 1 1

AOC1 10 0 1 0.00272109672732751 1 0.968273260289657

ACTL7A 7 0 0 0.00447375108354491 1 1

SAPCD2 4 0 0 0.0463274983021047 1 1

BUB3 4 0 0 0.0463274983021047 1 1

SLC22A18 4 0 0 0.0463274983021047 1 1

CTSC 10 0 2 0.00965178637516871 1 0.872738552446782

EIF3J 8 0 1 0.0109413172055553 1 0.940037380594825

FURIN 5 2 8 0.90752192082565 0.943655533200879 0.0223993563099247

NFATC2IP 4 0 0 0.0463274983021047 1 1

MARVELD3 6 1 0 0.0416382254462463 0.890056051673045 1

PROCA1 3 0 5 0.809178333533066 1 0.0335780283108114

ZNF750 6 1 0 0.0416382254462463 0.890056051673045 1

MPPE1 5 0 0 0.0213233416842564 1 1

KEAP1 5 0 0 0.0213233416842564 1 1

CLIP3 9 0 2 0.0179431022115431 1 0.837233316198664

PPFIA3 9 1 1 0.0179431022115431 0.969561061532513 0.968273260289657

TMEM52B 1 1 5 0.988247552057685 0.890056051673045 0.015948402149609

MFN2 8 1 1 0.0327516496754004 0.957970850346816 0.956360538099567

FAM214B 5 2 7 0.866192167096457 0.926551100093222 0.0485352203130862

KLHL13 1 1 5 0.988247552057685 0.890056051673045 0.015948402149609

CIART 5 0 0 0.0213233416842564 1 1

PHAX 7 0 1 0.0215160238576014 1 0.917693895037003

DLGAP4 10 1 2 0.0247944696393595 0.984085326617886 0.90105594484024

NECAP2 4 0 0 0.0463274983021047 1 1

NUDT17 4 0 0 0.0463274983021047 1 1

KCNJ11 5 0 0 0.0213233416842564 1 1

GIF 6 0 1 0.0416382254462463 1 0.887140871224982

TMC7 6 0 1 0.0416382254462463 1 0.887140871224982

ASPHD2 6 0 1 0.0416382254462463 1 0.887140871224982

PATZ1 4 0 0 0.0463274983021047 1 1

ZCCHC5 8 0 2 0.0327516496754004 1 0.793128963052146

PTGFR 6 1 0 0.0416382254462463 0.890056051673045 1

FMO5 9 0 0 0.000926368622463118 1 1

KIFAP3 8 0 2 0.0327516496754004 1 0.793128963052146

MR1 6 0 1 0.0416382254462463 1 0.887140871224982

WNT9A 8 0 1 0.0109413172055553 1 0.940037380594825

OR2L13 2 1 5 0.949159299785454 0.920123595552516 0.0335780283108114

CKAP2L 2 0 4 0.85760924819628 1 0.0450453231390708

MOGAT1 5 0 0 0.0213233416842564 1 1

LRCH3 6 0 1 0.0416382254462463 1 0.887140871224982

BTN2A1 7 0 1 0.0215160238576014 1 0.917693895037003

BNIP3L 4 0 0 0.0463274983021047 1 1

IDO2 6 0 0 0.00978296170678797 1 1

DCAF12 5 0 0 0.0213233416842564 1 1

ZNF169 2 0 4 0.85760924819628 1 0.0450453231390708

ERP44 7 0 1 0.0215160238576014 1 0.917693895037003

ZDHHC6 4 0 0 0.0463274983021047 1 1

SDHAF2 4 0 0 0.0463274983021047 1 1

FOLR2 4 0 0 0.0463274983021047 1 1

STARD13 3 1 7 0.949811357358387 0.969561061532513 0.0097215538190752

HIF1A 8 0 1 0.0109413172055553 1 0.940037380594825

IGHV1-46 4 0 0 0.0463274983021047 1 1

LYSMD2 4 0 0 0.0463274983021047 1 1

TEX9 4 0 0 0.0463274983021047 1 1

ZNF592 8 1 1 0.0327516496754004 0.957970850346816 0.956360538099567

MYO5B 9 0 2 0.0179431022115431 1 0.837233316198664

FBXO15 1 0 4 0.957655037862292 1 0.0188689109478078

ZSCAN4 6 0 1 0.0416382254462463 1 0.887140871224982

TTI1 10 0 2 0.00965178637516871 1 0.872738552446782

FAM217B 4 0 0 0.0463274983021047 1 1

TCEANC 5 0 0 0.0213233416842564 1 1

TFDP3 4 0 0 0.0463274983021047 1 1

FAM122C 4 0 0 0.0463274983021047 1 1

TYK2 4 1 6 0.840736001584801 0.969561061532513 0.0429142007986453

TMEM63C 8 2 0 0.0327516496754004 0.79854993786922 1

TMEM171 1 0 4 0.957655037862292 1 0.0188689109478078

KCNK5 1 0 4 0.957655037862292 1 0.0188689109478078

ZSCAN2 2 0 4 0.85760924819628 1 0.0450453231390708

RHOXF2 1 0 5 0.977664195795495 1 0.00578070485217632

ZNF385B 7 0 1 0.0215160238576014 1 0.917693895037003

POU4F2 7 2 9 0.819617606155775 0.975173805854432 0.0252911634314708

TMEM109 1 1 4 0.977664195795495 0.848827071050437 0.0450453231390708

KHDRBS1 6 1 0 0.0416382254462463 0.890056051673045 1

CD207 6 0 1 0.0416382254462463 1 0.887140871224982

GLI4 6 0 1 0.0416382254462463 1 0.887140871224982

SLC27A4 5 0 0 0.0213233416842564 1 1

MAMDC4 6 0 1 0.0416382254462463 1 0.887140871224982

RPL6 4 0 0 0.0463274983021047 1 1

KRT23 6 0 0 0.00978296170678797 1 1

REXO1 3 0 6 0.875097348111752 1 0.0126373532473112

ZNF358 1 0 4 0.957655037862292 1 0.0188689109478078

ARHGAP40 4 0 0 0.0463274983021047 1 1

SLC25A18 1 0 4 0.957655037862292 1 0.0188689109478078

DMAP1 2 0 4 0.85760924819628 1 0.0450453231390708

AAK1 9 0 0 0.000926368622463118 1 1

ZYX 4 0 0 0.0463274983021047 1 1

OR52L1 4 0 0 0.0463274983021047 1 1

STPG1 4 0 0 0.0463274983021047 1 1

XRCC5 8 1 1 0.0327516496754004 0.957970850346816 0.956360538099567

HHLA2 1 0 4 0.957655037862292 1 0.0188689109478078

B4GALT7 4 0 0 0.0463274983021047 1 1

CENPQ 4 0 0 0.0463274983021047 1 1

TAF6 4 0 0 0.0463274983021047 1 1

ZNF25 2 0 4 0.85760924819628 1 0.0450453231390708

CCDC59 4 0 0 0.0463274983021047 1 1

ADGRE5 9 0 3 0.0428170497206664 1 0.659707134958072

HSCB 4 0 0 0.0463274983021047 1 1

COA1 4 0 0 0.0463274983021047 1 1

MSRB3 5 0 0 0.0213233416842564 1 1

CSNK2A2 8 0 1 0.0109413172055553 1 0.940037380594825

YARS 5 0 0 0.0213233416842564 1 1

FAM71A 7 0 1 0.0215160238576014 1 0.917693895037003

POMGNT2 8 0 2 0.0327516496754004 1 0.793128963052146

ACSL1 6 0 1 0.0416382254462463 1 0.887140871224982

PHKG1 6 0 0 0.00978296170678797 1 1

GPR137 6 0 0 0.00978296170678797 1 1

LBP 5 0 0 0.0213233416842564 1 1

MED14 11 1 1 0.00510898139591144 0.984085326617886 0.983283424470725

FAM63A 0 0 4 1 1 0.0047530382994823

LMOD1 2 0 6 0.949159299785454 1 0.0053706264528876

CAMK2G 4 2 7 0.928573385522747 0.90469701404897 0.0309628117503546

RGS10 0 0 4 1 1 0.0047530382994823

MUC15 1 0 4 0.957655037862292 1 0.0188689109478078

ARHGDIB 2 0 4 0.85760924819628 1 0.0450453231390708

ZMYND15 4 0 6 0.770176249907343 1 0.0248091206279707

CRLF3 1 0 4 0.957655037862292 1 0.0188689109478078

ERN1 3 0 5 0.809178333533066 1 0.0335780283108114

MTMR8 0 0 4 1 1 0.0047530382994823

LUZP4 2 0 5 0.914164058222356 1 0.015948402149609

LECT1 4 0 0 0.0463274983021047 1 1

RCC2 8 0 1 0.0109413172055553 1 0.940037380594825

C1QL2 5 0 0 0.0213233416842564 1 1

MBNL2 5 0 0 0.0213233416842564 1 1

CSTF2 4 0 0 0.0463274983021047 1 1

LRRC42 2 0 4 0.85760924819628 1 0.0450453231390708

ACVR1 2 0 4 0.85760924819628 1 0.0450453231390708

GSTA3 1 0 4 0.957655037862292 1 0.0188689109478078

DBNL 1 0 4 0.957655037862292 1 0.0188689109478078

KAT5 1 0 4 0.957655037862292 1 0.0188689109478078

TBC1D24 2 0 4 0.85760924819628 1 0.0450453231390708

IGSF8 1 0 5 0.977664195795495 1 0.00578070485217632

ZNF852 1 1 5 0.988247552057685 0.890056051673045 0.015948402149609

ZNF793 5 0 6 0.646696684471791 1 0.0429142007986453

ZNF80 5 0 0 0.0213233416842564 1 1

RHCE 1 0 4 0.957655037862292 1 0.0188689109478078

ATP6V1C2 4 0 0 0.0463274983021047 1 1

CAV3 4 0 0 0.0463274983021047 1 1

SPRY4 5 0 0 0.0213233416842564 1 1

WDR89 4 0 0 0.0463274983021047 1 1

WBP2 4 0 0 0.0463274983021047 1 1

SOCS6 6 0 1 0.0416382254462463 1 0.887140871224982

GCDH 9 0 1 0.00548873408809398 1 0.956360538099567

DEDD2 4 0 0 0.0463274983021047 1 1

PPP1R13L 2 0 5 0.914164058222356 1 0.015948402149609

C5AR1 4 0 0 0.0463274983021047 1 1

SBK2 4 0 0 0.0463274983021047 1 1

NLRP11 9 0 2 0.0179431022115431 1 0.837233316198664

BFSP1 6 1 0 0.0416382254462463 0.890056051673045 1

TST 5 0 0 0.0213233416842564 1 1

FRS3 7 1 0 0.0215160238576014 0.920123595552516 1

SCNM1 4 0 0 0.0463274983021047 1 1

ACBD3 4 0 0 0.0463274983021047 1 1

FAM198A 4 0 0 0.0463274983021047 1 1

SLC35G2 5 0 0 0.0213233416842564 1 1

ADH1C 4 0 0 0.0463274983021047 1 1

RAI14 8 0 1 0.0109413172055553 1 0.940037380594825

STXBP1 6 0 1 0.0416382254462463 1 0.887140871224982

IDI2 4 0 0 0.0463274983021047 1 1

TSSK4 4 0 0 0.0463274983021047 1 1

LPAR4 6 1 0 0.0416382254462463 0.890056051673045 1

CD1C 4 0 0 0.0463274983021047 1 1

SLC1A4 6 0 0 0.00978296170678797 1 1

HOXD1 4 0 0 0.0463274983021047 1 1

UBE2K 5 0 0 0.0213233416842564 1 1

ABT1 6 0 1 0.0416382254462463 1 0.887140871224982

NCAPG2 6 1 0 0.0416382254462463 0.890056051673045 1

TRIM32 8 1 0 0.0109413172055553 0.942028759099056 1

HPX 6 0 0 0.00978296170678797 1 1

KBTBD4 6 0 1 0.0416382254462463 1 0.887140871224982

DGCR14 5 0 0 0.0213233416842564 1 1

OR14C36 0 0 4 1 1 0.0047530382994823

C11orf42 6 0 0 0.00978296170678797 1 1

DEFB116 2 0 4 0.85760924819628 1 0.0450453231390708

CATSPER2 2 0 4 0.85760924819628 1 0.0450453231390708

MKNK2 5 0 0 0.0213233416842564 1 1

MICALCL 2 0 4 0.85760924819628 1 0.0450453231390708

ME2 4 0 7 0.840736001584801 1 0.0097215538190752

FMO4 3 0 5 0.809178333533066 1 0.0335780283108114

CXCR4 1 0 6 0.988247552057685 1 0.00171362593320324

ANXA5 0 0 4 1 1 0.0047530382994823

EDIL3 2 1 7 0.982883938494014 0.957970850346816 0.00452284166020484

AMD1 1 0 4 0.957655037862292 1 0.0188689109478078

RASSF4 1 0 4 0.957655037862292 1 0.0188689109478078

HOXC12 0 0 4 1 1 0.0047530382994823

PTPRH 4 1 6 0.840736001584801 0.969561061532513 0.0429142007986453

RCC1 1 0 4 0.957655037862292 1 0.0188689109478078

HGFAC 2 0 5 0.914164058222356 1 0.015948402149609

API5 1 0 5 0.977664195795495 1 0.00578070485217632

ADCK1 2 0 5 0.914164058222356 1 0.015948402149609

C9orf3 6 0 1 0.0416382254462463 1 0.887140871224982

TMPRSS5 5 0 0 0.0213233416842564 1 1

IPP 4 0 0 0.0463274983021047 1 1

PPM1H 7 0 1 0.0215160238576014 1 0.917693895037003

JPH2 2 1 6 0.970319857406511 0.942028759099056 0.0126373532473112

OSBPL2 6 0 1 0.0416382254462463 1 0.887140871224982

CCDC78 0 1 4 1 0.792351146650411 0.0188689109478078

ZNF141 2 0 4 0.85760924819628 1 0.0450453231390708

STAM 0 0 4 1 1 0.0047530382994823

PIP4K2B 1 1 4 0.977664195795495 0.848827071050437 0.0450453231390708

ALAS2 7 0 1 0.0215160238576014 1 0.917693895037003

SECISBP2 4 0 0 0.0463274983021047 1 1

CLYBL 5 0 0 0.0213233416842564 1 1

NUDT14 4 0 0 0.0463274983021047 1 1

SMARCE1 4 0 0 0.0463274983021047 1 1

WTIP 4 0 0 0.0463274983021047 1 1

TROVE2 6 1 0 0.0416382254462463 0.890056051673045 1

BASP1 4 0 0 0.0463274983021047 1 1

SLC41A2 4 0 0 0.0463274983021047 1 1

MMADHC 1 0 4 0.957655037862292 1 0.0188689109478078

PIP5K1A 1 0 4 0.957655037862292 1 0.0188689109478078

OR10G8 6 0 0 0.00978296170678797 1 1

CCDC112 5 0 0 0.0213233416842564 1 1

VSTM2A 6 0 1 0.0416382254462463 1 0.887140871224982

MRPS2 6 0 0 0.00978296170678797 1 1

FGF3 1 0 4 0.957655037862292 1 0.0188689109478078

C1GALT1 5 0 0 0.0213233416842564 1 1

GBX1 4 0 0 0.0463274983021047 1 1

C11orf49 4 0 0 0.0463274983021047 1 1

ACSM3 6 0 0 0.00978296170678797 1 1

RARA 6 0 1 0.0416382254462463 1 0.887140871224982

RSPO4 4 0 0 0.0463274983021047 1 1

SIRT1 6 0 1 0.0416382254462463 1 0.887140871224982

HNRNPCL2 4 0 0 0.0463274983021047 1 1

MRPS10 4 0 0 0.0463274983021047 1 1

FAM220A 7 0 0 0.00447375108354491 1 1

TAS2R38 5 0 0 0.0213233416842564 1 1

PMP22 4 0 0 0.0463274983021047 1 1

GNL3 1 0 5 0.977664195795495 1 0.00578070485217632

CTH 4 0 0 0.0463274983021047 1 1

CTSL 4 0 0 0.0463274983021047 1 1

SHPK 4 0 0 0.0463274983021047 1 1

MCM5 4 0 0 0.0463274983021047 1 1

P2RY14 1 0 4 0.957655037862292 1 0.0188689109478078

TATDN3 4 0 0 0.0463274983021047 1 1

DNAJC25 4 0 0 0.0463274983021047 1 1

ACPT 5 0 0 0.0213233416842564 1 1

PLEKHA1 4 0 0 0.0463274983021047 1 1

FBXO28 4 0 0 0.0463274983021047 1 1

CCDC50 4 0 0 0.0463274983021047 1 1

TSNAXIP1 4 0 0 0.0463274983021047 1 1

DNAJA3 0 0 4 1 1 0.0047530382994823

MYLIP 4 0 0 0.0463274983021047 1 1

FAM78A 4 0 0 0.0463274983021047 1 1

ACAT1 4 0 0 0.0463274983021047 1 1

PID1 4 0 0 0.0463274983021047 1 1

ZNF165 1 0 4 0.957655037862292 1 0.0188689109478078

YBX1 4 0 0 0.0463274983021047 1 1

IGLV1-50 0 0 4 1 1 0.0047530382994823
