# Supplementary figures and images for: Identification and Validation of Immune Molecular Subtypes and Immune Landscape Based on Colon Cancer Cohort
Source: Front Med (Lausanne). 2022 May 6;9:827695. doi: 10.3389/fmed.2022.827695 (PMC9121983; doi:10.3389/fmed.2022.827695)

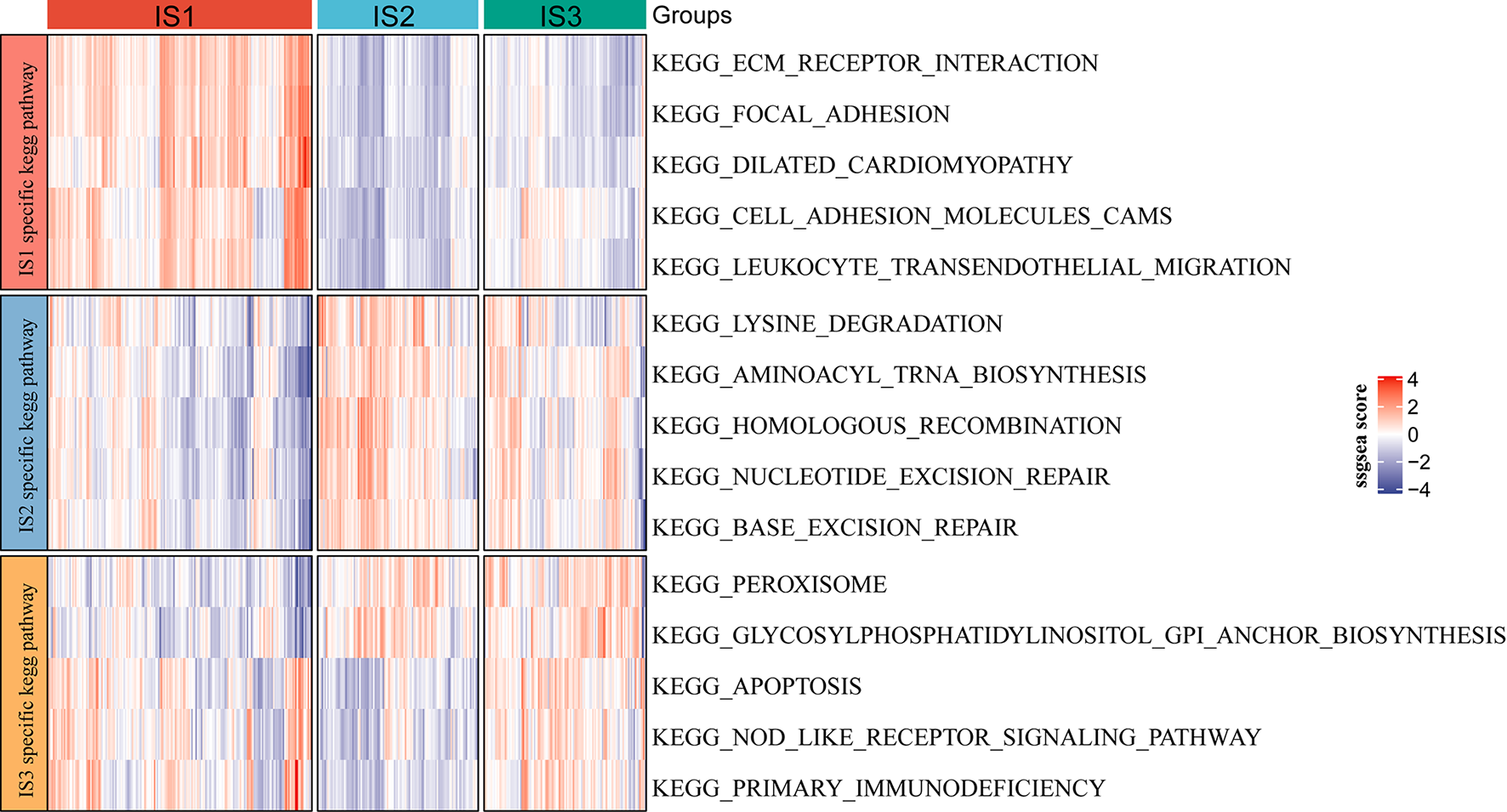

Supplement: Supplementary file 4 [file Image_1.TIF]
